# Chromosome-level reference genome of the European wasp spider *Argiope bruennichi*: a resource for studies on range expansion and evolutionary adaptation

**DOI:** 10.1093/gigascience/giaa148

**Published:** 2021-01-07

**Authors:** Monica M Sheffer, Anica Hoppe, Henrik Krehenwinkel, Gabriele Uhl, Andreas W Kuss, Lars Jensen, Corinna Jensen, Rosemary G Gillespie, Katharina J Hoff, Stefan Prost

**Affiliations:** Zoological Institute and Museum, University of Greifswald, Loitzer Str. 26, 17489 Greifswald, Germany; Institute of Mathematics and Computer Science, University of Greifswald, Walther-Rathenau-Str. 47, 17489 Greifswald, Germany; Center for Functional Genomics of Microbes, University of Greifswald, Felix-Hausdorf-Str. 8, 17489 Greifswald, Germany; Department of Biogeography, University of Trier, Universitätsring 15, 54296 Trier, Germany; Zoological Institute and Museum, University of Greifswald, Loitzer Str. 26, 17489 Greifswald, Germany; Center for Functional Genomics of Microbes, University of Greifswald, Felix-Hausdorf-Str. 8, 17489 Greifswald, Germany; Interfaculty Institute for Genetics and Functional Genomics, University of Greifswald, Felix-Hausdorf-Str. 8, 17489 Greifswald, Germany; Center for Functional Genomics of Microbes, University of Greifswald, Felix-Hausdorf-Str. 8, 17489 Greifswald, Germany; Interfaculty Institute for Genetics and Functional Genomics, University of Greifswald, Felix-Hausdorf-Str. 8, 17489 Greifswald, Germany; Center for Functional Genomics of Microbes, University of Greifswald, Felix-Hausdorf-Str. 8, 17489 Greifswald, Germany; Interfaculty Institute for Genetics and Functional Genomics, University of Greifswald, Felix-Hausdorf-Str. 8, 17489 Greifswald, Germany; Department of Environmental Science Policy and Management, University of California Berkeley, 130 Mulford Hall #3114, Berkeley, CA, 94720, USA; Institute of Mathematics and Computer Science, University of Greifswald, Walther-Rathenau-Str. 47, 17489 Greifswald, Germany; Center for Functional Genomics of Microbes, University of Greifswald, Felix-Hausdorf-Str. 8, 17489 Greifswald, Germany; LOEWE-Centre for Translational Biodiversity Genomics, Senckenberganlage 25, 60325 Frankfurt, Germany; South African National Biodiversity Institute, National Zoological Gardens of South Africa, 232 Boom St., Pretoria 0001, South Africa

**Keywords:** Argiope bruennichi, genome assembly, Araneae, spider, PacBio, Hi-C, chromosome-level, Hox duplication, silk, venom

## Abstract

**Background:**

*Argiope bruennichi*, the European wasp spider, has been investigated intensively as a focal species for studies on sexual selection, chemical communication, and the dynamics of rapid range expansion at a behavioral and genetic level. However, the lack of a reference genome has limited insights into the genetic basis for these phenomena. Therefore, we assembled a high-quality chromosome-level reference genome of the European wasp spider as a tool for more in-depth future studies.

**Findings:**

We generated, *de novo*, a 1.67 Gb genome assembly of *A. bruennichi* using 21.8× Pacific Biosciences sequencing, polished with 19.8× Illumina paired-end sequencing data, and proximity ligation (Hi-C)-based scaffolding. This resulted in an N50 scaffold size of 124 Mb and an N50 contig size of 288 kb. We found 98.4% of the genome to be contained in 13 scaffolds, fitting the expected number of chromosomes (n = 13). Analyses showed the presence of 91.1% of complete arthropod BUSCOs, indicating a high-quality assembly.

**Conclusions:**

We present the first chromosome-level genome assembly in the order Araneae. With this genomic resource, we open the door for more precise and informative studies on evolution and adaptation not only in *A. bruennichi* but also in arachnids overall, shedding light on questions such as the genomic architecture of traits, whole-genome duplication, and the genomic mechanisms behind silk and venom evolution.

## Data description

### Context

Spider genomes are of great interest, e.g., in the context of silk and venom evolution and biomedical and technical applications. In addition, spiders are fascinating from ecological and evolutionary perspectives. As the most important predators of terrestrial arthropods, they play a key role in terrestrial food webs [1–4]. Spiders are distributed on every continent except Antarctica, and diverse habitats can be occupied by single species or multiple close relatives [5, 6], making them ideal for studies on environmental plasticity, adaptation, and speciation. With regards to adaptation, work on cobweb spiders (Theridiidae) has revealed a whole-genome duplication (WGD) that may have facilitated diversification [7], with other studies highlighting a key role of tandem duplication and neofunctionalization of genes in the diversification and specialization of spider silks [8] and venoms [9]. A key aspect that has been missing from studies to date is the role of genome organization in promoting or impeding adaptation because there have been no studies on spiders that have provided a chromosomal framework for the genome.

Understanding the chromosomal organization of a genome is critical for identification of processes underlying divergence between populations, adaptation, and speciation. Indeed, the potential role of chromosomal reorganization in species formation has long been the subject of debate, in particular in *Drosophila* species, where polytene chromosomes allowed early visualization of chromosomal rearrangements [10]. For spiders, karyotype data are still used to identify changes in chromosomes associated with speciation [11]. With the advent of detailed genomic data, there has been renewed focus on the role that structural variants in the genome can play as drivers of adaptation and speciation, associated with translocations, fusions, and inversions [12], as well as with admixture and associated demographic changes [13]. Recent data from sister species of the genus *Drosophila* suggest that the establishment of inversion polymorphisms within isolated and/or heterogeneous environments may well set the stage for species formation [14]. To develop a broader understanding of the role of structural variation in adaptation and speciation [15–22], we need chromosome-level genomes that provide the ability to map the order of genes, define chromosomal gene neighborhoods, and identify potential genomic islands of differentiation [23–26].

To the best of our knowledge, 10 draft spider genomes have been published to date [7, 27–33], most of which focus on silk and venom genes, while one discusses WGD [7], and the publication of the most recent two focuses on gene content evolution across arthropods [33]. There is one additional, as yet unpublished, spider genome assembly available on NCBI (*Anelosimus studiosus*, accession No. GCA_008297655.1). Spider genomes are considered notoriously difficult to sequence, assemble, and annotate for a number of reasons, including their relatively high repeat content, low guanine cytosine (GC) content, high levels of heterozygosity in the wild [27], and owing to the fact that they possess some extremely long coding genes in the spidroin gene families [28, 29, 34, 35]. As a result of these challenges, the completeness of the available spider genomes varies greatly between assemblies (Supplementary Table S1). All of them are incomplete and there is no chromosome-level assembly published for any spider to date. While this does not diminish the conclusions of the aforementioned studies, a chromosome-level assembly would open doors for more detailed studies on the genomic architecture of gene families, such as silk and venom genes, providing greater understanding of the evolutionary mechanisms driving the diversification of these gene families and genome evolution, in addition to the aforementioned applications in understanding adaptation and speciation.

**Figure 1:**
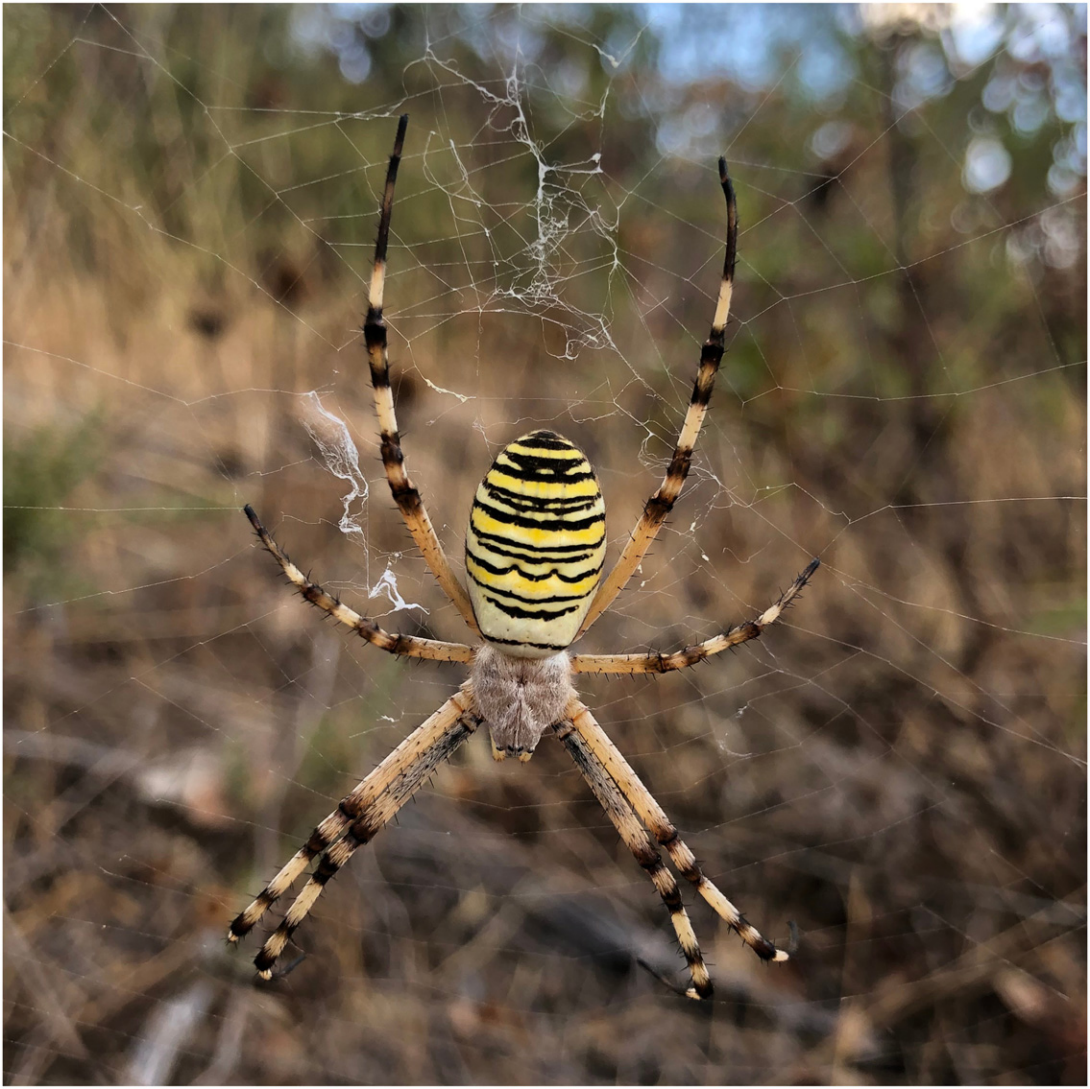
Female *Argiope bruennichi* spider in orb web from Loulé (Faro, Portugal). Photograph by Monica M. Sheffer.

The European wasp spider, *Argiope bruennichi* (Scopoli, 1772), is an orb-weaving spider in the family Araneidae (Fig. 1). Despite the lack of a reference genome, *A. bruennichi* has been the focal species for studies on local adaptation, range expansion, admixture, and biogeography [5, 36–38]. These studies have suggested that the range expansion and subsequent local adaptation of *A. bruennichi* from southern to northern Europe was caused by genetic admixture. However, it is not yet known which regions of the genome are admixed and whether these regions are truly responsible for adaptation to colder climates. *A. bruennichi* has also been well studied in the context of dispersal and life history traits [39], as well as sexual selection and chemical communication (e.g., [40–44]). A high-quality reference genome would allow new insights into our understanding of the genetic basis of these phenomena. Considering this background, a chromosome-level reference genome would be desirable for the species.

### Sampling, DNA extraction, and sequencing

Adult female *Argiope bruennichi* individuals (NCBI:txid94029) were collected in the south of Portugal in 2013 and 2019 (37° 44.34' N, 7° 51.18' W). Because inbred lines of the species do not exist, we selected a population that was previously found to have low heterozygosity in the wild, likely due to naturally high levels of inbreeding [5].

For the baseline assembly, DNA was extracted from a female collected in 2013 using the ArchivePure blood and tissue kit (5 PRIME, Hamburg, Germany), according to the manufacturer's protocol. An RNA digestion step was included using RNAse A solution (7,000 U mL^−1^; 5 PRIME). The DNA was stored at −80°C until library preparation in 2017. The DNA extract was cleaned using a salt: phenol chloroform isoamyl alcohol cleaning step and had a fragment size distribution of 1,300–165,500 bp (peak at 14,002 bp) before size selection. The library was size selected to 15 kb using Pippin prep and subsequently sequenced in 2018 at the QB3 Genomics facility at the University of California Berkeley on a Pacific Biosciences Sequel I platform (PacBio, Menlo Park, CA, USA) on 10 cells.

The specimen collected in 2019 was used to build a proximity-ligation-based short-read library (Hi-C). Four Hi-C libraries were prepared from a single individual using a Dovetail^TM^ Hi-C library preparation kit according to the manufacturer's protocol (Dovetail Genomics, Santa Cruz, CA). The specimen was anesthetized with CO_2_ before preparation. In brief, the legs were removed from the body and stored in liquid nitrogen, and the leg tissue was disrupted in liquid nitrogen using a mortar and pestle. Chromatin was fixed with formaldehyde, then extracted. Fixed chromatin was digested with DpnII, the 5′ overhangs filled in with biotinylated nucleotides, and the free blunt ends were ligated. After ligation, cross-links were reversed and the DNA was purified to remove proteins. Purified DNA was treated to remove biotin that was not internal to ligated fragments. The DNA was then sheared to ∼350 bp mean fragment size using a Covaris S2 Focused-ultrasonicator. A typical Illumina library preparation protocol followed, with end repair and Illumina adapter ligation. Biotinylated fragments were captured with streptavidin beads before PCR amplification (12 cycles), and size selection was performed using SPRI-select beads (Beckman Coulter GmbH, Germany) for a final library size distribution centered at ∼450 bp. The library was sequenced to ∼440 million paired-end reads on 1 Flowcell of an Illumina NextSeq 550 with a High Output v2 kit (150 cycles).

### Genome size estimation and coverage

We estimated the genome size of *Argiope bruennichi* on the basis of data for closely related species, and bioinformatically on the basis of previously published Illumina paired-end data derived from a single female individual from a population in Madeira (SRA accession No. ERX533198) [5], which we later used for polishing the assembly.

The closely related species *Argiope aurantia* and *Argiope trifasciata* have genome size estimates based on Feulgen densitometry data of 1.620 Gb [45] or 1.650 Gb [46] for *A. aurantia* and 1.690 Gb for *A. trifasciata* [45, 47]. Using the backmap.pl (v. 0.3) pipeline [48–55] on the Illumina data from *A. bruennichi* [5], we generated a genome size estimate of 1.740 Gb. Averaging these 4 genome size measurements yields an estimate of 1.675 Gb.

Given this estimate, the PacBio sequencing yielded 21.8× coverage (∼36.65 Gb sequenced, with an estimated genome size of 1.67 Gb). The previously published Illumina data [5] have a coverage of 19.8× (33.05 Gb sequenced).

### *De novo* genome assembly

**Figure 2:**
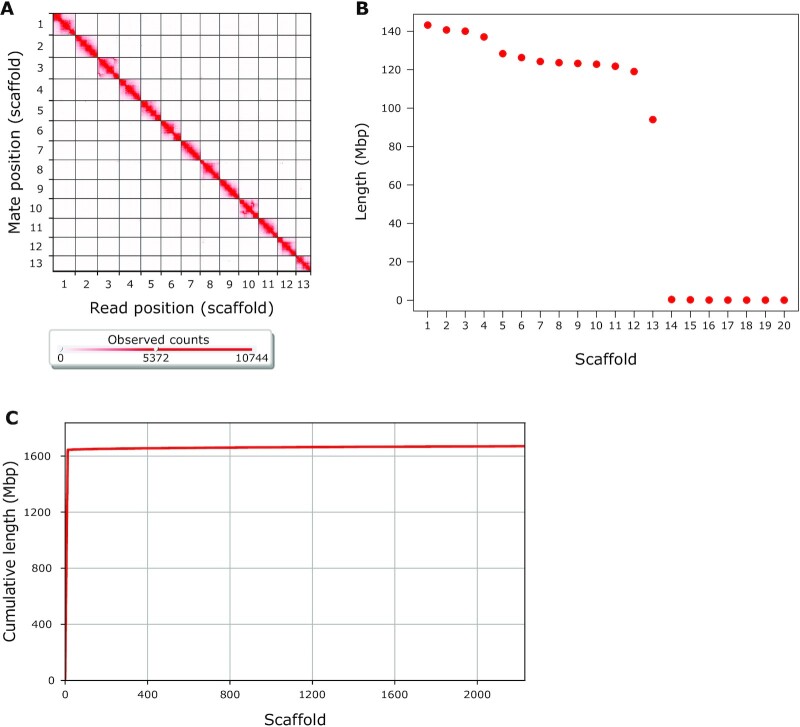
*Argiope bruennichi* genome assembly completeness. (A) Contact heat map of Hi-C scaffolding shows long-range contacts of paired-end Hi-C reads. Gray gridlines denote scaffold (chromosome) boundaries. Visualized with Juicebox (v. 1.11.08) [64]. (B) The length of the 20 longest scaffolds in the assembly shows that the 13 putative chromosome scaffolds are much larger than the next largest. Red points represent individual scaffolds, ordered from largest to smallest. (C) Cumulative length of assembly contained within scaffolds. Note that almost all (98.4%) of the genome is contained within very few scaffolds. Visualized with QUAST v. 5.0.2 [60] using default parameters, except –min-contig 0.

First, we generated a baseline assembly using 21.8× long-read PacBio Sequel I sequencing data and the wtdbg2 assembler (v. 2.3) (WTDBG, RRID:SCR_017225) [56]. Next, we polished the assembly by applying 3 rounds of Pilon (v. 1.23) (Pilon, RRID:SCR_014731) [57] using the 19.8× of previously published Illumina paired-end data [5]. Mapping for the 3 rounds of polishing resulted in a mapping rate ranging from 92.55% to 93.69%. The polishing resulted in 13,843 contigs with an N50 of 288.4 kb, and an overall assembly size of 1.67 Gb. Analysis of BUSCO (v. 3.1.0) scores, using the arthropod dataset (BUSCO, RRID:SCR_015008) [58], showed the presence of 90.2% of complete BUSCOs, with 86.4% complete and single-copy BUSCOs, 3.8% complete and duplicated BUSCOs, 3.3% fragmented BUSCOs, and 6.5% missing BUSCOs (Table 1). Next, we scaffolded the contigs using a proximity-ligation-based short-read library [59]. The sequences from this library had a 94.71% mapping rate against the polished assembly. Scaffolding using HiRise v. 2.1.7, a software pipeline designed specifically for using proximity ligation data to scaffold genome assemblies [59], resulted in 12 scaffolds >1 Mb in size and 1 scaffold just slightly less than 1 Mb in size. These 13 scaffolds comprise 98.4% of the assembly, with a genome assembly scaffold N50 of 124 Mb and BUSCO scores of 91.1% complete genes (Fig. 2, Table 1). Genome assembly statistics were calculated using QUAST v. 5.0.2 (QUAST, RRID:SCR_001228) [60] applying default parameters, except setting the minimum contig length (–min-contig parameter) to 0. Previous studies have inferred the chromosome number of *A. bruennichi* to be 13, indicating that our genome assembly achieved full-chromosome level [61, 62]. As an additional assessment of assembly quality, we ran the K-mer Analysis Toolkit v. 2.4.2 (KAT, RRID:SCR_016741) [63] “comp" tool, comparing the *k*-mer content in the Illumina sequencing data to the *k*-mer content in the final assembly. Different values of the parameter *k* (*k* = 17, 27, 29, 30, and 37) yielded *k*-mer completeness estimates ranging from 86.55% to 90.43% (Supplementary Fig. S1). The missing *k*-mer content in the final assembly may be attributed to errors remaining in the assembly, likely within repeat regions. This could be attributed to the moderate 19.8× coverage Illumina reads used for polishing and their short read length, which may have been insufficient to correct the more error-prone PacBio reads.

**Table 1:** *Argiope bruennichi* genome assembly completeness

Genome assembly statistic	Unscaffolded	Scaffolded
Assembly size (bp)	1,669,116,561	1,670,285,661
AT/GC/N content (%)	70.7/29.3/0	70.6/29.3/0.1
No. of contigs/scaffolds	13,843	2,231
Longest contig/scaffold (bp)	2,039,454	143,171,375
Contig/scaffold N50 (bp)	288,395	124,235,998
Contig/scaffold N90 (bp)	67,231	119,022,586
% Repetitive	34.66	34.64
BUSCO analysis**^a^**		
Complete (%)	90.2	91.1
Complete and single-copy (%)	86.4	87.8
Complete and duplicated (%)	3.8	3.3
Fragmented (%)	3.3	2.8
Missing (%)	6.5	6.1

Genome assembly statistics were calculated using QUAST v. 5.0.2 (QUAST, RRID:SCR_001228) [60] using default parameters, except –min-contig 0. AT: adenine thymine.

a BUSCO analysis using default parameters against the arthropod dataset.

The 13 largest scaffolds are henceforth referred to as Chromosomes 1–13, ordered according to size (Fig. 2B). The 14th-largest scaffold (Scaffold 839) contained the 16S sequence of a recently discovered, as yet unnamed, bacterial symbiont of *A. bruennichi* [48]. The remaining 2,217 scaffolds are much smaller, ranging from 1,747 to 258,743 bp in length (Supplementary Fig. S2) and will henceforth be referred to as “lesser scaffolds.”

### Repeat masking and removal of contaminants

The assembly was repeat-masked using a combination of the *de novo* repeat finder RepeatModeler (v. open-1.0.11) (RepeatModeler, RRID:SCR_015027) [65] and the homology-based repeat finder RepeatMasker (v. open-4.0.9) (RepeatMasker, RRID:SCR_012954) [66]. Repetitive regions accounted for 34.64% of the genome assembly, of which the majority (20.52% of the genome) consisted of unclassified repeats, meaning that they have not been classified in previous studies. The remaining repetitive elements were made up of DNA elements (i.e., transposable elements: 6.27%), long interspersed nuclear elements (LINEs: 1.60%), simple repeats (i.e., duplications of 1–5 bp: 1.58%), long terminal repeat (LTR) elements (0.76%), satellites (0.63%), low-complexity repeats (i.e., polypurine or polypyrimidine stretches: 0.42%), and short interspersed nuclear elements (SINEs: 0.08%) (Table 2). BlobTools (v. 1.0) (Blobtools, RRID:SCR_017618) [67] was used to search for contamination by bacterial or mitochondrial sequences, finding none.

**Table 2:** *Argiope bruennichi* repetitive DNA elements

Type of element	No. of elements	Length (bp)	Proportion of assembly (%)
SINEs	4,643	1,314,740	0.08
LINEs	52,648	26,768,096	1.60
LTR elements	21,649	12,683,330	0.76
DNA elements	282,019	104,785,665	6.27
Unclassified	1,359,138	342,727,030	20.52
Small RNA	0	0	0
Satellites	28,474	10,495,658	0.63
Simple repeats	595,962	26,379,486	1.58
Low complexity	137,182	6,952,634	0.42
**Total**			34.64

Repetitive elements were classified using RepeatModeler (v. open-1.0.11) [65] and RepeatMasker (v. open-4.0.9) [66].

### Genome annotation

Raw reads from previously published transcriptome sequencing data of different life stages: 20 pooled eggs (accession No. SRR11861505), 20 pooled first instar spiderlings (accession No. SRR11861504), 1 whole body of an adult female (accession No. SRR11861502), and 1 whole body of an adult male (accession No. SRR11861503) [5] were mapped against the repeat-masked assembly using HISAT2 (v. 2.1.0) (HISAT2, RRID:SCR_015530) [68]. After conversion of the resulting SAM file into a BAM file and subsequent sorting using SAMtools (v. 1.7) (SAMTOOLS, RRID:SCR_002105) [49], the sorted BAM file was converted to intron-hints for AUGUSTUS (v. 3.3.2) (Augustus, RRID:SCR_008417) [69] using AUGUSTUS scripts. AUGUSTUS was run on the soft-masked genome with the *Parasteatoda* parameter set. The resulting gff file containing predicted genes was converted into a gtf file using the AUGUSTUS script gtf2gff.pl. Additional AUGUSTUS scripts (getAnnoFastaFromJoinGenes.py and fix_in_frame_stop_codon_genes.py) were used to find and replace predicted protein-coding genes containing in-frame stop codons with newly predicted genes. The resulting gtf file containing 23,270 predicted genes (26,318 transcripts) was converted to gff3 format using gtf2gff.pl and protein sequences of predicted genes were extracted with getAnnoFastaFromJoinGenes.py. Finally, functional annotation was performed using InterProScan (v. 5.39–77.0) (InterProScan, RRID:SCR_005829) [70, 71] (Table 3). The majority of annotated genes fall on the 13 chromosome scaffolds, although 272 transcripts were predicted on the lesser scaffolds. The annotation gff3 file and the files containing predicted transcripts and proteins are available on GigaDB [72].

**Table 3:** *Argiope bruennichi* genome annotation statistics

Statistic	Value
No. of protein-coding genes	23,270
Functionally annotated genes (%)	81.0
Mean exon length (bp)	200
Mean intron length (bp)	4,035
BUSCO analysis**^a^**	
Complete (%)	89.3
Complete and single-copy (%)	76.7
Complete and duplicated (%)	12.6
Fragmented (%)	7.0
Missing (%)	3.7

a BUSCO analysis using default parameters against the arthropod dataset.

### Comparative genomic analysis of repeat content

High repetitiveness is characteristic of spider genomes [27]. To compare the repeat content of *A. bruennichi* with that of other spiders, we downloaded the genome assemblies of several other spider species from NCBI and DDBJ (accession numbers in Table 4), then treated them in the same manner as the *A. bruennichi* genome, masking the repeats using RepeatModeler (v. open-1.0.11) [65] and RepeatMasker (v. open-4.0.9) [66]. *Acanthoscurria geniculata* was excluded from this analysis owing to the relatively poorly assembled genome. The *A. bruennichi* genome has a slightly lower percentage of repetitive element content (34.64%) compared to most other spiders (Table 4). Some species, such as *Loxosceles reclusa, Trichonephila clavipes* (formerly *Nephila clavipes*), *Anelosimus studiosus*, and *Parasteatoda tepidariorum*, have similar repetitive content (36.51%, 36.61%, 35.98%, and 36.79%, respectively); other species have much higher repetitive content, such as *Araneus ventricosus, Dysdera silvatica, Stegodyphus dumicola, Stegodyphus mimosarum*, and *Pardosa pseudoannulata* (55.96%, 60.03%, 58.98%, 56.91%, and 48.61%, respectively). Only *Latrodectus hesperus* has lower repetitive content (20.97%). The classification and relative percentage of these repeats can be found in Supplementary Table S2 and Supplementary Fig. S3. It is often asserted that the repeat content in spiders is higher in general than in other arthropod groups (i.e., [27]). To test this assertion, we looked into the repeat content in genomes of additional arthropod species. We obtained repeat content estimates, for which the repeats were masked using RepeatModeler and RepeatMasker, for 3 insect species (*Bombus terrestris, Drosophila melanogaster*, and *Rhodnius prolixus* [73]) and 7 tick and mite species (*Ixodes persulcatus, Haemaphysalis longicornis, Dermacentor silvarum, Hyalomma asiaticum, Rhipicephalus sanguineus,Rhipicephalus microplus*, and*Ixodes scapularus* [74]). We additionally downloaded the genomes of 4 more arthropod species, generated custom species-specific repeat libraries with RepeatModeler, and masked the genomes with RepeatMasker to avoid any issues of under- or overmasking using other repeat-masking programs: a butterfly, *Heliconius melpomene* [75]; a beetle, *Tribolium castaneum* [76]; a millipede, *Helicorthomorpha holstii* [77]; and a scorpion, *Centruroides sculpturatus* [7, 33]. The percentage of total repetitive content for all of these species is presented in Table 4. In general, spiders do have a higher repetitive content than insects, but there is a large range of repetitive content in spiders, compared to which the repetitive content in *A. bruennichi* is relatively low. All of the selected spider species, aside from *L. hesperus*, have higher repetitive content than all other investigated groups, with the exception of ticks and mites, which have very high repetitive content overall (range: 52.6–64.4% repetitive). We conclude from this preliminary investigation that spider genomes, and arachnid genomes generally, do indeed have a higher repeat content than other arthropods.

**Table 4:** Total repetitive content in the genomes of spiders and selected other arthropods

Class	Order	Species	% Repetitive	Accession No. [reference]
Arachnida	Araneae	*Argiope bruennichi*	34.64	
		*Araneus ventricosus*	55.96	BGPR01000001-BGPR01300721^a^ [29]
		*Trichonephila clavipes*	36.61	GCA_002102615.1^b^ [28]
		*Dysdera silvatica*	60.03	GCA_006491805.1^b^ [32]
		*Stegodyphus dumicola*	58.98	GCA_010614865.1^b^ [31]
		*Stegodyphus mimosarum*	56.91	GCA_000611955.2^b^ [27]
		*Pardosa pseudoannulata*	48.61	GCA_008065355.1^b^ [30]
		*Loxosceles reclusa*	36.51	GCA_001188405.1^b^ [33]
		*Anelosimus studiosus*	35.98	GCA_008297655.1^b^,^c^
		*Latrodectus hesperus*	20.97	GCA_000697925.2^b^ [33]
		*Parasteatoda tepidariorum*	36.79	GCA_000365465.3^b^ [7]
	Scorpiones	*Centruroides sculpturatus*	34.40	GCA_000671375.2^b^ [7, 33]
	Acari	*Ixodes persulcatus*	64.40	GCA_013358835.1^b^ [74]
		*Haemaphysalis longicornis*	59.30	GCA_013339765.1^b^ [74]
		*Dermacentor silvarum*	60.20	GCA_013339745.1^b^ [74]
		*Hyalomma asiaticum*	52.60	GCA_01333685.1^b^ [74]
		*Rhipicephalus sanguineus*	61.60	GCA_013339695.1^b^ [74]
		*Rhipicephalus microplus*	63.10	GCA_013339725.1^b^ [74]
		*Ixodes scapularis*	63.50	GCF_002892825.2^b^ [74, 78]
Diplopoda	Helminthomorpha	*Helicorthomorpha holstii*	23.50	GCA_013389785.1^b^ [77]
Insecta	Hemiptera	*Rhodnius prolixus*	29.25	GCA_000181055.3^b^ [73]
	Hymenoptera	*Bombus terrestris*	12.51	GCA_000214255.1^b^ [73]
	Coleoptera	*Tribolium castaneum*	28.50	GCA_000002335.3^b^ [76]
	Lepidoptera	*Heliconius melpomene*	32.40	GCA_000313835.2^b^ [75]
	Diptera	*Drosophila melanogaster*	19.31	GCA_000001215.4^b^ [73]

Repetitive elements were classified using RepeatModeler (v. open-1.0.11) [65] and RepeatMasker (v. open-4.0.9) [66].

a DNA Data Bank of Japan (DDBJ).

b GenBank, NCBI.

c [unpublished, Jessica Purcell].

### Genome architecture of Hox, spidroin, and venom genes

Previous studies on spider genomes have focused on WGD, silk gene evolution, and venom gene evolution [7, 27–30]. Therefore, to place the *A. bruennichi* genome into the same context, we manually curated 3 gene sets from publicly available protein sequences: Hox, spidroin (silk), and venom genes. Because Hox genes are highly conserved across taxa [79], we chose the most complete sequences for the 10 arthropod Hox gene classes from spiders without regard to the relatedness of the species to *A. bruennichi* (Supplementary File S1). In contrast to Hox genes, spidroin and venom genes are highly polymorphic and species specific [80–83]. For the spidroin gene set, we downloaded protein sequences of the 7 spidroin gene classes exclusively from 5 species of the genus *Argiope* (Supplementary File S2). Venom genes are best studied in spiders that are medically significant to humans, which are very distant relatives to *A. bruennichi* [84–87]. To allow comparison, we focused on venom gene sequences available for araneid spiders (2 species, Supplementary File S3); however, the function and classification of these genes is poorly understood. With these 3 gene sets (Hox, spidroin, and venom), we performed a TBLASTN search against our genome assembly (v. 2.10.0+) (TBLASTN, RRID:SCR_011822) [88, 89]. We recorded the genomic position of the best matches and compared them with the AUGUSTUS gene predictions for those locations. We used a conservative E-value cut-off of <1.00 × 10^−20^ and only included results with an identity >60%. If hits overlapped on a scaffold or mapped to the same gene, only the hit with the highest identity and lowest E-value was retained. In cases where these metrics conflicted, the hit with the longest match length was retained. The manually curated FASTA files of each gene set used for the TBLASTN search are available in Supplementary Files S1–S3 and on GigaDB [72]. A table of the best matches with accession numbers for each gene set is available in Supplementary Tables S3–S5.

### Hox cluster duplication

**Figure 3:**
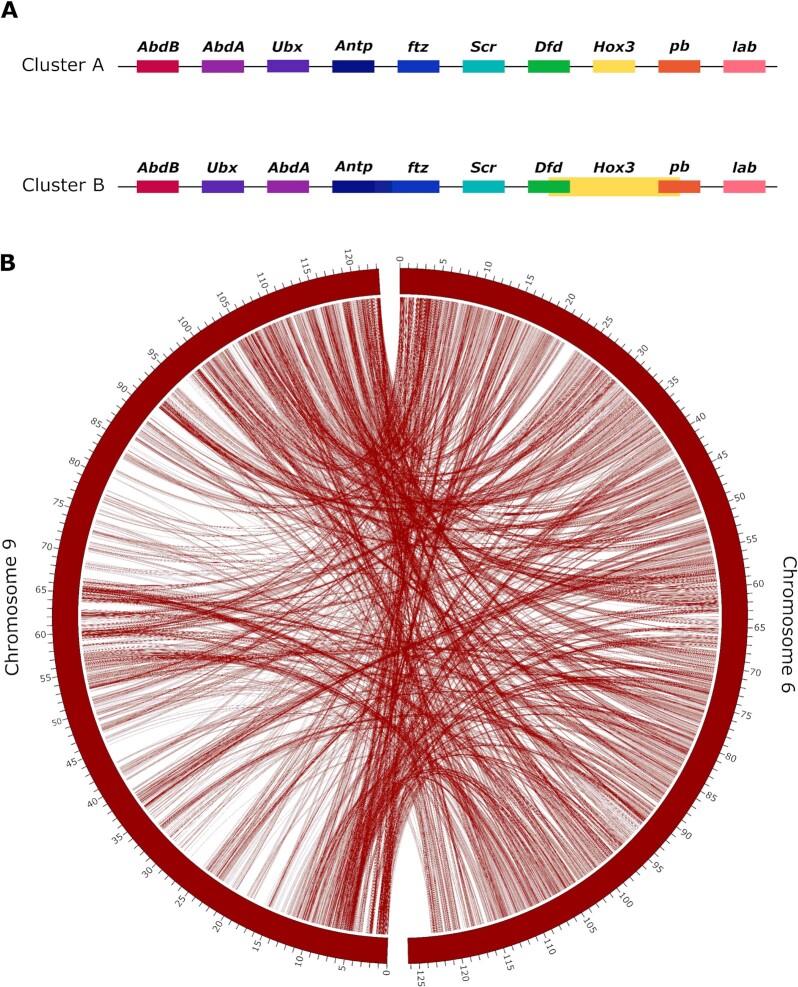
Duplication of the Hox-containing chromosomes. (A) Hox gene clusters. Genes connected by a black line occur on the same scaffold, in the order depicted. Cluster A occurs on Chromosome 9, and Cluster B occurs on Chromosome 6. (B) A synteny plot of the results of SatsumaSynteny2 [90] visualized in Circos [92] shows chromosome-scale conservation of synteny for the Hox-containing chromosomes (Chromosomes 6 and 9). The 2 curved rectangles represent Chromosomes 6 and 9, and the tick marks represent the position on the chromosome, in megabase pairs. Lines between the 2 rectangles show the shared syntenic blocks between the chromosomes, based on sequence homology. The presence of 2 Hox gene clusters on 2 highly syntenic chromosomes is suggestive of whole-genome duplication in *Argiope bruennichi*, as was found previously for *Parasteatoda tepidariorum* [7].

In 2017, Schwager et al. revealed that a WGD event occurred in the ancestor of scorpions and spiders, as evidenced by a high number of duplicated genes, including 2 clusters of Hox genes in the common house spider *P. tepidariorum* and the bark scorpion *C. sculpturatus* [7]. They found 1 nearly complete cluster of Hox genes on a single scaffold, lacking the *fushi tarazu (ftz)* gene, which they argued may be the case for this cluster in all spiders. The second set of Hox genes was distributed across 2 scaffolds, which the authors attributed to incompleteness of the assembly due to patchy sequencing coverage [7]. For consistency, we use the same nomenclature for Hox genes as used in [7] (*Abdominal-B: AbdB, Abdominal-A: AbdA, Ultrabithorax: Ubx, Antennapedia: Antp, fushi tarazu: ftz, sex combs reduced: scr, Deformed: Dfd, Hox3, proboscipedia: pb, labial: lab*). Corresponding with the results from *P. tepidariorum*, we found 2 clusters of Hox genes in *A. bruennichi*, with no evidence of tandem duplication. The 2 clusters occurred on 2 chromosomes (Chromosomes 6 and 9). In these locations, InterProScan generally annotated the genes as Hox genes but did not identify the specific type. On Chromosome 9, the Hox genes were in reverse colinear order (ordered according to their expression in development), with no overlapping regions. Because the cluster on Chromosome 9 is complete, we refer to it as “Cluster A.” On Chromosome 6 (“Cluster B”) the genes were out of colinear order, with the position of *AbdA* and *Ubx* switched, and the coordinates for *Dfd, Hox3*, and *pb* from the blast search overlapping (Fig. 3A). The hits for *Antp* and *ftz* in Cluster B fell onto a single predicted gene in the annotation. Thus, it is unclear whether *A. bruennichi* lacks 1 copy of *ftz*, as in *P. tepidariorum*, or whether the annotation incorrectly fused the 2 genes in this cluster. In the study by Schwager et al. [7], low sequencing coverage of Cluster B downstream of *Dfd* limited their inference. In our genome assembly, by mapping the PacBio reads against the final assembly, we calculated that we have an average of >12× coverage across the length of both clusters, suggesting that Cluster B is not out of order due to problems arising from low coverage. It is possible that Hox Cluster B in spiders has changed or lost functionality following the proposed ancestral WGD event. To check whether the 2 Hox-containing chromosomes show evidence of duplication, we performed an analysis of conserved synteny using the tool SatsumaSynteny2 [90]. “Synteny” here refers to loci occurring on the same chromosome; chromosomes with conserved synteny will have a high degree of syntenic blocks in common. In the genome of *A. bruennichi*, Chromosomes 6 and 9 show a high level of conserved synteny (Fig. 3B). The presence of 2 Hox clusters on highly syntenic chromosomes in our assembly is suggestive, but not evidence, of WGD in *A. bruennichi*because it could also have arisen from duplication of only the ancestral Hox-containing chromosome; future studies will be able to capitalize on the now-available chromosome-level assemblies for several groups (e.g., horseshoe crabs, ticks, and our spider) [74, 91] to perform more detailed analyses of duplication across chelicerates.

### Spidroin genes

**Figure 4:**
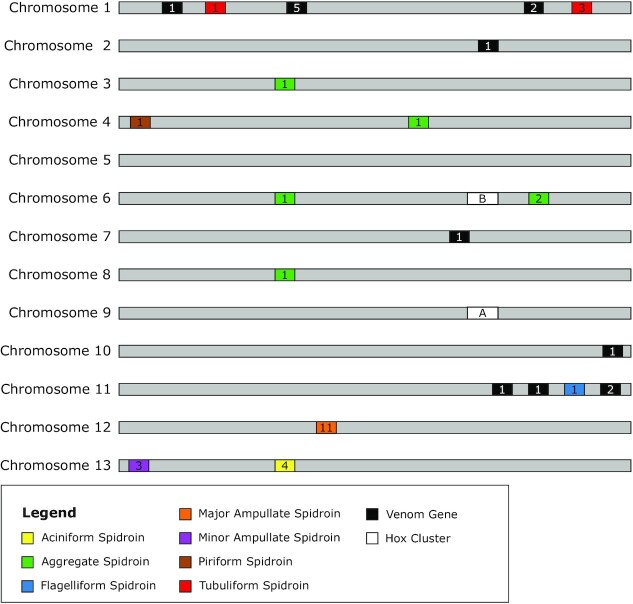
Schematic representation of the location of gene families on the 13 chromosomes. The light grey bars represent chromosomes, the coloured rectangles represent the 7 different spidroin gene families, the black rectangles represent venom genes, and the white rectangles represent Hox gene clusters. The numbers inside of the rectangles represent the number of genes found within that cluster.

There are 7 classes of silk produced by araneomorph spiders, each with 1 or more unique uses; it is important to note that the uses of these silk types are best understood for spiders in the family Araneidae, and the number and uses of silk types can vary widely between families [28, 29, 93, 94]. The classes of silk are major ampullate (*MaSp*), minor ampullate (*MiSp*), piriform (*PiSp*), aggregate (*AgSp*), aciniform (*AcSp*), tubuliform (also referred to as cylindrical) (*TuSp*), and flagelliform (*Flag*). In *A. bruennichi*, spidroin genes occur on 8 of the 13 chromosome scaffolds (Chromosomes 1, 3, 4, 6, 8, 11, 12, and 13) (Fig. 4). There were no hits on the lesser scaffolds. We found 4 unique hits for *AcSp*, 6 hits for *AgSp*, 1 hit for *Flag*, 11 hits for *MaSp*, 3 hits for *MiSp*, 1 hit for *PiSp*, and 4 hits for *TuSp*. In the majority of cases, all blast hits for a single spidroin type occurred on a single chromosome; the only exception was for *AgSp*, which had hits on 4 different chromosomes. However, these were not all annotated as spidroins; on Chromosome 6 there were 2 *AgSp* hits that were annotated as spidroins and 1 hit that was annotated as a chitin-binding domain, while on Chromosome 4 the *AgSp* hit was annotated as tropoelastin, on Chromosome 3 the hit was annotated as a chitin-binding domain, and on Chromosome 8 the hit was annotated as a serine protease. All hits for *TuSp* occurred on Chromosome 1, but there were hits in 2 physically separated areas of the chromosome; in 1 region there were hits on 3 annotated genes, and only 1 hit in the other region. There are more sequences available on NCBI for *MaSp* than any of the other spidroin types in the genus *Argiope*, which allowed us to find matches for several unique *MaSp* genes in the *A. bruennichi* assembly. These occur in a small region of Chromosome 12, in close proximity to one another, suggesting that the spidroin genes in this group may have diversified via tandem duplication, as has been suggested in previous studies [95].

### Venom genes

We found high identity matches for venom toxins on 5 of the chromosome scaffolds (Chromosomes 1, 2, 7, 10, and 11) (Fig. 4), but the majority of hits were on Chromosome 1. In most cases, each region containing venom gene matches contained only 1 gene, with the exception of a region on Chromosome 1, which contained 5 genes in very close proximity to one another, and 2 other regions (on Chromosome 1 and Chromosome 11), which contained matches to 2 genes. Babb et al. 2017 [28] conducted a study on silk genes in *T. clavipes*, in which they found a novel flagelliform-type gene (*FLAG-b*), which was expressed most highly in the venom glands, not the flagelliform silk glands. This added to previous findings in the *S. mimosarum* genome, where spidroin-like proteins are also found in the venom glands [27]. Interestingly, in the *A. bruennichi* genome assembly, there are several venom genes on Chromosome 11 in close proximity to the flagelliform spidroin gene.

## Conclusions

We have assembled and annotated the first chromosome-level genome for a spider. The assembly approach of combining long-read, short-read, and proximity ligation data overcame the challenges of assembling arachnid genomes, namely, large genome size, high repetitiveness, and low GC content. In our study, we made a preliminary analysis of the location of certain gene families of interest in the context of spider genomics, which hinted at several interesting directions for future studies on the evolution of silk and venom genes. Furthermore, because this species has undergone a recent and rapid range expansion, the well-resolved genome assembly will be useful for studies on the genomic underpinnings of range expansion and evolutionary adaptation to novel climates.

## Data Availability

The final genome assembly and raw data from the PacBio and Hi-C libraries, as well as the annotation, have been deposited at NCBI under BioProject PRJNA629526. A publicly accessible genome browser hub with the annotation, raw transcriptome, and PacBio read coverage can be found on the UCSC Genome Browser server (under “My Data” > “Track Hubs” > “My Hubs” enter the cited URL [96]). Supporting data are available via the *GigaScience* data repository, GigaDB, including the softmasked assembly in FASTA format, the output file from RepeatMasker, predicted coding genes and their functional annotation in GFF3 formats, predicted coding gene nucleotide and translated sequences in FASTA formats, functional annotation from InterProScan in TSV format, the blast query results for Hox, spidroin, and venom genes in FASTA format, and the BUSCO output files in a zip folder [72].

## Additional Files

Supplementary Figure S1. KAT plots

Supplementary Figure S2. Histogram of minor scaffold lengths

Supplementary Figure S3. Stacked barplot of repeat content in spiders

Supplementary File S1. Hox blast query sequences

Supplementary File S2. Spidroin blast query sequences

Supplementary File S3. Venom blast query sequences

Supplementary Table S1. Spider genome assembly statistics

Supplementary Table S2. Repetitive content in spiders

Supplementary Table S3. Hox blast results

Supplementary Table S4. Spidroin blast results

Supplementary Table S5. Venom blast results

## Abbreviations

*Abd-A: Abdominal-A; Abd-B: Abdominal-B; AcSp:* aciniform spidroin;*AgSp:* aggregate spidroin;*Antp: Antennapedia;* AT: adenine thymine; bp: base pairs; BUSCO: Benchmarking Universal Single Copy Orthologs; DDBJ: DNA Data Bank of Japan;*Dfd: Deformed; Flag:* flagelliform spidroin;*ftz: fushi tarazu;* Gb: gigabase pairs; GC: guanine cytosine; kb: kilobase pairs;*lab: labial;* LINE: long interspersed nuclear element; LTR: long terminal repeat;*MaSp:* major ampullate spidroin; Mb: megabase pairs;*MiSp:* minor ampullate spidroin; NCBI: National Center for Biotechnology Information; PacBio: Pacific Biosciences;*pb: proboscipedia; PiSp:* piriform spidroin; *scr: sex combs reduced;* SINE: short interspersed nuclear element; SRA: Sequence Read Archive; TSV: tab-separated value;*TuSp:* tubuliform spidroin;*Ubx: Ultrabithorax;* UCSC: University of California Santa Cruz; WGD: whole-genome duplication.

## Competing Interests

The authors declare that they have no competing interests.

## Funding

Funding for this study was provided by the Deutsche Forschungsgemeinschaft (DFG) as part of the Research Training Group 2010 RESPONSE (GRK 2010) to G.U.

## Authors’ Contributions

M.M.S., H.K., G.U., and S.P. conceived of the study; M.M.S., H.K., and G.U. collected the spiders. H.K. extracted DNA for the PacBio sequencing; M.M.S. prepared and submitted the DNA for PacBio sequencing, with input and infrastructure provided by R.G.G. M.M.S. and C.J. constructed and sequenced the Hi-C library, with input and infrastructure provided by L.J. and A.W.K. M.M.S., A.H., and S.P. performed the genome assembly, and A.H. and K.J.H. performed the genome annotation with input and infrastructure provided by M.M.S. and S.P. A.H. and K.J.H. analysed the repeat content of other arthropod species; M.M.S. performed the analysis of Hox duplication, spidroin genes, and venom genes. M.M.S., A.H., K.J.H., and S.P. wrote the first draft of the manuscript. All authors read and approved the final manuscript.

## Supplementary Material

giaa148_GIGA-D-20-00146_Original_Submission

giaa148_GIGA-D-20-00146_Revision_1

giaa148_GIGA-D-20-00146_Revision_2

giaa148_Response_to_Reviewer_Comments_Original_Submission

giaa148_Response_to_Reviewer_Comments_Revision_1

giaa148_Reviewer_1_Report_Original_Submission Jessica Garb -- 7/7/2020 Reviewed

giaa148_Reviewer_1_Report_Revision_1 Jessica Garb -- 11/3/2020 Reviewed

giaa148_Reviewer_2_Report_Original_Submission Jean-FranÃ§ois Flot -- 8/23/2020 Reviewed

giaa148_Reviewer_2_Report_Revision_1 Jean-FranÃ§ois Flot -- 11/10/2020 Reviewed

giaa148_Supplemental_Files
