# Supplementary material for: Chromosome-level reference genome of the European wasp spider Argiope bruennichi: a resource for studies on range expansion and evolutionary adaptation
Source: Gigascience. 2021 Jan 7;10(1):giaa148. doi: 10.1093/gigascience/giaa148 (PMC7788392; doi:10.1093/gigascience/giaa148)
Supplement: giaa148_GIGA-D-20-00146_Original_Submission [file giaa148_giga-d-20-00146_original_submission.pdf]

GigaScience

Chromosome-level reference genome of the European wasp spider Argiope bruennichi: a resource for studies on range expansion and evolutionary adaptation

--Manuscript Draft--

**Manuscript Number:**	GIGA-D-20-00146
**Full Title:**	Chromosome-level reference genome of the European wasp spider Argiope bruennichi: a resource for studies on range expansion and evolutionary adaptation
**Article Type:**	Data Note
**Funding Information:**	Deutsche Forschungsgemeinschaft (GRK 2010) — Dr. Gabriele Uhl
**Abstract:**	Background<br><br>Argiope bruennichi , the European wasp spider, has been studied intensively as to sexual selection, chemical communication, and the dynamics of rapid range expansion at a behavioral and genetic level. However, the lack of a reference genome has limited insights into the genetic basis for these phenomena. Therefore, we assembled a high-quality chromosome-level reference genome of the European wasp spider as a tool for more in-depth future studies.<br><br>Findings<br><br>We generated, de novo , a 1.67Gb genome assembly of A. bruennichi using 21.5X PacBio sequencing, polished with 30X Illumina paired-end sequencing data, and proximity ligation (Hi-C) based scaffolding. This resulted in an N50 scaffold size of 124Mb and an N50 contig size of 288kb. We found 98.4% of the genome to be contained in 13 scaffolds, fitting the expected number of chromosomes (n = 13). Analyses showed the presence of 91.1% of complete arthropod BUSCOs, indicating a high quality of the assembly.<br><br>Conclusions<br><br>We present the first chromosome-level genome assembly in the class Arachnida. With this genomic resource, we open the door for more precise and informative studies on evolution and adaptation in A. bruennichi , as well as on several interesting topics in Arachnids, such as the genomic architecture of traits, whole genome duplication and the genomic mechanisms behind silk and venom evolution.
**Corresponding Author:**	Monica M Sheffer<br>Zoological Institute and Museum, University of Greifswald, Germany<br>Greifswald, GERMANY
**Corresponding Author Secondary Information:**	
**Corresponding Author's Institution:**	Zoological Institute and Museum, University of Greifswald, Germany
**Corresponding Author's Secondary Institution:**	
**First Author:**	Monica M Sheffer
**First Author Secondary Information:**	
**Order of Authors:**	Monica M Sheffer
	Anica Hoppe
	Henrik Krehenwinkel
	Gabriele Uhl
	Andreas W. Kuss
	Lars Jensen
	Corinna Jensen

Powered by Editorial Manager® and ProduXion Manager® from Aries Systems Corporation

	Rosemary G. Gillespie
	Katharina J. Hoff
	Stefan Prost
**Order of Authors Secondary Information:**	
**Additional Information:**	
**Question**	**Response**
Are you submitting this manuscript to a special series or article collection?	No
**Experimental design and statistics**<br><br>Full details of the experimental design and statistical methods used should be given in the Methods section, as detailed in our Minimum Standards Reporting Checklist. Information essential to interpreting the data presented should be made available in the figure legends.<br><br>Have you included all the information requested in your manuscript?	Yes
**Resources**<br><br>A description of all resources used, including antibodies, cell lines, animals and software tools, with enough information to allow them to be uniquely identified, should be included in the Methods section. Authors are strongly encouraged to cite Research Resource Identifiers (RRIDs) for antibodies, model organisms and tools, where possible.<br><br>Have you included the information requested as detailed in our Minimum Standards Reporting Checklist?	Yes
**Availability of data and materials**<br><br>All datasets and code on which the conclusions of the paper rely must be either included in your submission or deposited in publicly available repositories (where available and ethically	Yes

Powered by Editorial Manager® and ProduXion Manager® from Aries Systems Corporation

appropriate), referencing such data using a unique identifier in the references and in the “Availability of Data and Materials” section of your manuscript.<br><br>Have you have met the above requirement as detailed in our Minimum Standards Reporting Checklist?	

Powered by Editorial Manager® and ProduXion Manager® from Aries Systems Corporation

Manuscript

Click here to download Manuscript ArgiopeGenome-Manuscript-final.docx

Chromosome-level reference genome of the European wasp spider *Argiope bruennichi*: a resource for studies on range expansion and evolutionary adaptation

Monica M. Sheffer[1†], Anica Hoppe[2,3], Henrik Krehenwinkel[4], Gabriele Uhl[1], Andreas W. Kuss[5], Lars Jensen[5], Corinna Jensen[5], Rosemary G. Gillespie[6], Katharina J. Hoff[2,3]* & Stefan Prost[7,8]*

*indicates equal contribution

†indicates corresponding author

[1] Zoological Institute and Museum, University of Greifswald, Germany

[2] Institute of Mathematics and Computer Science, University of Greifswald, Germany

[3] Center for Functional Genomics of Microbes, University of Greifswald, Germany

[4] Department of Biogeography, University of Trier, Germany

[5] Interfaculty Institute for Genetics and Functional Genomics, University of Greifswald, Germany

[6] Department of Environmental Science Policy and Management, University of California Berkeley, USA

[7] LOEWE-Centre for Translational Biodiversity Genomics, Germany

[8] South African National Biodiversity Institute, National Zoological Gardens of South Africa, South Africa

Abstract

Background: *Argiope bruennichi*, the European wasp spider, has been studied intensively as to sexual selection, chemical communication, and the dynamics of rapid range expansion at a behavioral and genetic level. However, the lack of a reference genome has limited insights into the genetic basis for these phenomena. Therefore, we assembled a high-quality chromosome-level reference genome of the European wasp spider as a tool for more in-depth future studies.

Findings: We generated, *de novo*, a 1.67Gb genome assembly of *A. bruennichi* using 21.5X PacBio sequencing, polished with 30X Illumina paired-end sequencing data, and proximity ligation (Hi-C) based scaffolding. This resulted in an N50 scaffold size of 124Mb and an N50 contig size of 288kb. We found 98.4% of the genome to be contained in 13 scaffolds, fitting the expected number of chromosomes (n = 13). Analyses showed the presence of 91.1% of complete arthropod BUSCOs, indicating a high quality of the assembly.

Conclusions: We present the first chromosome-level genome assembly in the class Arachnida. With this genomic resource, we open the door for more precise and informative studies on evolution and adaptation in *A. bruennichi*, as well as on several interesting topics in Arachnids, such as the genomic architecture of traits, whole genome duplication and the genomic mechanisms behind silk and venom evolution.

Keywords

Argiope bruennichi, genome assembly, Araneae, spider, PacBio, Hi-C, chromosome-level, whole genome duplication, silk, venom

Data description

Context

Spider genomes are of great interest, for instance in the context of silk and venom evolution and biomedical and technical applications. Additionally, spiders are fascinating from ecological and evolutionary perspectives. As the most important predators of terrestrial arthropods, they play a key role in terrestrial food webs [1–4]. Spiders are distributed on every continent, except Antarctica, and diverse habitats can be occupied by single species or multiple close relatives [5,6], making them ideal for studies on environmental plasticity, adaptation and speciation. A chromosome-level genome assembly would greatly increase the potential for inference on evolutionary adaptation and modes of speciation [7]. For instance, a well-resolved genome is critical, if evolutionary adaptation happens along genomic islands of differentiation [8–11] or to assess the importance of large genomic rearrangements, such as inversions, in speciation [12–18].

To the best of our knowledge, only eight draft spider genomes have been published to date [19–25], most of which focus on silk and venom genes, while one discusses whole genome duplication [21]. There are three additional, as yet unpublished, spider genome assemblies available on NCBI (National Center for Biotechnology Information) (accession numbers: *Anelosimus studiosus*: GCA_008297655.1; *Latrodectus hesperus*: GCA_000697925.2; *Loxosceles reclusa*: GCA_001188405.1). Spider genomes are considered notoriously difficult to sequence, assemble, and annotate for a number of factors, including their relatively high repeat content, low guanine cytosine (GC) content [19] and due to the fact that they possess some extremely long coding genes in the

spidroin gene families [26,27]. Due to these challenges, the completeness of the available spider genomes varies greatly between assemblies (Supplementary Table 1). All of them are incomplete and there is no chromosome-level assembly published for any spider to date. While this does not lessen the conclusions of the above-mentioned studies, a chromosome-level assembly would open doors for more detailed studies on the genomic architecture of gene families, such as silk and venom genes, providing greater understanding of the evolutionary mechanisms driving the diversification of these gene families and genome evolution, in addition to the aforementioned applications in understanding adaptation and speciation.

The European wasp spider, *Argiope bruennichi* (Scopoli, 1772), is an orb-weaving spider in the family Araneidae (Figure 1). Despite the lack of a reference genome, *A. bruennichi* has been the focal species for studies on local adaptation, range expansion, admixture, and biogeography [5,28–30]. These studies have suggested that the range expansion and subsequent local adaptation of *A. bruennichi* to northern Europe was caused by genetic admixture. However, it is not yet known which regions of the genome are admixed, and if these regions are truly responsible for adaptation to colder climates. *A. bruennichi* has also been well studied in the context of dispersal and life history traits [31], as well as sexual selection and chemical communication (e.g. [32–36]). A high-quality reference genome would allow altogether new insights into our understanding of the genetic basis of these phenomena. Considering this background, a chromosome-level reference genome would be highly desirable for the species.

Sampling, DNA extraction and sequencing

Adult female *Argiope bruennichi* individuals were collected in the south of Portugal in 2013 and 2019 (Latitude: 37.739 N, Longitude: -7.853 E). As inbred lines of the species do not exist, we selected a population which was previously found to have low heterozygosity in the wild, likely due to naturally high levels of inbreeding [5].

For the baseline assembly, deoxyribonucleic acid (DNA) was extracted from a female collected in 2013 using the ArchivePure blood and tissue kit (5 PRIME, Hamburg, Germany), according to the manufacturer's protocol. A ribonucleic acid (RNA) digestion step was included using RNAse A solution (7000 U mL^{-1}; 5 PRIME). The DNA was stored at -80°C and subsequently sequenced in 2017 at the QB3 Genomics facility at the University of California Berkeley on a Pacific Biosciences Sequel I platform (PacBio, Menlo Park, CA, USA) on 10 cells. The sequencing yielded 21.5X coverage (approximately 36.65 gigabasepairs (Gb), with an estimated genome size of 1.7 Gb).

The specimen collected in 2019 was used to build a proximity-ligation based short-read library ("Hi-C"). Four Hi-C libraries were prepared from a single individual using Dovetail™ Hi-C library preparation kit according to the manufacturer's protocol (Dovetail Genomics, Santa Cruz, CA). The specimen was anesthetized with CO_2 before preparation. In brief, the legs were removed from the body and stored in liquid nitrogen, and the leg tissue was disrupted in liquid nitrogen using a mortar and pestle. Chromatin was fixed with formaldehyde, then extracted. Fixed chromatin was digested with DpnII, the 5' overhangs filled in with biotinylated nucleotides, and the free blunt ends were ligated. After ligation, crosslinks were reversed and the DNA purified to remove proteins. Purified DNA was treated to remove biotin that was not internal to ligated fragments. The DNA was then sheared to ~350 bp mean fragment size using a Covaris S2 Focused-ultrasonicator. A

typical Illumina library preparation protocol followed, with end repair and Illumina adapter ligation. Biotinylated fragments were captured with streptavidin beads before PCR (polymerase chain reaction) amplification (12 cycles), and size selection was performed using SPRI-select beads (Beckman Coulter GmbH, Germany) for a final library size distribution centered around 450 bp. The library was sequenced to approximately 440 million paired end reads on one Flowcell of an Illumina NextSeq 550 with a High Output v2 kit (150 cycles).

***De novo* genome assembly**

First, we generated a baseline assembly using 21.5X long-read Pacific Biosciences (PacBio) Sequel I sequencing data and the wtdbg2 assembler (v. 2.3) (WTDBG, RRID:SCR_017225) [37]. Next, we polished the assembly by applying three rounds of Pilon (v. 1.23) (Pilon, RRID:SCR_014731) [38] using ~30X of previously published Illumina paired-end data [5]. This resulted in 13,843 contigs with an N50 of 288.4 kilobase pairs (kb), and an overall assembly size of 1.67 gigabase pairs (Gb). Analysis of Benchmarking Universal Single Copy Orthologs (BUSCO) (v. 3.1.0) scores, using the arthropod data set (BUSCO, RRID:SCR_015008) [39], showed the presence of 90.2% of complete BUSCOs, with 86.4% complete and single-copy BUSCOs, 3.8% complete and duplicated BUSCOs, 3.3% fragmented BUSCOs, and 6.5% missing BUSCOs (Table 1). Next, we scaffolded the contigs using a proximity-ligation based short-read library [40]. Scaffolding using HiRise v. 2.1.7, a software pipeline designed specifically for using proximity ligation data to scaffold genome assemblies [40], resulted in 13 scaffolds over 1 megabase pairs (Mb) in size, comprising 98.4% of the assembly, with a genome assembly scaffold N50 of 124Mb and BUSCO scores of 91.1% complete genes (Figure

2, Table 1). Genome assembly statistics were calculated using QUAST v. 5.0.2 (QUAST, RRID:SCR_001228) [41] applying default parameters, except --min-contig 0. Previous studies have inferred the chromosome number of *A. bruennichi* to be 13, indicating our genome assembly is full-chromosome level [42,43].

Table 1: *Argiope bruennichi* genome assembly completeness

Genome assembly statistic	Unscaffolded	Scaffolded
**Assembly size**	1,669,116,561	1,670,285,661
**AT [a] / GC / N content (%)**	70.7 / 29.3 / 0	70.6 / 29.3 / 0.1
**Number of contigs / scaffolds**	13,843	2,231
**Longest contig / scaffold**	2,039,454	143,171,375
**Contig / scaffold N50**	288,395	124,235,998
**Contig / scaffold N90**	67,231	119,022,586
**% repetitive**	34.66	34.64
**BUSCO analysis [b]**		
Complete BUSCOs (%)	90.2	91.1
Complete and single-copy BUSCOs (%)	86.4	87.8
Complete and duplicated BUSCOs (%)	3.8	3.3
Fragmented BUSCOs (%)	3.3	2.8
Missing BUSCOs (%)	6.5	6.1

Genome assembly statistics were calculated using QUAST v. 5.0.2 (QUAST, RRID:SCR_001228) [41] using default parameters, except --min-contig 0.
[a] AT: adenine thymine
[b] BUSCO analysis using default parameters against the arthropod dataset

Repeat masking and removal of contaminants

The assembly was repeat-masked using a combination of the *de novo* repeat finder RepeatModeler (v. open-1.0.11) (RepeatModeler, RRID:SCR_015027) [44] and the

homology-based repeat finder RepeatMasker (v. open-4.0.9) (RepeatMasker, RRID:SCR_012954) [45]. Repetitive regions accounted for 34.64% of the genome assembly, of which the majority (20.52% of the genome) consisted of unclassified repeats, meaning that they have not been classified in previous studies. The remaining repetitive elements were made up of DNA elements (i.e. transposable elements: 6.27%), long interspersed nuclear elements (LINEs: 1.60%), simple repeats (i.e. duplications of 1-5 bp: 1.58%), long terminal repeat (LTR) elements (0.76%), satellites (0.63%), low complexity repeats (i.e. poly-purine or poly-pyrimidine stretches: 0.42%), and short interspersed nuclear elements (SINEs: 0.08%) (Table 2). BlobTools (v. 1.0) (Blobtools, RRID:SCR_017618) [46] was used to search for contamination, and subsequently mitochondrial sequences and bacterial scaffolds were removed from the assembly. The 14th-largest scaffold (Scaffold 839) matched the sequence of a recently-discovered bacterial symbiont of *Argiope bruennichi* [47].

Table 2: *Argiope bruennichi* repetitive DNA elements

Type of element	Number of elements	Length (bp)	Percentage of assembly
**SINEs**	4,643	1,314,740	0.08 %
**LINEs**	52,648	26,768,096	1.60 %
**LTR elements**	21,649	12,683,330	0.76 %
**DNA elements**	282,019	104,785,665	6.27 %
**Unclassified**	1,359,138	342,727,030	20.52 %
**Small RNA**	0	0	0.00 %
**Satellites**	28,474	10,495,658	0.63 %
**Simple repeats**	595,962	26,379,486	1.58 %
**Low complexity**	137,182	6,952,634	0.42 %
		**Total:**	34.64 %

Repetitive elements were classified using RepeatModeler (v. open-1.0.11) (Smit & Hubley, 2008) and RepeatMasker (v. open-4.0.9) (Smit & Hubley, 2013).

Genome annotation

Raw reads from previously published transcriptome sequencing data [5] were mapped against the repeat-masked assembly using HISAT2 (v. 2.1.0) (HISAT2, RRID:SCR_015530) [48]. After conversion of the resulting SAM file into a BAM file and subsequent sorting using SAMtools (v. 1.7) (SAMTOOLS, RRID:SCR_002105) [49], the sorted BAM file was converted to intron-hints for AUGUSTUS (v. 3.3.2) (Augustus, RRID:SCR_008417) [50] using AUGUSTUS scripts. AUGUSTUS was run on the soft-masked genome with the *Parasteatoda* parameter set. The resulting gff file containing predicted genes was converted into a gtf file using the AUGUSTUS script gtf2gff.pl. Additional AUGUSTUS scripts (getAnnoFastaFromJoinGenes.py and fix_in_frame_stop_codon_genes.py) were used to find and replace predicted genes containing in-frame stop codons with newly predicted genes. The resulting gtf file containing 23,270 predicted genes was converted to gff3 format using gtf2gff.pl and protein sequences of predicted genes were extracted with getAnnoFastaFromJoinGenes.py. Finally, functional annotation was performed using InterProScan (v. 5.39-77.0) (InterProScan, RRID:SCR_005829) [51,52] (Table 3).

Table 3: *Argiope bruennichi* genome annotation statistics

**Genome Annotation Statistic**	**Value**
**Number of protein coding genes**	23,270
**Functionally annotated genes (%)**	81.0
**Average exon length (bp)**	200
**Average intron length (bp)**	4,035
**BUSCO analysis** [a]	
Complete BUSCOs (%)	89.3

Complete and single-copy BUSCOs (%)	76.7
Complete and duplicated BUSCOs (%)	12.6
Fragmented BUSCOs (%)	7.0
Missing BUSCOs (%)	3.7

[a] BUSCO analysis using default parameters against the arthropod dataset

Comparative genomic analysis of repeat content

High repetitiveness is characteristic of spider genomes [19]. In order to compare the repeat content of *A. bruennichi* with that of other spiders, we downloaded the genome assemblies of several other spider species from NCBI and the DNA Data Bank of Japan (DDBJ) (accession numbers in Table 4), then treated them in the same manner as the *A. bruennichi* genome, masking the repeats using RepeatModeler (v. open-1.0.11) [44] and RepeatMasker (v. open-4.0.9) [45]. *Acanthoscurria geniculata* was excluded from this analysis due to the very large and relatively poorly assembled genome. The *A. bruennichi* genome has a slightly lower percentage of repetitive element content (34.64%) compared to most other spiders (Table 4). Some species, such as *Loxosceles reclusa, Trichonephila clavipes* (formerly *Nephila clavipes*), *Anelosimus studiosus* and *Parasteatoda tepidariorum,* have similar repetitive content (36.51%, 36.61%, 35.98% and 36.79% respectively); other species have much higher repetitive content, such as *Araneus ventricosus, Dysdera silvatica, Stegodyphus dumicola, Stegodyphus mimosarum* and *Pardosa pseudoannulata* (55.96%, 60.03%, 58.98%, 56.91% and 48.61% respectively). Only *Latrodectus hesperus* has lower repetitive content (20.97%). The classification and relative percentage of these repeats can be found in Supplementary Table 2 and Supplementary Figure 1.

Table 4: Total repetitive content in the genomes of spiders		
**Species**	**% repetitive**	**Accession number [reference]**
Argiope bruennichi	34.64	--
Araneus ventricosus	55.96	BGPR01000001-BGPR01300721 [a] [22]
Trichonephila clavipes	36.61	GCA_002102615.1 [b] [20]
Dysdera silvatica	60.03	GCA_006491805.1 [b] [25]
Stegodyphus dumicola	58.98	GCA_010614865.1 [b] [24]
Stegodyphus mimosarum	56.91	GCA_000611955.2 [b] [19]
Pardosa pseudoannulata	48.61	GCA_008065355.1 [b] [23]
Loxosceles reclusa	36.51	GCA_001188405.1 [b] [unpublished]
Anelosimus studiosus	35.98	GCA_008297655.1 [b] [unpublished]
Latrodectus hesperus	20.97	GCA_000697925.2 [b] [unpublished]
Parasteatoda tepidariorum	36.79	GCA_000365465.3 [b] [21]

Repetitive elements were classified using RepeatModeler (v. open-1.0.11) (Smit & Hubley, 2008) and RepeatMasker (v. open-4.0.9) (Smit & Hubley, 2013).

[a] DNA Data Bank of Japan (DDBJ)

[b] National Center for Biotechnology Information (NCBI)

Genome architecture of Hox, spidroin and venom genes

Previous studies on spider genomes have focused on whole genome duplication, silk gene evolution, and venom gene evolution [19–23]. Therefore, to place the *A. bruennichi* genome into the same context, we manually curated three gene sets from publicly available protein sequences: Hox, spidroin (silk), and venom genes. Because Hox genes are highly conserved across taxa [53], we chose the most complete sequences for the ten arthropod Hox gene classes from spiders without regard to the relatedness of the species to *A. bruennichi* (Supplementary Table 3). In contrast to Hox genes, spidroin and

venom genes are highly polymorphic and species-specific [54–57]. For the spidroin gene set, we downloaded protein sequences of the seven spidroin gene classes exclusively from five species of the genus *Argiope* (Supplementary Table 4). Venom genes are best studied in spiders that are medically significant to humans, which are very distant relatives to *A. bruennichi* [51–54]. To allow comparison, we focused on venom gene sequences available for araneid spiders (two species, Supplementary Table 5); however, the function and classification of these genes is poorly understood. With these three gene sets (Hox, spidroin, and venom), we performed a TBLASTN search against our genome assembly (v. 2.10.0+) (TBLASTN, RRID:SCR_011822) [62,63]. We recorded the genomic position of the best matches and compared them with the AUGUSTUS gene predictions for those locations. We employed a conservative E-value cutoff of less than 1.00E-20 and only included results with an identity greater than 60%. The manually curated FASTA files of each gene set used for the TBLASTN search are available in Supplementary Files 1-3. A table of the matches with accession numbers for each gene set is available in Supplementary Tables 3-5.

Whole genome duplication

In 2017, Schwager *et al.* asserted that a whole genome duplication (WGD) event occurred in the ancestor of scorpions and spiders, as evidenced by a high number of duplicated genes, including two clusters of Hox genes in *Parasteatoda tepidariorum* and the bark scorpion *Centruroides sculpturatus* [21]. In their study, they found one nearly-complete cluster of Hox genes on a single scaffold, lacking the *fushi tarazu (ftz)* gene, which they argued may be the case for this cluster in all spiders. The second set of Hox genes was distributed across two scaffolds, which the authors attributed to incompleteness of the

assembly due to patchy sequencing coverage [21]. For consistency, we will use the same nomenclature for Hox genes as used in [21] (*Abdominal-B: AbdB, Abdominal-A: AbdA, Ultrabithorax: Ubx, Antennapedia: Antp, fushi tarazu: ftz, sex combs reduced: scr, Deformed: Dfd, Hox3, proboscipedia: pb, labial: lab*). Corresponding with the results from *P. tepidariorum*, we found two clusters of Hox genes, with no evidence of tandem duplication. The two clusters occurred on two chromosomes (Chromosome 9 and Chromosome 6). In these locations, InterProScan generally annotated the genes as Hox genes but did not identify the specific type. On Chromosome 9, the Hox genes were in reverse collinear order, with no overlapping regions. Because it is complete, we will refer to this cluster as "Cluster A." On Chromosome 6, ("Cluster B") the genes were out of collinear order, with the position of *AbdA* and *Ubx* switched, and the coordinates for *Dfd, Hox3* and *pb* from the blast search overlapping (Figure 3A). The hits for *Antp* and *ftz* in Cluster B fell onto a single predicted gene in the annotation. Thus, it is unclear if *A. bruennichi* lacks one copy of *ftz*, as in *P. tepidariorum*, or if the annotation incorrectly fused the two genes in this cluster. In the study by Schwager *et al.,* 2017 [21]*,* low sequencing coverage of Cluster B downstream of *Dfd* limited their inference. In our genome assembly, by mapping the PacBio reads against the final assembly, we calculated that we have an average of more than 12X coverage across the length of both clusters, suggesting that Cluster B is not out of order due to problems arising from low coverage. It is possible that Hox Cluster B in spiders has changed or lost functionality following the ancestral WGD event.

<u>Spidroin genes</u>

There are seven classes of silk produced by araneomorph spiders, each with one or more unique uses; it is important to note that the uses of these silk types are best understood for spiders in the family Araneidae, and the number and uses of silk types can vary widely between families [20,22,64,65]. The classes of silk are major ampullate (*MaSp*) minor ampullate (*MiSp*), piriform (*PiSp*), aggregate (*AgSp*), aciniform (*AcSp*) tubuliform (also referred to as cylindrical) (*TuSp*) and flagelliform (*Flag*). In *A. bruennichi*, spidroin genes occur on eight out of the thirteen chromosome scaffolds (Chromosomes 1, 3, 4, 6, 8, 11, 12 and 13) (Figure 3B). There were no hits on smaller scaffolds. In the majority of cases, all blast matches for a single spidroin type occurred on a single chromosome; the only exception was for *AgSp*, which had hits on four different chromosomes. However, these were not all annotated as spidroins; on Chromosome 6 there were multiple hits which were annotated as spidroins, while on Chromosome 4 the hit was annotated as tropoelastin, on Chromosome 3 the hit was annotated as a chitin binding domain, and on Chromosome 8 the hit was annotated as a serine protease. All hits for *TuSp* occurred on Chromosome 1, but there were two physically separated clusters on the chromosome. There are more sequences available for *MaSp* than any of the other spidroin types in the genus *Argiope*, which allowed us to find matches for several unique *MaSp* genes in the *A. bruennichi* assembly. These occur in a small region of Chromosome 12, in close proximity to one another, suggesting that the spidroin genes in this group may have diversified via tandem duplication.

<u>Venom genes</u>

We found high identity matches for venom toxins on five of the chromosome scaffolds (Chromosomes 1, 2, 7, 10 and 11) (Figure 3B), but the majority of hits were on

Chromosome 1. Babb *et al.* 2017 conducted a study on silk genes in *Trichonephila clavipes* (formerly *Nephila clavipes*), in which they found a novel flagelliform-type gene (FLAG-b) which was expressed most highly in the venom glands, not the flagelliform silk glands. This added to previous findings in the *Stegodyphus mimosarum* genome, where spidroin-like proteins in the venom glands are found [19]. Interestingly, in the *A. bruennichi* genome assembly, there are several venom genes on Chromosome 11 in close proximity to flagelliform spidroin genes.

Conclusion

We have assembled and annotated the first chromosome-level genome for an arachnid. The assembly approach of combining long read, short read, and proximity ligation data overcame the challenges of assembling arachnid genomes, namely genome size, high repetitiveness, and low GC content. In our study, we made a preliminary analysis of the location of certain gene families of interest in the context of spider genomics, which hinted at several interesting directions for future studies on the evolution of silk and venom genes. Furthermore, because this species has undergone a recent and rapid range expansion, the well-resolved genome assembly will be useful for studies on the genomic underpinnings of range expansion and evolutionary adaptation to novel climates.

Availability of supporting data

The final genome assembly and raw data from the PacBio and Hi-C libraries, as well as the annotation, have been deposited at NCBI under BioProject PRJNA629526 and will be available upon publication. A publicly accessible genome browser hub with the

annotation and raw transcriptome and PacBio read coverage can be found on the UCSC Genome Browser server (hub name "Wasp spider hub").

Availability of source code and requirements

All data required to replicate this work are available on NCBI and in the supplementary files.

Declarations

List of abbreviations

Abd-A: Abdominal-A; Abd-B: Abdominal-B; AcSp: aciniform spidroin*; AgSp:* aggregate spidroin*; Antp: Antennapedia;* AT: adenine thymine*;* bp: basepairs*;* BUSCO: Benchmarking Universal Single Copy Orthologs*;* DDBJ: DNA Data Bank of Japan*; Dfd: Deformed;* DNA: deoxyribonucleic acid; *Flag:* flagelliform spidroin*; ftz: fushi tarazu;* Gb: gigabase pairs*;* GC: guanine cytosine*;* kb: kilobase pairs*; lab: labial;* LINE: long interspersed nuclear element*;* LTR: long terminal repeat*; MaSp:* major ampullate spidroin*;* Mb: megabase pairs*; MiSp:* minor ampullate spidroin*;* NCBI: National Center for Biotechnology Information*;* PacBio: Pacific Biosciences*; pb: proboscipedia;* PCR: polymerase chain reaction; *PiSp:* piriform spidroin*;* RNA: ribonucleic acid; *scr: sex combs reduced;* SINE: short interspersed nuclear element*; TuSp:* tubuliform spidroin*; Ubx: Ultrabithorax;* WGD: whole genome duplication

Consent for publication

Not applicable.

Competing interests

The authors declare that they have no competing interests.

Funding

Funding for this study was provided by the Deutsche Forschungsgemeinschaft (DFG) as part of the Research Training Group 2010 RESPONSE (GRK 2010) to GU.

Authors' contributions

MMS, HK, GU, and SP conceived of the study; MMS, HK, and GU collected the spiders. HK extracted DNA for the PacBio sequencing; MMS prepared and submitted the DNA for PacBio sequencing, with input and infrastructure provided by RGG. MMS and CJ constructed and sequenced the Hi-C library, with input and infrastructure provided by LJ and AK. MMS, AH and SP performed the genome assembly, and AH and KJH performed the genome annotation with input and infrastructure provided by MMS and SP. AH analyzed the repeat content of other spider genomes; MMS performed the analysis of whole genome duplication, spidroin genes, and venom genes. MMS, AH, KJH and SP wrote the first draft of the manuscript. All authors read and approved the final manuscript.

Acknowledgements

We would like to thank the California Academy of Sciences for allowing us access to their computing resources for the genome assembly, and to Dovetail Genomics for their support in troubleshooting the Hi-C kit and running HiRise. MMS thanks José Cerca for helpful ideas and discussions about the silk and venom gene analysis.

References

1. Wise DH. Spiders in Ecological Webs [Internet]. Cambridge: Cambridge University Press; 1993 [cited 2020 May 5]. Available from:

https://books.google.de/books?hl=en&lr=&id=qP0fYU3FTDwC&oi=fnd&pg=PR11&dq=wise+1993+spiders+in+ecological+webs&ots=qdaLh8EfxB&sig=H41AG9axkAJ-jfFYTL_8Z2OlKLg#v=onepage&q=wise 1993 spiders in ecological webs&f=false

2. Spiller DA, Schoener TW. Effects of top and intermediate predators in a terrestrial food web. Ecology [Internet]. Ecological Society of America; 1994 [cited 2020 May 5];75:182–96. Available from: http://doi.wiley.com/10.2307/1939393

3. Moulder BC, Reichle DE. Significance of spider predation in the energy dynamics of forest-floor arthropod communities. Ecol Monogr [Internet]. Wiley; 1972 [cited 2020 May 5];42:473–98. Available from: http://doi.wiley.com/10.2307/1942168

4. Wirta HK, Weingartner E, Hambäck PA, Roslin T. Extensive niche overlap among the dominant arthropod predators of the High Arctic. Basic Appl Ecol. Elsevier GmbH; 2015;16:86–92.

5. Krehenwinkel H, Rödder D, Tautz D. Eco-genomic analysis of the poleward range expansion of the wasp spider *Argiope bruennichi* shows rapid adaptation and genomic admixture. Glob Chang Biol. 2015;21:4320–32.

6. Garb JE, González A, Gillespie RG. The black widow spider genus *Latrodectus* (Araneae: Theridiidae): Phylogeny, biogeography, and invasion history. Mol Phylogenet Evol. Academic Press Inc.; 2004;31:1127–42.

7. Řezáč M, Arnedo MA, Opatova V, Musilová J, Řezáčová V, Král J. Taxonomic revision and insights into the speciation mode of the spider *Dysdera erythrina* species-complex (Araneae: Dysderidae): Sibling species with sympatric distributions. Invertebr Syst [Internet]. CSIRO; 2018 [cited 2020 Apr 29];32:10–54. Available from:

http://www.publish.csiro.au/?paper=IS16071

8. Vijay N, Bossu CM, Poelstra JW, Weissensteiner MH, Suh A, Kryukov AP, et al. Evolution of heterogeneous genome differentiation across multiple contact zones in a crow species complex. Nat Commun. Nature Publishing Group; 2016;7:1–10.

9. Turner TL, Hahn MW, Nuzhdin S V. Genomic islands of speciation in *Anopheles gambiae*. PLoS Biol [Internet]. 2005 [cited 2020 Apr 6];3:1572–8. Available from: www.plosbiology.org

10. Hejase HA, Salman-Minkov A, Campagna L, Hubisz MJ, Lovette IJ, Gronau I, et al. Genomic islands of differentiation in a rapid avian radiation have been driven by recent selective sweeps. bioRxiv. Cold Spring Harbor Laboratory; 2020;2020.03.07.977694.

11. Duranton M, Allal F, Fraïsse C, Bierne N, Bonhomme F, Gagnaire PA. The origin and remolding of genomic islands of differentiation in the European sea bass. Nat Commun. Nature Publishing Group; 2018;9:1–11.

12. Faria R, Navarro A. Chromosomal speciation revisited: Rearranging theory with pieces of evidence. Trends Ecol. Evol. Elsevier Current Trends; 2010. p. 660–9.

13. White MJD. Chromosomal rearrangements and speciation in animals [Internet]. Annu. Rev. Genet. 1969 [cited 2020 May 11]. p. 75–98. Available from: www.annualreviews.org

14. Rieseberg LH. Chromosomal rearrangements and speciation. Trends Ecol. Evol. Elsevier Current Trends; 2001. p. 351–8.

15. Noor MAF, Gratos KL, Bertucci LA, Reiland J. Chromosomal inversions and the reproductive isolation of species. Proc Natl Acad Sci U S A. National Academy of

Sciences; 2001;98:12084–8.

16. Yannic G, Basset P, Hausser J. Chromosomal rearrangements and gene flow over time in an inter-specific hybrid zone of the *Sorex araneus* group. Heredity (Edinb). Nature Publishing Group; 2009;102:616–25.

17. Feulner PGD, De-Kayne R. Genome evolution, structural rearrangements and speciation. Artic J Evol Biol [Internet]. 2017 [cited 2020 May 11];30:1488–90. Available from: https://www.researchgate.net/publication/318986085

18. Castiglia R. Sympatric sister species in rodents are more chromosomally differentiated than allopatric ones: Implications for the role of chromosomal rearrangements in speciation. Mamm Rev [Internet]. Blackwell Publishing Ltd; 2014 [cited 2020 May 11];44:1–4. Available from: http://doi.wiley.com/10.1111/mam.12009

19. Sanggaard KW, Bechsgaard JS, Fang X, Duan J, Dyrlund TF, Gupta V, et al. Spider genomes provide insight into composition and evolution of venom and silk. Nat Commun. Nature Publishing Group; 2014;5:3765.

20. Babb PL, Lahens NF, Correa-Garhwal SM, Nicholson DN, Kim EJ, Hogenesch JB, et al. The *Nephila clavipes* genome highlights the diversity of spider silk genes and their complex expression. Nat Genet. Nature Publishing Group; 2017;49:895–903.

21. Schwager EE, Sharma PP, Clarke T, Leite DJ, Wierschin T, Pechmann M, et al. The house spider genome reveals an ancient whole-genome duplication during arachnid evolution. BMC Biol. BMC Biology; 2017;15:1–27.

22. Kono N, Nakamura H, Ohtoshi R, Moran DAP, Shinohara A, Yoshida Y, et al. Orb-weaving spider *Araneus ventricosus* genome elucidates the spidroin gene catalogue.

Sci Rep [Internet]. Nature Publishing Group; 2019 [cited 2019 Jun 23];9:8380. Available from: http://www.nature.com/articles/s41598-019-44775-2

23. Yu N, Li J, Liu M, Huang L, Bao H, Yang Z, et al. Genome sequencing and neurotoxin diversity of a wandering spider *Pardosa pseudoannulata* (pond wolf spider). bioRxiv [Internet]. 2019;747147. Available from: http://biorxiv.org/content/early/2019/08/29/747147.abstract

24. Liu S, Aagaard A, Bechsgaard J, Bilde T. DNA methylation patterns in the social spider, *Stegodyphus dumicola*. Genes (Basel). 2019;10:137.

25. Sánchez-Herrero JF, Frías-López C, Escuer P, Hinojosa-Alvarez S, Arnedo MA, Sánchez-Gracia A, et al. The draft genome sequence of the spider *Dysdera silvatica* (Araneae, Dysderidae): A valuable resource for functional and evolutionary genomic studies in chelicerates. Gigascience. 2019;8:giz099.

26. Stellwagen SD, Renberg RL. Toward spider glue: Long read scaffolding for extreme length and repetitious silk family genes AgSp1 and AgSp2 with insights into functional adaptation. G3 Genes, Genomes, Genet. Genetics Society of America; 2019;9:1909–19.

27. Ayoub NA, Garb JE, Kuelbs A, Hayashi CY. Ancient properties of spider silks revealed by the complete gene sequence of the prey-wrapping silk protein (AcSp1). Mol Biol Evol [Internet]. 2013 [cited 2020 Jan 4];30:589–601. Available from: https://academic.oup.com/mbe/article-lookup/doi/10.1093/molbev/mss254

28. Krehenwinkel H, Tautz D. Northern range expansion of European populations of the wasp spider *Argiope bruennichi* is associated with global warming-correlated genetic

admixture and population-specific temperature adaptations. Mol Ecol. 2013;22:2232–48.

29. Wawer W, Rutkowski R, Krehenwinkel H, Lutyk D, Pusz- K. Population structure of the expansive wasp spider (*Argiope bruennichi*) at the edge of its range. J Arachnol. 2017;45:361–9.

30. Krehenwinkel H, Graze M, Rödder D, Tanaka K, Baba YG, Muster C, et al. A phylogeographical survey of a highly dispersive spider reveals eastern Asia as a major glacial refugium for Palaearctic fauna. J Biogeogr [Internet]. Wiley/Blackwell (10.1111); 2016 [cited 2018 Jun 21];43:1583–94. Available from: http://doi.wiley.com/10.1111/jbi.12742

31. Wolz M, Klockmann M, Schmitz T, Pekár S, Bonte D, Uhl G. Dispersal and life-history traits in a spider with rapid range expansion. Mov Ecol 2019 81. BioMed Central; 2020;8:1–11.

32. Fromhage L, Uhl G, Schneider JM. Fitness consequences of sexual cannibalism in female *Argiope bruennichi*. Behav Ecol Sociobiol [Internet]. Springer-Verlag; 2003 [cited 2019 Sep 1];55:60–4. Available from: http://link.springer.com/10.1007/s00265-003-0656-6

33. Schneider JM, Fromhage L, Uhl G. Extremely short copulations do not affect hatching success in *Argiope bruennichi* (Araneae, Araneidae). J Arachnol [Internet]. American Arachnological Society; 2005 [cited 2019 Sep 1];33:663–9. Available from: http://www.bioone.org/doi/abs/10.1636/S03-32.1

34. Schneider J, Uhl G, Herberstein ME. Cryptic female choice within the genus

Argiope: A comparative approach. In: Peretti A, Aisenberg A, editors. Cryptic Female Choice Arthropods Patterns, Mech Prospect [Internet]. Cham: Springer International Publishing; 2015 [cited 2018 Jul 26]. p. 55–77. Available from: http://link.springer.com/10.1007/978-3-319-17894-3_3

35. Chinta SP, Goller S, Lux J, Funke S, Uhl G, Schulz S. The sex pheromone of the wasp spider *Argiope bruennichi*. Angew Chemie - Int Ed [Internet]. Wiley-Blackwell; 2010 [cited 2018 Jul 26];49:2033–6. Available from: http://doi.wiley.com/10.1002/anie.200906311

36. Uhl G, Zimmer SM, Renner D, Schneider JM. Exploiting a moment of weakness: male spiders escape sexual cannibalism by copulating with moulting females. Sci Rep [Internet]. Nature Publishing Group; 2015 [cited 2018 Jul 26];5:16928. Available from: http://www.nature.com/articles/srep16928

37. Ruan J, Li H. Fast and accurate long-read assembly with wtdbg2. Nat Methods. Nature Research; 2020;17:155–8.

38. Walker BJ, Abeel T, Shea T, Priest M, Abouelliel A, Sakthikumar S, et al. Pilon: An integrated tool for comprehensive microbial variant detection and genome assembly improvement. Wang J, editor. PLoS One [Internet]. 2014 [cited 2020 Jan 4];9:e112963. Available from: https://dx.plos.org/10.1371/journal.pone.0112963

39. Simão FA, Waterhouse RM, Ioannidis P, Kriventseva E V., Zdobnov EM. BUSCO: assessing genome assembly and annotation completeness with single-copy orthologs. Bioinformatics [Internet]. 2015 [cited 2020 Feb 4];31:3210–2. Available from: https://academic.oup.com/bioinformatics/article-

lookup/doi/10.1093/bioinformatics/btv351

40. Putnam NH, O'Connell BL, Stites JC, Rice BJ, Blanchette M, Calef R, et al. Chromosome-scale shotgun assembly using an in vitro method for long-range linkage. Genome Res. Cold Spring Harbor Laboratory Press; 2016;26:342–50.

41. Gurevich A, Saveliev V, Vyahhi N, Tesler G. QUAST: Quality assessment tool for genome assemblies. Bioinformatics [Internet]. 2013 [cited 2020 Mar 24];29:1072–5. Available from: http://bioinf.spbau.ru/quast

42. Zhang YJ, Tong SJ. The routine method for preparing the chromosomes in spiders. Chinese J Zool. 1990;25:30–1.

43. Araujo D, Mattos VF, Giroti AM, Kraeski MG, Carvalho LS, Brescovit AD. Cytogenetical characterization of six orb-weaver species and review of cytogenetical data for Araneidae. J Arachnol. 2011;39:337–44.

44. Smit AFA, Hubley R. RepeatModeler-1.0 [Internet]. 2008. Available from: http://www.repeatmasker.org

45. Smit AFA, Hubley R. RepeatMasker-4.0 [Internet]. 2013. Available from: http://www.repeatmasker.org

46. Laetsch DR, Blaxter ML. BlobTools: Interrogation of genome assemblies. F1000Research. F1000 Research, Ltd.; 2017;6:1287.

47. Sheffer MM, Uhl G, Prost S, Lueders T, Urich T, Bengtsson MM. Tissue- and population-level microbiome analysis of the wasp spider *Argiope bruennichi* identified a novel dominant bacterial symbiont. Microorganisms [Internet]. 2020 [cited 2020 Jan 4];8:8. Available from: https://www.mdpi.com/2076-2607/8/1/8

48. Kim D, Paggi JM, Park C, Bennett C, Salzberg SL. Graph-based genome alignment and genotyping with HISAT2 and HISAT-genotype. Nat Biotechnol. Nature Publishing Group; 2019;37:907–15.

49. Li H, Handsaker B, Wysoker A, Fennell T, Ruan J, Homer N, et al. The Sequence Alignment/Map format and SAMtools. Bioinformatics [Internet]. 2009 [cited 2020 Jan 8];25:2078–9. Available from: https://academic.oup.com/bioinformatics/article-lookup/doi/10.1093/bioinformatics/btp352

50. Hoff KJ, Stanke M. Predicting Genes in Single Genomes with AUGUSTUS. Curr Protoc Bioinforma [Internet]. 2018 [cited 2020 Jan 8];e57. Available from: https://onlinelibrary.wiley.com/doi/abs/10.1002/cpbi.57

51. Jones P, Binns D, Chang HY, Fraser M, Li W, McAnulla C, et al. InterProScan 5: Genome-scale protein function classification. Bioinformatics [Internet]. 2014 [cited 2020 Jan 15];30:1236–40. Available from: https://academic.oup.com/bioinformatics/article-lookup/doi/10.1093/bioinformatics/btu031

52. Quevillon E, Silventoinen V, Pillai S, Harte N, Mulder N, Apweiler R, et al. InterProScan: Protein domains identifier. Nucleic Acids Res [Internet]. 2005 [cited 2020 Jan 15];33:W116–20. Available from: https://academic.oup.com/nar/article-lookup/doi/10.1093/nar/gki442

53. Pearson JC, Lemons D, McGinnis W. Modulating Hox gene functions during animal body patterning. Nat. Rev. Genet. Nature Publishing Group; 2005. p. 893–904.

54. Gatesy J, Hayashi C, Motriuk D, Woods J, Lewis R. Extreme diversity, conservation, and convergence of spider silk fibroin sequences. Science (80-). American Association

for the Advancement of Science; 2001;291:2603–5.

55. Hayashi CY, Shipley NH, Lewis R V. Hypotheses that correlate the sequence, structure, and mechanical properties of spider silk proteins. Int J Biol Macromol. Elsevier; 1999. p. 271–5.

56. Casewell NR, Wü Ster W, Vonk FJ, Harrison RA, Fry BG. Complex cocktails: the evolutionary novelty of venoms. Trends Ecol Evol [Internet]. 2013 [cited 2020 May 11];28:219–29. Available from: http://dx.doi.org/10.1016/j.tree.2012.10.020

57. Fry BG, Roelants K, Champagne DE, Scheib H, Tyndall JDA, King GF, et al. The Toxicogenomic Multiverse: Convergent Recruitment of Proteins Into Animal Venoms. 2009 [cited 2020 May 11]; Available from: www.annualreviews.org

58. Grishin E. Polypeptide neurotoxins from spider venoms. Eur J Biochem [Internet]. John Wiley & Sons, Ltd; 1999 [cited 2020 May 11];264:276–80. Available from: http://doi.wiley.com/10.1046/j.1432-1327.1999.00622.x

59. Escoubas P. Molecular diversification in spider venoms: A web of combinatorial peptide libraries. Mol. Divers. 2006. p. 545–54.

60. Escoubas P, Sollod B, King GF. Venom landscapes: Mining the complexity of spider venoms via a combined cDNA and mass spectrometric approach. Toxicon. Pergamon; 2006;47:650–63.

61. Diniz CR, do Nascimento Cordeiro M, Junor LR, Kelly P, Fischer S, Reimann F, et al. The purification and amino acid sequence of the lethal neurotoxin Tx1 from the venom of the Brazilian ‘armed’ spider *Phoneutria nigriventer*. FEBS Lett [Internet]. 1990 [cited 2020 May 11];263:251–3. Available from: http://doi.wiley.com/10.1016/0014-

5793%2890%2981386-3

62. Gerts EM, Yu YK, Agarwala R, Schäffer AA, Altschul SF. Composition-based statistics and translated nucleotide searches: Improving the TBLASTN module of BLAST. BMC Biol. BioMed Central; 2006;4:41.

63. Altschul SF, Gish W, Miller W, Myers EW, Lipman DJ. Basic local alignment search tool. J Mol Biol. Academic Press; 1990;215:403–10.

64. Vollrath F. Biology of spider silk. Int J Biol Macromol [Internet]. 1999;24:81–8. Available from: https://www.sciencedirect.com/science/article/pii/S0141813098000762

65. Blackledge TA, Hayashi CY. Silken toolkits: Biomechanics of silk fibers spun by the orb web spider *Argiope argentata* (Fabricius 1775). J Exp Biol. The Company of Biologists Ltd; 2006;209:2452–61.

66. Durand NC, Robinson JT, Shamim MS, Machol I, Mesirov JP, Lander ES, et al. Juicebox Provides a Visualization System for Hi-C Contact Maps with Unlimited Zoom. Cell Syst. Cell Press; 2016;3:99–101.

Figure Legends

Figure 1: Female *Argiope bruennichi* spider in orb web from Loulé (Faro, Portugal). Photo credit: Monica M. Sheffer

Figure 2: Genome assembly completeness. (A) Contact heatmap of Hi-C scaffolding shows long-range contacts of paired-end Hi-C reads. Gray lines denote scaffold (chromosome) boundaries. Visualized with Juicebox (v. 1.11.08) [66]. (B) Cumulative length of assembly contained within contigs. Note that the vast majority (98.4%) of the genome is contained within very few (13) contigs. Visualized with QUAST v. 5.0.2 [41] using default parameters, except --min-contig 0.

Figure 3: Schematic representation of location of gene families on the 13 chromosomes. (A) Hox gene clusters. Genes connected by a black line occur on the same scaffold. Cluster A occurs on Chromosome 9, and Cluster B occurs on Chromosome 6. The presence of two Hox gene clusters on two chromosomes validates the previous finding of whole genome duplication [21]. (B) Position of Hox, spidroin and venom genes on chromosome scaffolds. The light grey bars represent chromosomes, the colored rectangles represent the seven different spidroin gene families, the black rectangles represent venom genes, and the white rectangles represent Hox gene clusters.

Figures

Figure 1:

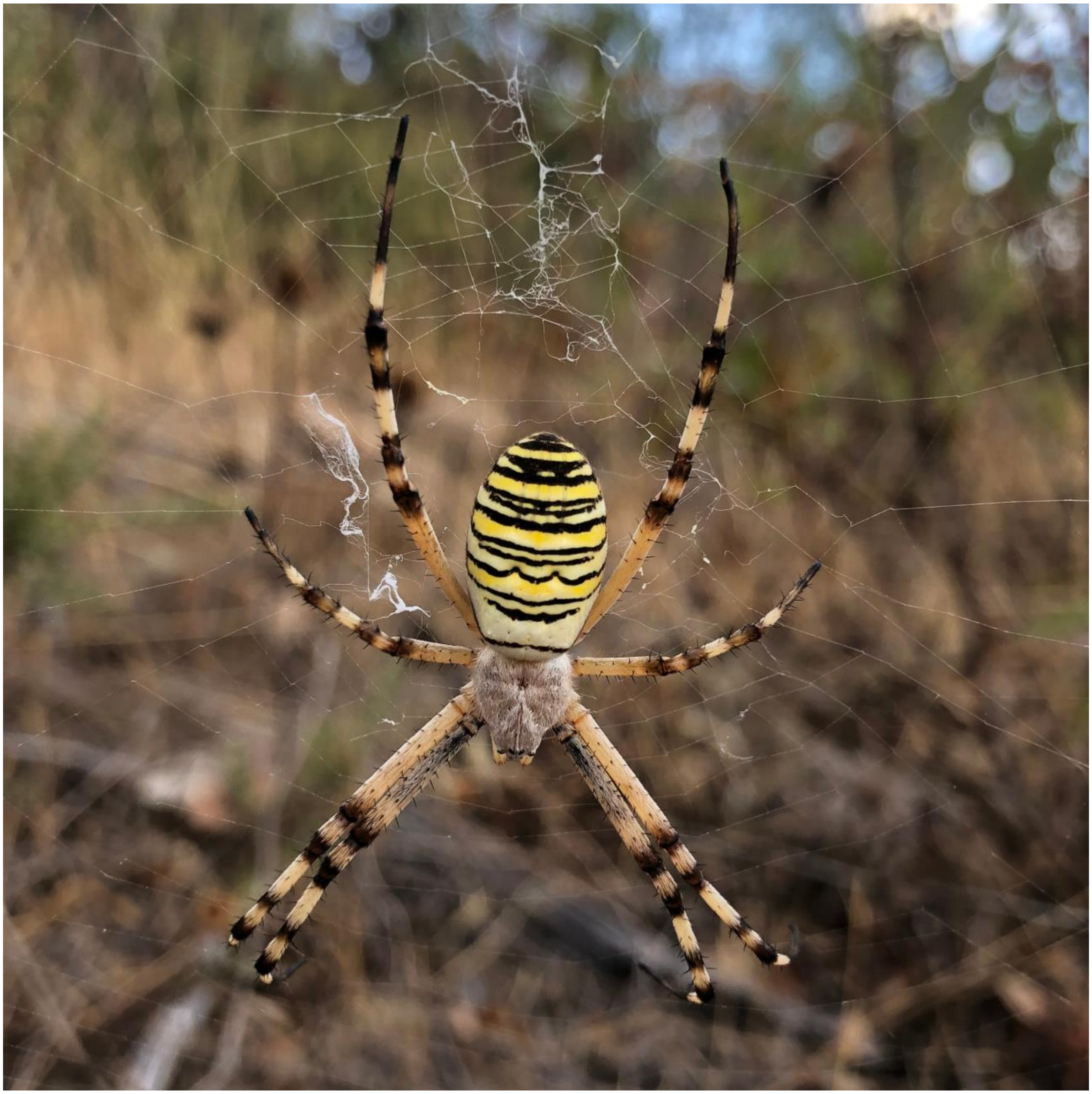

Figure 2:

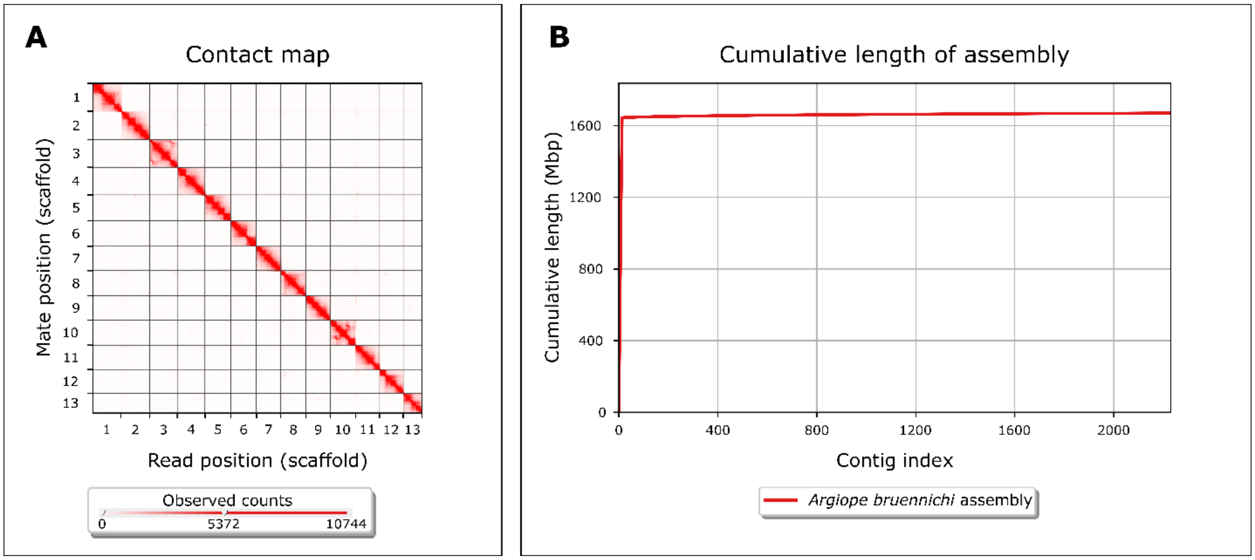
A
Contact map
Mate position (scaffold)
1
2
3
4
5
6
7
8
9
10
11
12
13
Read position (scaffold)
Observed counts
0
5372
10744
B
Cumulative length of assembly
Cumulative length (Mbp)
0
400
800
1200
1600
2000
Contig index
Argiope bruennichi assembly

Figure 3:

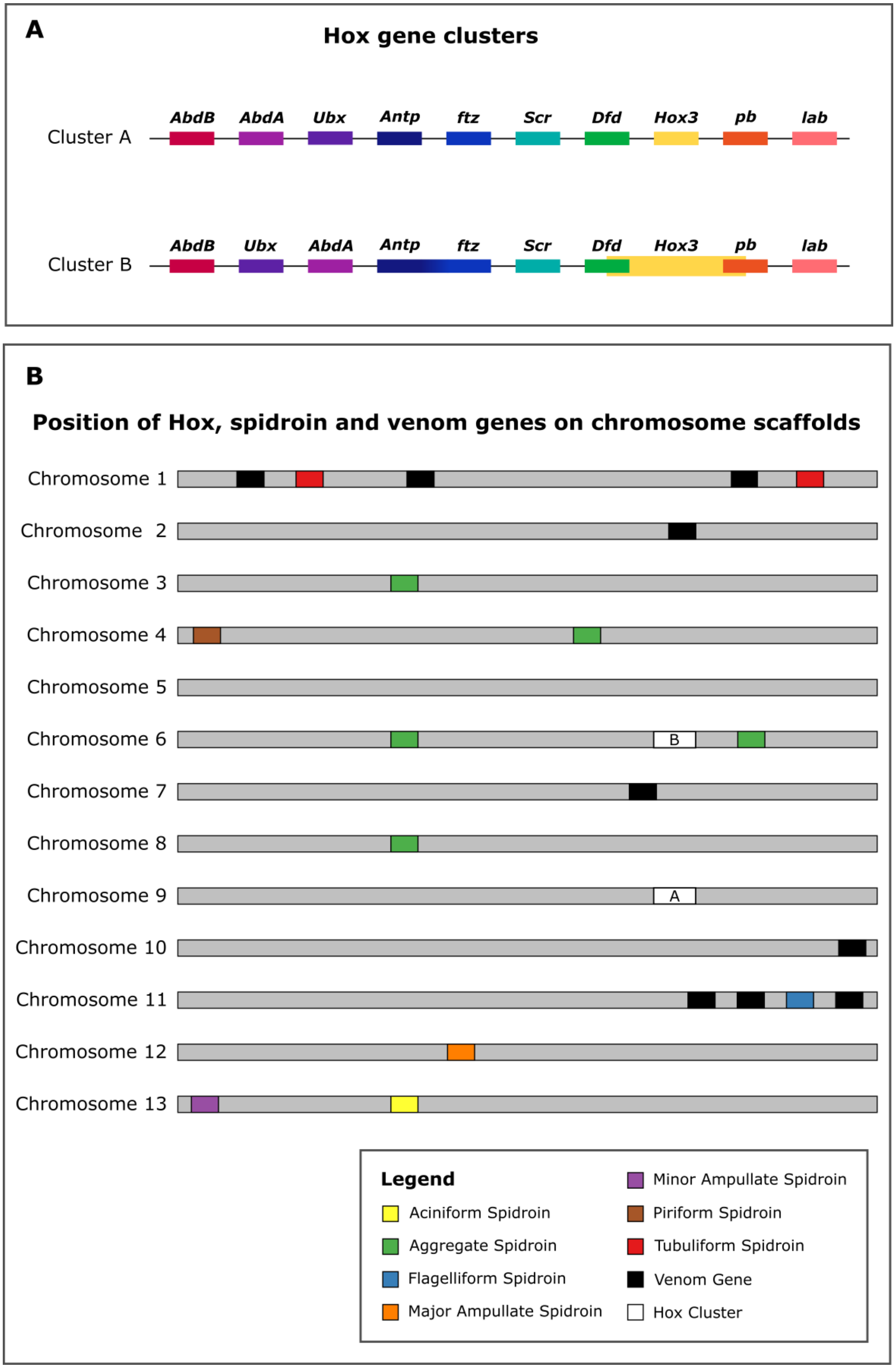
A
Hox gene clusters
Cluster A
AbdB
AbdA
Ubx
Antp
ftz
Scr
Dfd
Hox3
pb
lab
Cluster B
AbdB
Ubx
AbdA
Antp
ftz
Scr
Dfd
Hox3
pb
lab
B
Position of Hox, spidroin and venom genes on chromosome scaffolds
Chromosome 1
Chromosome 2
Chromosome 3
Chromosome 4
Chromosome 5
Chromosome 6
B
Chromosome 7
Chromosome 8
Chromosome 9
A
Chromosome 10
Chromosome 11
Chromosome 12
Chromosome 13
Legend
Aciniform Spidroin
Aggregate Spidroin
Flagelliform Spidroin
Major Ampullate Spidroin
Minor Ampullate Spidroin
Piriform Spidroin
Tubuliform Spidroin
Venom Gene
Hox Cluster

585

Figure 1

Click here to download Figure
Figure1_Argiope_bruennichi_MertolaPortugal.JPG

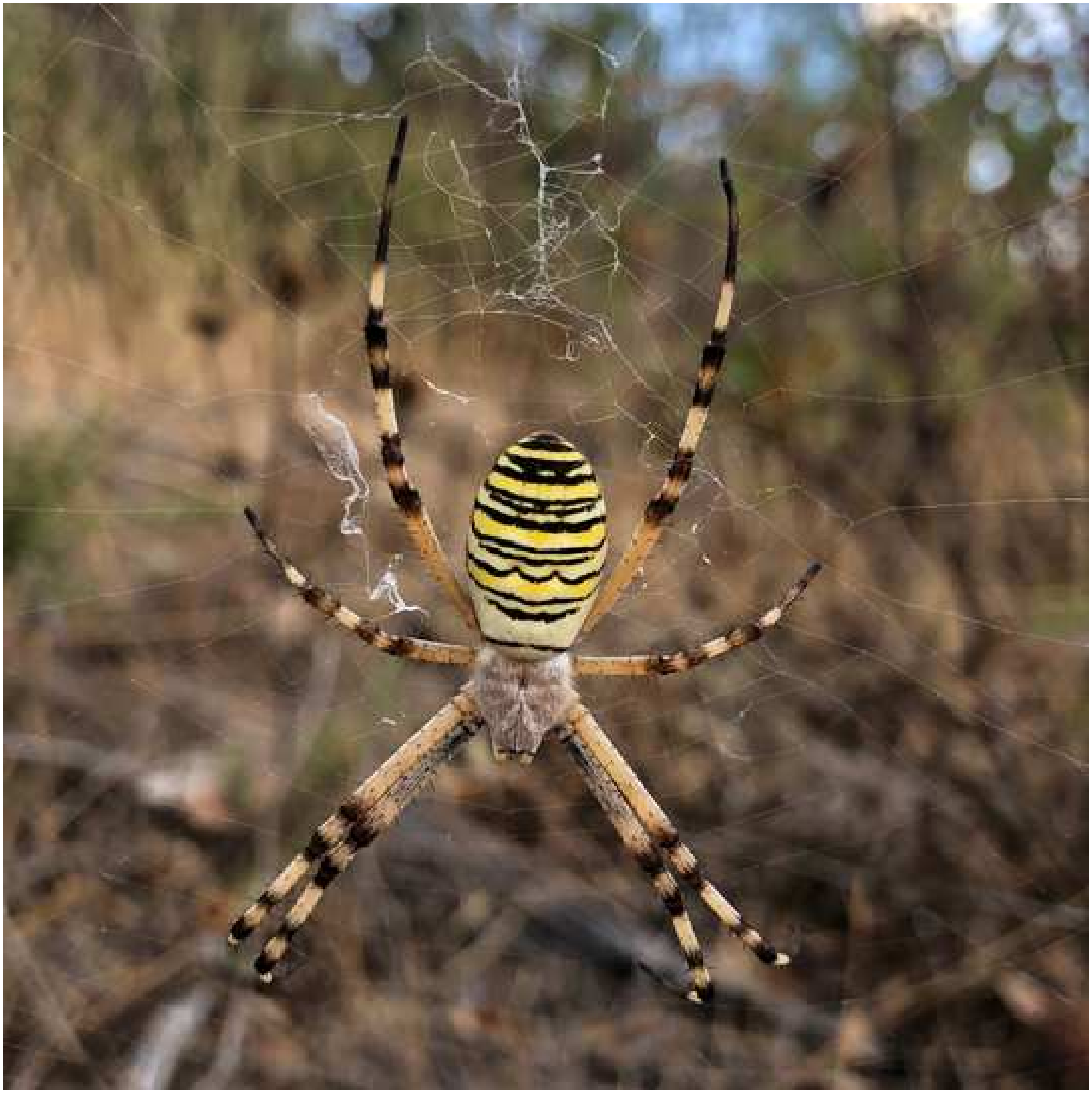

Figure 2

Click here to download Figure Figure2_AssemblyCompleteness.png

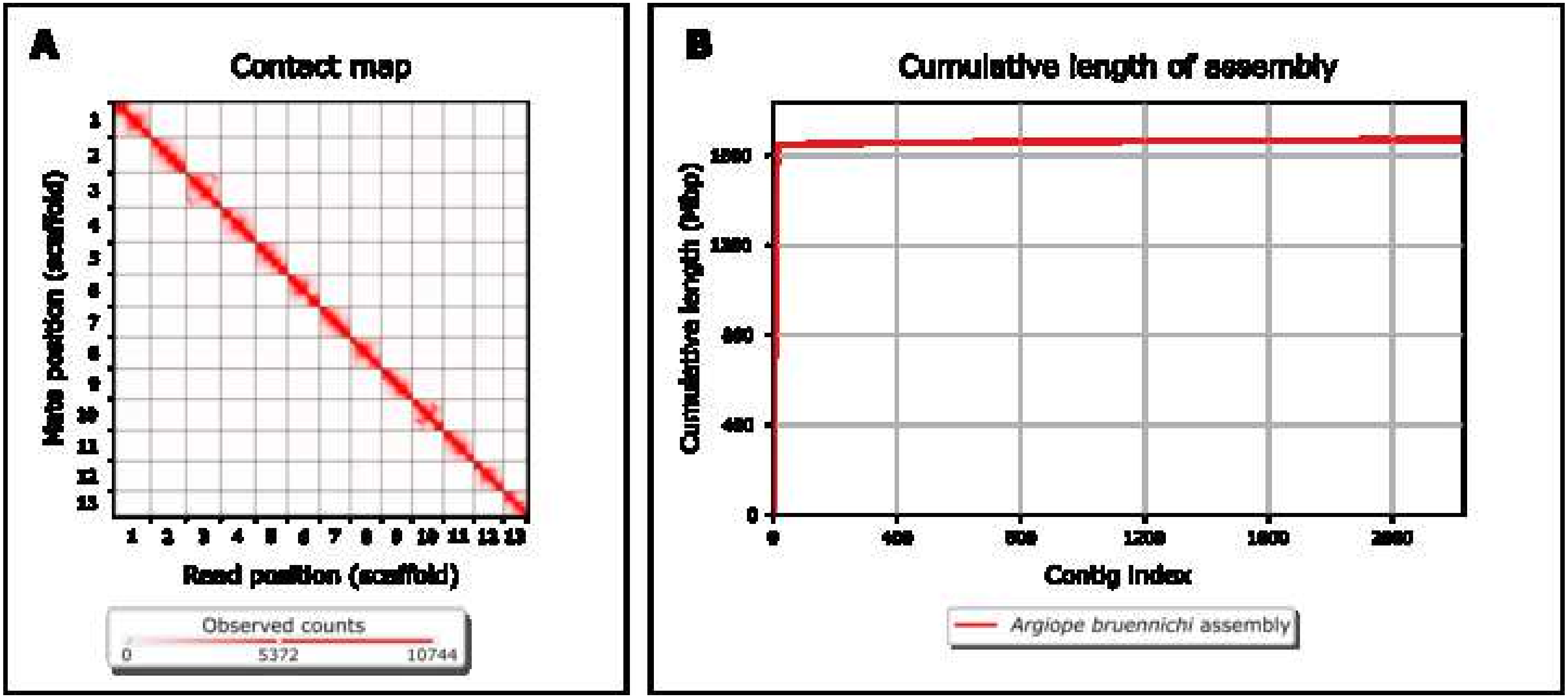

Figure 3

Click here to download Figure Figure3_GenomeArchitecture.png

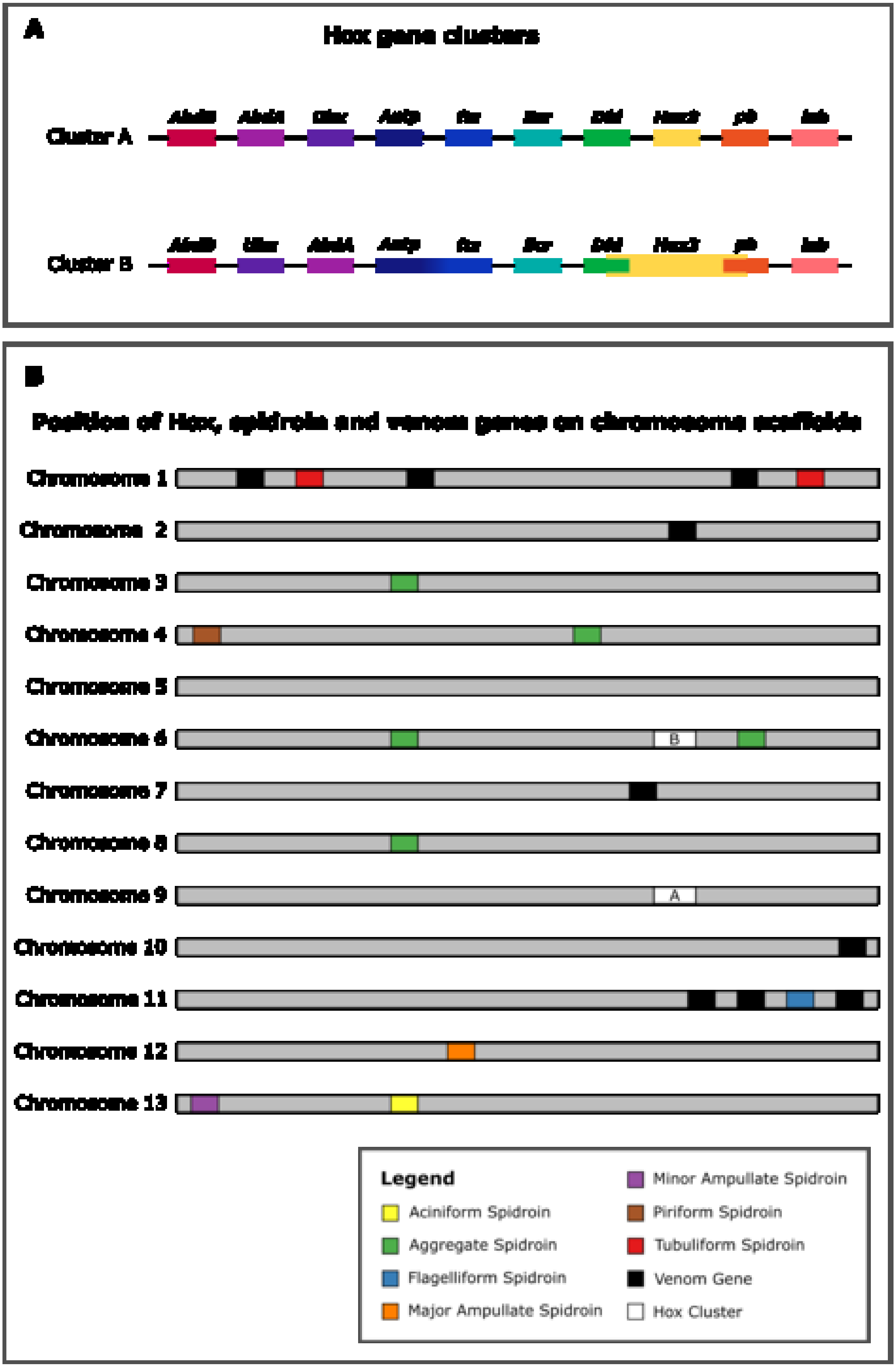

Supplementary File 1

Click here to access/download
Supplementary Material
SupplementaryFile1_hox_blastResults.fasta

Supplementary File 2

Click here to access/download

Supplementary Material

SupplementaryFile2_spidroin_blastResults.fasta

Supplementary File 3

Click here to access/download
Supplementary Material
SupplementaryFile3_venom_blastResults.fasta

Supplementary Figure 1

Click here to access/download
Supplementary Material
SupplementaryFigure1_RepeatContent.png

Supplementary Table 1

Click here to access/download
Supplementary Material
SupplementaryTable1_SpiderGenomeAssemblies.xlsx

Supplementary Table 2

Click here to access/download
Supplementary Material
SupplementaryTable2_RepetitiveContentSpiders.xlsx

Supplementary Table 3

Click here to access/download
Supplementary Material
SupplementaryTable3_hox_blastResults.xlsx

Supplementary Table 4

Click here to access/download
Supplementary Material
SupplementaryTable4_spidroin_blastResults.xlsx

Supplementary Table 5

Click here to access/download

Supplementary Material

SupplementaryTable5_venom_blastResults.xlsx
