# Supplementary material for: Chromosome-level reference genome of the European wasp spider Argiope bruennichi: a resource for studies on range expansion and evolutionary adaptation
Source: Gigascience. 2021 Jan 7;10(1):giaa148. doi: 10.1093/gigascience/giaa148 (PMC7788392; doi:10.1093/gigascience/giaa148)
Supplement: giaa148_GIGA-D-20-00146_Revision_1 [file giaa148_giga-d-20-00146_revision_1.pdf]

GigaScience

Chromosome-level reference genome of the European wasp spider Argiope bruennichi: a resource for studies on range expansion and evolutionary adaptation

--Manuscript Draft--

Manuscript Number:	GIGA-D-20-00146R1
Full Title:	Chromosome-level reference genome of the European wasp spider Argiope bruennichi: a resource for studies on range expansion and evolutionary adaptation
Article Type:	Data Note
Funding Information:	Deutsche Forschungsgemeinschaft (GRK 2010) — Dr. Gabriele Uhl
Abstract:	Background<br><br>Argiope bruennichi , the European wasp spider, has been investigated intensively as a focal species for studies on sexual selection, chemical communication, and the dynamics of rapid range expansion at a behavioral and genetic level. However, the lack of a reference genome has limited insights into the genetic basis for these phenomena. Therefore, we assembled a high-quality chromosome-level reference genome of the European wasp spider as a tool for more in-depth future studies.<br><br>Findings<br><br>We generated, de novo , a 1.67Gb genome assembly of A. bruennichi using 21.5X PacBio sequencing, polished with 30X Illumina paired-end sequencing data, and proximity ligation (Hi-C) based scaffolding. This resulted in an N50 scaffold size of 124Mb and an N50 contig size of 288kb. We found 98.4% of the genome to be contained in 13 scaffolds, fitting the expected number of chromosomes (n = 13). Analyses showed the presence of 91.1% of complete arthropod BUSCOs, indicating a high quality assembly.<br><br>Conclusions<br><br>We present the first chromosome-level genome assembly in the order Araneae. With this genomic resource, we open the door for more precise and informative studies on evolution and adaptation not only in A. bruennichi , but also in arachnids overall, shedding light on questions such as the genomic architecture of traits, whole-genome duplication and the genomic mechanisms behind silk and venom evolution.
Corresponding Author:	Monica M Sheffer<br>Zoological Institute and Museum, University of Greifswald, Germany<br>Greifswald, GERMANY
Corresponding Author Secondary Information:	
Corresponding Author's Institution:	Zoological Institute and Museum, University of Greifswald, Germany
Corresponding Author's Secondary Institution:	
First Author:	Monica M. Sheffer
First Author Secondary Information:	
Order of Authors:	Monica M. Sheffer
	Anica Hoppe
	Henrik Krehenwinkel
	Gabriele Uhl
	Andreas W. Kuss
	Lars Jensen
	Corinna Jensen

Powered by Editorial Manager® and ProduXion Manager® from Aries Systems Corporation

	Rosemary G. Gillespie
	Katharina J. Hoff
	Stefan Prost
**Order of Authors Secondary Information:**	
**Response to Reviewers:**	Comments to the editor:<br><br>I am re-submitting our data note manuscript entitled, “Chromosome-level reference genome of the European wasp spider Argiope bruennichi: a resource for studies on range expansion and evolutionary adaptation” by Monica M. Sheffer, Anica Hoppe, Henrik Krehenwinkel, Gabriele Uhl, Andreas W. Kuss, Lars Jensen, Corinna Jensen, Rosemary G. Gillespie, Katharina J. Hoff and Stefan Prost (shared last authorship, KJH and SP), following major revision.<br>Firstly, we would like to thank both reviewers for their insightful comments, which we feel helped us to improve the manuscript substantially. We addressed the specific points of the reviewers in response letters to each of them, below. However, there are some general changes to the manuscript that were not specifically requested, which are outlined here:<br><br>1) Formatting of title page and table with author emails (we did not track formatting changes, as this looked very messy, but actual additions of information are tracked)<br>2) Addition of city to author affiliation information (lines 14-27)<br>3) We can no longer claim to have published the first chromosome-level genome for an arachnid, as recently six tick genomes have been published at chromosome level. Therefore, we have changed the text to reflect that this is the first chromosome-level genome assembly for a spider (order Araneae) (lines 46-47, 402-403).<br>4) We rearranged and added one new panel to our assembly completeness figure (figure 2B), which shows the size of the 20 largest scaffolds. This demonstrates that the 13 ‘chromosome’ super scaffolds are dramatically larger than the next largest, and that there are no large missing pieces.<br>5) According to formatting guidelines of the journal, we removed embedded titles from our figures.<br>6) In the final version of our assembly, no bacterial or mitochondrial sequences were found, so we adjusted phrasing in lines 224-226 to reflect that.<br>7) Some small changes to grammar and word choice have also been made.<br><br>With the field of arachnid genomics advancing so quickly, we are hopeful that the revisions to our manuscript are satisfactory, as a quick decision would allow us to publish our dataset while it is still the first chromosome-level genome for a spider and will be valued and cited as such.<br><br>Sincerely,<br>Monica M. Sheffer, on behalf of all co-authors<br><br>-----------<br><br>Authors’ reply to the Review Report (Reviewer 1):<br><br>We thank Reviewer 1 for the input on and subsequent improvement of our manuscript, and have answered their questions here and amended the manuscript to address them. Changes to the manuscript are given here with line numbers and excerpts from the text, and indicated in the text using track changes. Line numbers correspond to the manuscript with “All Markup” showing in track changes.<br><br>Reviewer point one:<br>Minor point, but the genomes of L. hesperus and L. reclusa have been analyzed, "published" and discussed along with other pilot genomes of the i5k project in a paper by Thomas et al. (2020) in Genome Biology (see: https://genomebiology.biomedcentral.com/articles/10.1186/s13059-019-1925-7)<br>It is more that these species' genomes haven't been published and discussed in their own single genome specific paper. It would be nice to cite the aforementioned paper to credit the i5k work.

Powered by Editorial Manager® and ProduXion Manager® from Aries Systems Corporation

Authors' response:
Thank you for drawing our attention to this oversight. We have updated the text (lines 91-94) and Supplementary Table 1 to reflect the publication of these spider genomes: "To the best of our knowledge, ten draft spider genomes have been published to date [7,27–33], most of which focus on silk and venom genes, while one discusses whole-genome duplication [7] and the publication of the most recent two focuses on gene content evolution across arthropods [33]. There is one additional, as yet unpublished, spider genome assembly available on NCBI (National Center for Biotechnology Information) (Anelosimus studiosus, accession number: GCA_008297655.1)."

Reviewer point two:
On page 3, line 63 the authors discuss why spider genomes are notoriously difficult to assemble They mention high repeat content , low GC content and long spidroin genes. I was surprised that they did not mention that spider genomes are likely to be highly polymorphic (have high heterozygosity), and the difficulty of assembling heterozygous genomes, and that it is not easy to make inbred lines of spiders. Given the authors specifically pick an individual from a population with low heterozygosity, it seems they recognize this as a problem too, so perhaps they should mention this being part of the problem of assembly.

Authors' response:
Indeed, this is a challenge that we failed to mention in the introduction; we have added this to the text in lines 100-101:
"Spider genomes are considered notoriously difficult to sequence, assemble, and annotate for a number of factors, including their relatively high repeat content, low guanine cytosine (GC) content, high levels of heterozygosity in the wild [27]..."

Reviewer point three:
The Babb et al. 2017 paper should probably also be cited along with the other references on line 66 (it also provides a comprehensive sense of spidroin gene lengths)

Authors' response:
Thank you for noticing this omission. We have added the reference to line 102, and additionally added the reference to Kono et al. 2019, which is also relevant here: "…they possess some extremely long coding genes in the spidroin gene families [28,29,34,35]."

Reviewer point four:
The authors should provide more detail on the library preparation methods for the PacBio genomic DNA libraries prior to sequencing. What was the length of the DNA insert sizes sequenced, what type of size selection methods were employed to restrict the sequencing to large fragments? This is important for people that would like to replicate the methods and maximize the utility of this publication. How long were the movie lengths of the SMRT cells?

Authors' response:
We included additional information about the library preparation for PacBio sequencing, lines 132-135:
"The DNA was stored at -80°C until library preparation in 2017. The DNA extract was cleaned using a salt:PCI cleaning step, and had a fragment size distribution from 1,300-165,500 bp (peak at 14,002 bp) before size selection. The library was size selected to 15 kilobasepairs (kb) using Pippin prep…"
We do not have any information on the movie lengths, but if this is critical information for the reviewer, we can contact the sequencing facility for more details.
One additional change is to the sequencing year: we noticed in our notes that while we submitted the DNA for library prep and sequencing in 2017, the actual sequencing was performed by the facility in 2018. This has been updated in line 135:
"…and subsequently sequenced in 2018 at the QB3 Genomics facility at the University of California Berkeley on a Pacific Biosciences Sequel I platform (PacBio, Menlo Park, CA, USA) on 10 cells."

Reviewer point five:
On line 123 can the authors provide the NCBI SRA accession numbers for the Illumina

data (from reference 5) used for genome polishing. Was a specific subset of Illumina reads published with reference 5 used for the polishing and if so what geographic population did that individual come from and how many individuals was the data derived from?

Authors' response:
Thank you for pointing this out, we realized after submission that the accession number was not in the text. We have added the accession number, and information about the sequenced individual in lines 159-161:
"…previously published Illumina paired-end data derived from a single female individual from a population in Madeira (SRA accession number: ERX533198) [5]…"

Reviewer point six:
I find it a little confusing that the authors do not state the total number scaffolds assembled in the text of the paper but I assume it is listed in Table 1 as 2231 scaffolds. The text says that scaffolding resulted in 13 scaffolds over 1Mb in size. So my interpretation is that there were 2231 scaffolds, and 13 of these were over 1Mb in size. I think the authors should clarify this in the text, in other words most of the genome is in these 13 large pieces but there are still many additional remaining pieces. As a follow up, I think it would be helpful for the authors to discuss what is going on with these remaining pieces (do they contain genes?) and provide more detail on them such as a histogram of the size distribution of the smaller scaffolds, otherwise it is hard to visualize what this data looks like

Authors' response:
This is a very good point. We have added more information on our choice of naming the 13 largest scaffolds as chromosomes, and on the sizes of the lesser scaffolds in the text and a new panel of figure 2 (lines 201-206 (quoted below), Figure 2B), as well as a supplementary histogram which shows all of the lesser scaffold sizes, Supplementary Figure 1. We moved our reference to the bacterial scaffold into this section (de novo genome assembly) as well. We have also provided information on the small number of transcripts predicted on lesser scaffolds in the section on genome annotation (lines 252-253).
"The 13 largest scaffolds are thus henceforth referred to as Chromosomes 1-13, ordered according to size (Figure 2B). The 14th-largest scaffold (Scaffold 839) contained the 16S sequence of a recently discovered, as yet unnamed, bacterial symbiont of A. bruennichi [48]. The remaining 2,217 scaffolds are much smaller, ranging from 1,747-258,743 bp in length (Supplementary Figure 1) and will henceforth be referred to as "lesser scaffolds"."

"The majority of annotated genes fall on the 13 chromosome scaffolds, although 272 transcripts were predicted on the lesser scaffolds."

Reviewer point seven:
I really like the tables and figures of the amount of repetitive DNA content in different spider genomes. Given the earlier statement that spider genomes are difficult to assemble due to their repetitiveness (line 64), I think it would be useful to broaden the context and also compare spider repeat content to that of other arthropods to determine if spiders are an outlier or this was a misconception.

Authors' response:
Thank you for this suggestion; it provides an interesting dimension to the paper. Because some repeat masking programs are prone to under-masking, we included 10 non-spider arthropod species which used RepeatModeler for generating a custom species-specific repeat library and RepeatMasker for masking (as we did) for quantifying repeat content, and downloaded an additional 4 genomes to mask ourselves, representing a broad taxonomic sampling, although narrow within taxa. Repeat masking is very computationally expensive, which is why we chose a small number. The results of our analysis can be found in the text (lines 275-295) and in Table 4, which we extended to include all of the newly-investigated species:
"It is often asserted that the repeat content in spiders is higher in general than in other arthropod groups [i.e. 27]. In order to test this assertion, we looked into the repeat content in genomes of additional arthropod species. We obtained repeat content estimates, for which the repeats were masked using RepeatModeler and

RepeatMasker, for three insect species (Bombus terrestris, Drosophila melanogaster and Rhodnius prolixus [71]), and seven tick and mite species (Ixodes persulcatus, Haemaphysalis longicornis, Dermacentor silvarum, Hyalomma asiaticum, Rhipicephalus sanguineus, and Ixodes scapularus [72]). We additionally downloaded the genomes of four more arthropod species, generated custom species-specific repeat libraries with RepeatModeler and masked the genomes with RepeatMasker, to avoid any issues of under- or over masking using other repeat masking programs: a butterfly, Heliconius melpomene [73], a beetle, Tribolium castaneum [74], a millipede, Helicorthomorpha holstii [75], and a scorpion, Centruroides sculpturatus [7,33]. The percentage of total repetitive content for all of these species is presented in Table 4. In general, spiders do have a higher repetitive content than insects, but there is a large range of repetitive content in spiders, compared to which the repetitive content in A. bruennichi is relatively low. All of the selected spider species, aside from Latrodectus hesperus, have higher repetitive content than all other investigated groups, with the exception of ticks and mites, which have very high repetitive content overall (range: 52.6-64.4% repetitive). We conclude from this preliminary investigation that spider genomes, and arachnid genomes generally, do indeed have a higher repeat content than other arthropods."
We hope that this first look, which shows high variability but nonetheless high repeat content in spiders might inspire further research into the variability in repeat content in other groups. We feel that an in depth analysis of the other arthropod groups is not within the scope of this data note but are happy to discuss this topic further.

Reviewer point eight:
On line 158-159 - very cool that the 14th largest scaffold matched the sequence of a recently discovered symbiont of A. bruennichi. Can the authors says if the entire scaffold matched that of the symbiont or was it a mixture of spider and symbiont genetic material? What was the symbiont species, maybe just name the species?

Authors' response:
At this point, we cannot say if the scaffold is a mix or is solely bacterial sequence. Further work by collaborators will look into the bacterial symbiont in detail, including looking into this scaffold – the bacterium is extremely divergent, in 16S sequence space, from known species, thus we cannot say very much about it currently. We moved this information into the "De novo genome assembly" section, as it fits to the discussion of the non-chromosome "lesser" scaffolds.

Reviewer point nine:
Line 165 - for the published RNA-Seq reads used for genome annotation - can the authors say what tissues, sex and developmental stages these reads came from in this paper to give context to the quality of the evidence for the annotation? Perhaps provide the SRA accession for these reads somewhere?

Authors' response:
We have added the information about life stages, as well as the accession numbers, to the text in lines 234-238:
"Raw reads from previously published transcriptome sequencing data of different life stages: 20 pooled eggs (accession number SRR11861505), 20 pooled first instar spiderlings (accession number SRR11861504), one whole body of an adult female (accession number SRR11861502) and one whole body of an adult male (accession number SRR11861503) [5] were mapped against the repeat-masked assembly…"

Reviewer point ten:
The authors say how many genes were predicted from the genome. Maybe I missed it but I could not find the total number of transcripts/proteins predicted from the genome. I think this should also be listed.

Authors' response:
We have added the predicted transcript number (26,318) to the text in lines 248-249.

Reviewer point eleven:
Can the authors be sure to deposit a fasta file of predicted transcripts and proteins from this genome in NCBI and to report the accession for these in the paper itself? In addition the authors could provide these as supplementary files to maximize the utility

of this resource. Can they also provide a link/url in the paper to the UCSC genome browser when it is available?

Authors' response:
The fasta files of predicted transcripts and proteins will be automatically generated by NCBI; our genome is in the final stages of processing, so the files will be available publicly on NCBI when the genome is. However, our files for predicted transcripts and proteins (.aa file for proteins and .codingseq file for transcripts) have been uploaded to gigaDB with the submission of our manuscript, and should thus be accessible – we have added this to the text (lines 254-255):
"The annotation gff3 file and the files containing predicted transcripts and proteins are available on GigaDB."
We have added the URL for the UCSC browser in the section, "Availability of supporting data," although it should now also be publicly searchable on the UCSC genome browser homepage.

Reviewer point twelve:
The authors should also think about if they want to provide their gff file as supplementary , again to maximize utility for the community wanting to understand their annotations.

Authors' response:
Similarly to the point above, the gff3 file is available on GigaDB (line 254-255).

Reviewer point thirteen:
The analyses of the venom and silk genes are very interesting but it is hard to tell what are the number of total venom and silk genes or genome-predicted proteins found or within each category, e.g., how many of each silk gene type or total number of venom genes and the numbers distributed in the islands. This is because (as I interpret it) they report on number of regions on chromosomes where those genes lie, but not the number of genes within those regions. I tried to look further into this by looking at the supplementary blast results, but it is hard to tell because different queries blast to some of the same genomic regions. My point is simply that this information is not easy to find or deduce from the way it's presented.

Authors' response:
Indeed, we struggled with how to present these results, so we appreciate the suggestion about reporting on the number of genes within each island in Figure 3. Originally, we included all matches which passed our filters for E-value and % identity in the supplemental files (thus the confusion with multiple queries blasting to the same region), but have now reduced those matches which map to the same region/gene (lines 322-324 and Supplementary Tables 3-5), and have mentioned in the text and in the figure how many genes we found per silk or venom type on each scaffold (lines 370 -376, 379-381, 390-393).

Reviewer point fourteen:
How well does this assembly perform for the spidroin genes? Are they completely assembled, do they contain Ns, how long are they - what is the length range? This would be another good assessment of the quality of the assembly.

Authors' response:
By manual inspection of the blast hits for spidroin genes in the UCSC genome browser (one can use the "genomebrowser_search" column in Supplementary Table 4 to look at each hit), the assembly in general appears to be high quality with good coverage of long PacBio reads in these areas. However, in some cases, such as for the aciniform spidroin genes, it appears that the annotation may have split the genes into several pieces. In the future, if we or others are interested in the details of the silk genes, manual curation of the annotation could improve the annotation of the silk genes, as the assembly appears complete. As to Ns: many of the spidroin genes contain softmasked repeats, but no Ns.

Reviewer point fifteen:
Great job on an important piece of work!

Powered by Editorial Manager® and ProduXion Manager® from Aries Systems Corporation

Authors' response:
Thank you very much for the detail-oriented, helpful, and positive review of our work. Your suggestions helped us look at the manuscript with fresh eyes, and allowed us to improve our reporting of the findings in this genome in a more explicit and (hopefully) more understandable way.

Authors' reply to the Review Report (Reviewer 2)

We thank Reviewer 2 (and their PhD student) for the input on and subsequent improvement of our manuscript, and have answered their questions here and, where necessary, amended the manuscript to address them. Changes to the manuscript are given here with line numbers and excerpts from the text, and indicated in the text using track changes. Line numbers correspond to the manuscript with "All Markup" showing in track changes.

Reviewer points one and four:
One: The manuscript revolves around the presentation of a high-quality chromosome-level assembly, but the evidence supporting the quality of the assembly is a bit sketchy: merely contiguity statistics, BUSCO scores and a contact map. To be convinced by the quality of the assembly, I would need to see a KAT plot (https://kat.readthedocs.io/en/latest/walkthrough.html#genome-assembly-analysis-using-k-mer-spectra - as the Illumina sequencing depth is only 30X, the authors will probably need to play a bit with the k-mer size parameter to generate a satisfying plot in which the peaks are well separated from one another) and a k-mer completeness estimate.

Four: Running KAT (with default parameters) on the data downloaded from the FTP server provided by the authors yielded a genome size estimate of 1.62 Gb. Also, KAT estimated a k-mer completeness of only 88.9% (for the homozygous peak, which should have a 100% k-mer completeness for a haploid assembly of a diploid genome): this may be due to the 21.5X PacBio coverage used for the assembly being too low for the consensus step to fully correct the sequencing errors, followed by a polishing step with Pilon using an Illumina coverage once again on the lower side (30X). The authors could possibly obtain a better polished assembly with a higher k-mer completeness by performing their polishing using HyPo, which utilizes both PacBio and Illumina data.

Authors' response:
As these two points are related, we have chosen to respond to them together.
We have not seen KAT before and it is indeed a very helpful tool. Thank you for the suggestion. With KAT we now have a much more high-resolution tool to investigate the error profile of the assembly. Given the fact that the pacbio and the Illumina data came from two individuals from different populations, would you still expect a k-mer completeness of close to 100%? We will investigate this and also try HyPo, and if that results in a better kmer match, we will update the submission of the genome on NCBI in the future. However, for the purposes of this data note, we feel that fine-tuning the assembly will not change the downstream results of annotation and genomic architecture, and we hope that you agree. We have mentioned the results of KAT in the manuscript now (lines 192-200), with the explanation that the missing k-mers may be due to sequencing errors remaining in the assembly, or due to the use of individuals from different populations in different years:
"As an additional assessment of assembly quality, we ran the K-mer Analysis Toolkit (KAT v. 2.4.2 , RRID: SCR_016741) [63] comp tool, comparing k-mer content in the Illumina sequencing data to k-mer content in the final assembly. Different values of the parameter k (k =17, 27, 29, 30 and 37) yielded k-mer completeness estimates ranging from 86.55-90.43%. The missing k-mer content in the final assembly may be attributed to the fact that the sequenced individuals came from two different populations, or it may be attributed to errors remaining in the assembly, due to the relatively high error rate and moderate 21.8X coverage of PacBio reads."
Given the mapping rates (see point five below) of the Illumina data for polishing and scaffolding, we think the missing k-mers are more likely due to the use of different individuals, and not due to a high error rate remaining in the assembly.
We generated KAT plots for k values of 17, 27, 29, 30 and 37. Due to memory

limitations of our server (due to simultaneously running the synteny analysis; see point 6 below), we were not able to run higher values of k. According to the KAT documentation, the plot for k = 17 in our case is too low. We do not see many differences in the KAT plots between the higher values of k, although there are differences in completeness and genome size estimates depending on the value of k: k = 17 yields a genome size estimate of 1.23 Gb and completeness of 90.43%; k = 27 yields a genome size estimate of 1.62 Gb and completeness of 88.9%; k = 29 yields a genome size estimate of 1.621 Gb and completeness of 88.96%; k = 30 yields a genome size estimate of 1.763 Gb and completeness of 87.6%; k = 30 yields a genome size estimate of 1.83 Gb and completeness of 86.55%. Given these results, the k value of 29 seems most appropriate, although we have included the range of results in the manuscript.
Regarding polishing: during the genome assembly polishing process we tried PacBio polishing using Racon before running Pilon. However, that did not change the BUSCO results compared to the Pilon only approach, so we are not confident that polishing using PacBio reads with another tool will change the assembly substantially. Therefore, in consideration of the time it would take to compare the assembly and polishing strategies, re-run the scaffolding and annotation, etc., we hope that the assembly in its current state is acceptable, as it remains the most complete spider genome assembly to date.

Reviewer point two:
Also, as the amount of repetitions seems fairly high it would be interesting to see coverage plots (obtained by remapping the PacBio reads on the one hand and the Illumina reads on the other hand on the genome assembly) in order to assess whether some repeated parts have been overcollapsed or (conversely) some haplotypes have not been
properly merged, resulting in artefactual duplications.

Authors' response:
We have included two additional tracks on our UCSC genome browser, which show the Illumina and Pacbio reads mapped onto the assembly. While there certainly seem to be some cases of overcollapsing repeats, it does not seem to be a pervasive problem. We hope that with the addition of this track to the browser, interested readers can look into this in detail.

Reviewer point three:
On line 99 it is mentioned that the genome size was estimated at 1.7 Gb but there is no explanation regarding this estimation: was it obtained using flow cytometry, or by analyzing the k-mer distribution of Illumina reads?

Authors' response:
Thank you for bringing this to our attention. This estimate was based off of the Animal Genome Size Database, which has densitometry data from the very close relatives Argiope trifasciata and Argiope aurantia. We have added this rationalization to the text. We previously rounded up to 1.7 to not overestimate our coverage estimate if A. bruennichi has a slightly larger genome size than its close relatives. However, we have now added additional investigations into the expected genome size using bioinformatic methods (backmap.pl), which taken together with the data for the other species show 1.675Gb as an appropriate estimate; therefore, we have changed our coverage measurement slightly to reflect this.
We have added all of this information into a new section, "Genome size estimation and coverage" (lines 157-169):
"We estimated the genome size of Argiope bruennichi based on data for closely related species, and bioinformatically based on previously published Illumina paired-end data derived from a single female individual from a population in Madeira (SRA accession number: ERX533198) [5], which we later used for polishing the assembly.
The closely related species A. aurantia and A. trifasciata have genome size estimates based on densitometry data of 1.620 gigabasepairs (Gb) [45] or 1.650 Gb [46] for A. aurantia and 1.690 Gb for A. trifasciata [45,47]. Using the backmap.pl (v. 0.3) pipeline [48–55] on the Illumina data from A. bruennichi [5], we generated a genome size estimate of 1.740 Gb. Averaging these four genome size measurements yields an estimate of 1.675 Gb.
Given this estimate, the PacBio sequencing yielded 21.8X coverage (approximately

36.65 Gb sequenced, with an estimated genome size of 1.675 Gb)"
We also tried using KAT to estimate the genome size, but found that it was extremely sensitive to the chosen value of k, and thus left it out of the estimate. The KAT documentation describes k values from 17 to 63 as generally reasonable. Therefore, we ran KAT comp with k values 17, 27, 29, 30 and 37. Higher values required more memory than we have on our server. With increasing values of k we had increasing genome size estimates (1.23 Gb, 1.62 Gb, 1.621 Gb, 1.763 Gb, 1.83 Gb).

Reviewer point five:
As the PacBio, Illumina and Hi-C data were generated from different individuals collected several years apart, the mapping rates of the Illumina data on the initial PacBio assembly as well as the mapping rate of the the Hi-C data on the polished assembly should be mentioned.

Authors' response:
We have included information on the mapping rates in lines 175-176 and lines 183-184:
"Mapping for the three rounds of polishing resulted in a mapping rate ranging from 92.55-93.69%."
"The sequences from this [the Hi-C] library had a 94.71% mapping rate against the polished assembly."

Reviewer point six:
The part entitled "whole-genome duplication" does not really look into WGS per se but rather only analyzes the duplication of the Hox gene cluster, which could also result from a segmental duplication involving this cluster. Argiope bruennichi being the first chromosome-scale assembly made available for any arachnid, the authors should seize this opportunity to perform a synteny and/or microsynteny analysis at chromosome level in order to check whether they find evidence supporting an actual whole-genome duplication.

Authors' response:
This suggestion is indeed very interesting. We tried to run Satsuma Synteny 2 on our genome, comparing the whole genome to itself, which proved to be too computationally intensive for our server. Therefore, we started by comparing the two hox-containing chromosomes, which do indeed appear to be very orthologous (Screenshot of MizBee viewer available upon request), suggesting at least duplication of these complete chromosomes, if not the whole genome.
To look for further evidence, we tried running a single chromosome against the whole genome to find the duplicate of that chromosome. This analysis ran for more than three weeks before it used all of the memory of our server and "core dumped". We then considered going pairwise, comparing one chromosome to another through all possible comparisons, however the comparison of the two Hox-containing scaffolds above took more than 5 days and all of our server's memory to run. The pairwise analysis of all remaining chromosomes, if they took the same time, would have taken more time than we were allotted for the revision, and would tie up all of our computational resources for other projects during that time. If the reviewer has a suggestion for another tool for synteny analysis (perhaps something lighter weight in terms of computation), we would welcome it, as we find this very interesting in general.
Because we are unable to provide evidence of WGD, and only of the two Hox-containing chromosomes, we have changed our manuscript to report on "Hox cluster duplication" (lines 54, 328, 356-361, 449, 725-727) instead of whole-genome duplication:
"It is possible that Hox Cluster B in spiders has changed or lost functionality following the proposed ancestral WGD event. The presence of two Hox clusters in our assembly is suggestive, but not evidence, of WGD in A. bruennichi, as it could have also arisen from duplication of only the Hox-containing chromosome; future studies can capitalize on the now-available chromosome-level assemblies for several groups (e.g. horseshoe crabs, ticks, and our spider) [72,88] to do more detailed analyses of duplication across chelicerates."
We feel that with the publication of this genome, as well as the chromosome-level genomes of the horseshoe crab and a handful of tick species which have been published since we submitted our manuscript, the stage is set for a chelicerate-wide analysis of WGD. This is not possible for us given the computational resources

needed, but we look forward to sharing our data with others so that it can be used for this purpose. Indeed, this is why we chose the format of a data note for fast dissemination of our data.

Reviewer point seven:
-line 38: "Arachnids" should be spelled "arachnids

Authors’ response:
Thank you for bringing this to our attention; it has been fixed in the text (line 49).

Reviewer point eight:
- lines 38-39: "whole-genome duplication" is normally with a hyphen

Authors’ response:
Thank you for bringing this to our attention. We have fixed this in the indicated lines in the abstract, and throughout the text (lines 50, 93, 303, 329, 433, 726).

Reviewer point nine:
- lines 53 and 230: I checked reference 7 and it does not really seem to support the assertion that "chromosome-level genome assembly would greatly increase the potential for inference on evolutionary adaptation and modes of speciation" (there is no discussion about the need for a chromosome-level genome assembly in that paper)

Authors’ response:
This reference (formerly reference 7, now reference 8) shows how chromosomal rearrangements play a role in speciation in spiders. While the authors do not directly make the argument that one needs a genome assembly for this purpose, it nonetheless supports the idea that information on chromosomal conformation (in this case assessed at a karyotype level) is important to understand speciation and adaptation. Chromosomal genome assemblies are another tool to complement and expand our understanding of the role of chromosomal rearrangements in speciation. Since we realized that the argumentation in the introduction could be stronger, clearer, and better supported by references to the literature, we have rewritten these paragraphs (lines 63-90) to provide a stronger argument, and added more references that rely on genomic sequence data, but have left this reference in due to its relevance for the taxonomic group:
“With regards to adaptation, work on cobweb spiders (Theridiidae) has revealed a whole-genome duplication that may facilitate diversification [7], with other studies highlighting a key role of tandem duplication and neofunctionalization of genes in the diversification and specialization of spider silks [8] and venoms [9]. A key aspect that has been missing from studies to date is the role of genome organization in facilitating or impeding adaptation as there have been no studies to date on spiders that have provided a chromosomal framework for the genome.
Understanding the chromosomal organization of a genome is critical for identification of processes underlying divergence between populations, adaptation, and speciation. Indeed, the potential role of chromosomal reorganization in species formation has long been the subject of debate, in particular in Drosophila species where polytene chromosomes allowed early visualization of chromosomal rearrangements [10]. Among spiders, karyotype data are still used to identify changes in chromosomes associated with speciation [11]. With the advent of detailed genomic data, there has been renewed focus on the role that structural variants in the genome can play as drivers of adaptation and speciation, associated with translocations, fusions, and inversions [12], as well as with admixture and associated demographic changes [13]. Recent data from sister species of the genus Drosophila suggest that the establishment of inversion polymorphisms within isolated and/or heterogeneous environments may well set the stage for species formation [14]. In order to develop a broader understanding of the role of structural variation in adaptation and speciation [15–22], we need chromosome-level genomes that provide the ability to map the order of genes, define chromosomal gene neighborhoods, and identify potential genomic islands of differentiation [23–26].”

Reviewer point ten:
- line 305: I could not find the "Wasp spider hub" on the UCSC genome browser, please provide a direct link

	Authors' response:<br>Our apologies for this, as public listing of the hub was delayed; the hub is now publicly listed at the UCSC Genome browser, and we have added the direct link to the accessibility of data section (lines 416-417).<br><br>Reviewer point eleven:<br>- figure 3b could be removed as it does not bring much relevant information that is not already present in the text.<br><br>Authors' response:<br>While we understand the reviewer's point that figure 3B could be seen as redundant relative to the text, we see the figure as a complement to more easily follow the text and place the results for the different gene groups into context with one another. If it is acceptable to the reviewers and editors, we would like to leave figure 3B in.
**Additional Information:**	
**Question**	**Response**
Are you submitting this manuscript to a special series or article collection?	No
**Experimental design and statistics**<br><br>Full details of the experimental design and statistical methods used should be given in the Methods section, as detailed in our Minimum Standards Reporting Checklist. Information essential to interpreting the data presented should be made available in the figure legends.<br><br>Have you included all the information requested in your manuscript?	Yes
**Resources**<br><br>A description of all resources used, including antibodies, cell lines, animals and software tools, with enough information to allow them to be uniquely identified, should be included in the Methods section. Authors are strongly encouraged to cite Research Resource Identifiers (RRIDs) for antibodies, model organisms and tools, where possible.<br><br>Have you included the information requested as detailed in our Minimum Standards Reporting Checklist?	Yes
**Availability of data and materials**	Yes

Powered by Editorial Manager® and ProduXion Manager® from Aries Systems Corporation

All datasets and code on which the conclusions of the paper rely must be either included in your submission or deposited in publicly available repositories (where available and ethically appropriate), referencing such data using a unique identifier in the references and in the “Availability of Data and Materials” section of your manuscript.<br><br>Have you have met the above requirement as detailed in our Minimum Standards Reporting Checklist?	

Powered by Editorial Manager® and ProduXion Manager® from Aries Systems Corporation

Manuscript

Click here to access/download;Manuscript;ArgiopeGenome-Manuscript-revisions_final.docx

Title

Chromosome-level reference genome of the European wasp spider *Argiope bruennichi*: a resource for studies on range expansion and evolutionary adaptation

Authors

Monica M. Sheffer[1†], Anica Hoppe[2,3], Henrik Krehenwinkel[4], Gabriele Uhl[1], Andreas W. Kuss[5], Lars Jensen[5], Corinna Jensen[5], Rosemary G. Gillespie[6], Katharina J. Hoff[2,3*] & Stefan Prost[7,8*]

†indicates corresponding author

*indicates equal contribution

Affiliations

[1] Zoological Institute and Museum, University of Greifswald, Greifswald, Germany

[2] Institute of Mathematics and Computer Science, University of Greifswald, Greifswald, Germany

[3] Center for Functional Genomics of Microbes, University of Greifswald, Greifswald, Germany

[4] Department of Biogeography, University of Trier, Trier, Germany

[5] Interfaculty Institute for Genetics and Functional Genomics, University of Greifswald, Greifswald, Germany

[6] Department of Environmental Science Policy and Management, University of California Berkeley, Berkeley, -USA

[7] LOEWE-Centre for Translational Biodiversity Genomics, Senckenberg, Frankfurt Germany

[8] South African National Biodiversity Institute, National Zoological Gardens of South Africa, Pretoria, South Africa

E-Mails and ORCID

Name	E-Mail Address	ORCID
Monica M. Sheffer	monica.sheffer@uni-greifswald.de	0000-0002-6527-4198
Anica Hoppe	anica.hoppe@stud.uni-greifswald.de	0000-0002-2586-1654
Henrik Krehenwinkel	krehenwinkel@uni-trier.de	0000-0001-5069-8601
Gabriele Uhl	gabriele.uhl@uni-greifswald.de	0000-0001-8758-7913
Andreas W. Kuss	andreas.kuss@uni-greifswald.de	0000-0002-9401-4627
Lars Jensen	larsriff.jensen@uni-greifswald.de	
Corinna Jensen	corinna.jensen@uni-greifswald.de	
Rosemary G. Gillespie	gillespie@berkeley.edu	0000-0003-0086-7424
Katharina J. Hoff	katharina.hoff@uni-greifswald.de	0000-0002-7333-8390
Stefan Prost	stefanprost.research@protonmail.com	0000-0002-6229-3596

Abstract

Background: *Argiope bruennichi*, the European wasp spider, has been investigated intensively as ~~to~~a focal species for studies on sexual selection, chemical communication, and the dynamics of rapid range expansion at a behavioral and genetic level. However, the lack of a reference genome has limited insights into the genetic basis for these phenomena. Therefore, we assembled a high-quality chromosome-level reference genome of the European wasp spider as a tool for more in-depth future studies.

Findings: We generated, *de novo*, a 1.67Gb genome assembly of *A. bruennichi* using 21.5X PacBio sequencing, polished with 30X Illumina paired-end sequencing data, and proximity ligation (Hi-C) based scaffolding. This resulted in an N50 scaffold size of 124Mb and an N50 contig size of 288kb. We found 98.4% of the genome to be contained in 13 scaffolds, fitting the expected number of chromosomes (n = 13). Analyses showed the presence of 91.1% of complete arthropod BUSCOs, indicating a high quality ~~of the~~ assembly.

Conclusions: We present the first chromosome-level genome assembly in the ~~class Arachnida~~order Araneae. With this genomic resource, we open the door for more precise and informative studies on evolution and adaptation not only in *A. bruennichi*, ~~as well as on several interesting topics~~but also in ~~Arachnids~~arachnids overall, shedding light on questions such as the genomic architecture of traits, whole~~-~~-genome duplication and the genomic mechanisms behind silk and venom evolution.

Keywords

Argiope bruennichi, genome assembly, Araneae, spider, PacBio, Hi-C, chromosome-level, ~~whole genome~~Hox duplication, silk, venom

Data description

Context

Spider genomes are of great interest, for instance in the context of silk and venom evolution and biomedical and technical applications. Additionally, spiders are fascinating from ecological and evolutionary perspectives. As the most important predators of terrestrial arthropods, they play a key role in terrestrial food webs [1–4]. Spiders are distributed on every continent~~,~~ except Antarctica, and diverse habitats can be occupied by single species or multiple close relatives [5,6], making them ideal for studies on environmental plasticity, adaptation and speciation. With regards to adaptation, work on cobweb spiders (Theridiidae) has revealed a whole-genome duplication that may facilitate diversification [7], with other studies highlighting a key role of tandem duplication and neofunctionalization of genes in the diversification and specialization of spider silks [8] and venoms [9]. A key aspect that has been missing from studies to date is the role of genome organization in facilitating or impeding adaptation as there have been no studies to date on spiders that have provided a chromosomal framework for the genome.

Understanding the chromosomal organization of a genome is critical for identification of processes underlying divergence between populations, adaptation, and speciation. Indeed, the potential role of chromosomal reorganization in species formation has long been the subject of debate, in particular in *Drosophila* species where polytene chromosomes allowed early visualization of chromosomal rearrangements [10]. Among

spiders, karyotype data are still used to identify changes in chromosomes associated with speciation [11]. With the advent of detailed genomic data, there has been renewed focus on the role that structural variants in the genome can play as drivers of adaptation and speciation, associated with translocations, fusions, and inversions [12], as well as with admixture and associated demographic changes [13]. Recent data from sister species of the genus *Drosophila* suggest that the establishment of inversion polymorphisms within isolated and/or heterogeneous environments may well set the stage for species formation [14]. In order to develop a broader understanding of the role of structural variation in adaptation and speciation [15–22], we need chromosome-level genomes that provide the ability to map the order of genes, define chromosomal gene neighborhoods, and identify potential genomic islands of differentiation [23–26]. ~~A chromosome-level genome assembly would greatly increase the potential for inference on evolutionary adaptation and modes of speciation [8]. For instance, a well-resolved genome is critical, if evolutionary adaptation happens along genomic islands of differentiation [13–16] or to assess the importance of large genomic rearrangements, such as inversions, in speciation [17–23].~~

To the best of our knowledge, ~~only eight~~ten draft spider genomes have been published to date [7,27–33], most of which focus on silk and venom genes, while one discusses whole-genome duplication [7] and the publication of the most recent two focuses on gene content evolution across arthropods [33]. There ~~are three~~is one additional, as yet unpublished, spider genome assembly~~ies~~ available on NCBI (National Center for Biotechnology Information) (*Anelosimus studiosus*, accession number~~s: *Anelosimus studiosus*:~~ GCA_008297655.1~~; *Latrodectus hesperus*: GCA_000697925.2; *Loxosceles*~~

~~*reclusa*: GCA_001188405.1~~). Spider genomes are considered notoriously difficult to sequence, assemble, and annotate for a number of factors, including their relatively high repeat content, low guanine cytosine (GC) content~~-~~, high levels of heterozygosity in the wild [27] and due to the fact that they possess some extremely long coding genes in the spidroin gene families [28,29,34,35]. Due to these challenges, the completeness of the available spider genomes varies greatly between assemblies (Supplementary Table 1). All of them are incomplete and there is no chromosome-level assembly published for any spider to date. While this does not lessen the conclusions of the above-mentioned studies, a chromosome-level assembly would open doors for more detailed studies on the genomic architecture of gene families, such as silk and venom genes, providing greater understanding of the evolutionary mechanisms driving the diversification of these gene families and genome evolution, in addition to the aforementioned applications in understanding adaptation and speciation.

The European wasp spider, *Argiope bruennichi* (Scopoli, 1772), is an orb-weaving spider in the family Araneidae (Figure 1). Despite the lack of a reference genome, *A. bruennichi* has been the focal species for studies on local adaptation, range expansion, admixture, and biogeography [5,36–38]. These studies have suggested that the range expansion and subsequent local adaptation of *A. bruennichi* from southern to northern Europe was caused by genetic admixture. However, it is not yet known which regions of the genome are admixed, and if these regions are truly responsible for adaptation to colder climates. *A. bruennichi* has also been well studied in the context of dispersal and life history traits [39], as well as sexual selection and chemical communication (e.g. [40–44]). A high-quality reference genome would allow altogether new insights into our understanding of

the genetic basis of these phenomena. Considering this background, a chromosome-level reference genome would be highly desirable for the species.

Sampling, DNA extraction and sequencing

Adult female *Argiope bruennichi* individuals were collected in the south of Portugal in 2013 and 2019 (Latitude: 37.739 N, Longitude: -7.853 E). As inbred lines of the species do not exist, we selected a population which was previously found to have low heterozygosity in the wild, likely due to naturally high levels of inbreeding [5].

For the baseline assembly, deoxyribonucleic acid (DNA) was extracted from a female collected in 2013 using the ArchivePure blood and tissue kit (5 PRIME, Hamburg, Germany), according to the manufacturer's protocol. A ribonucleic acid (RNA) digestion step was included using RNAse A solution (7000 U mL^{-1}; 5 PRIME). The DNA was stored at -80°C until library preparation in 2017. The DNA extract was cleaned using a salt:PCI cleaning step, and had a fragment size distribution from 1,300-165,500 bp (peak at 14,002 bp) before size selection. The library was size selected to 15 kilobasepairs (kb) using Pippin prep and subsequently sequenced in ~~2017~~ 2018 at the QB3 Genomics facility at the University of California Berkeley on a Pacific Biosciences Sequel I platform (PacBio, Menlo Park, CA, USA) on 10 cells. ~~The sequencing yielded 21.5X coverage (approximately 36.65 gigabasepairs (Gb), with an estimated genome size of 1.7 Gb.~~

The specimen collected in 2019 was used to build a proximity-ligation based short-read library ("Hi-C"). Four Hi-C libraries were prepared from a single individual using Dovetail™ Hi-C library preparation kit according to the manufacturer's protocol (Dovetail Genomics, Santa Cruz, CA). The specimen was anesthetized with CO_2 before preparation. In brief, the legs were removed from the body and stored in liquid nitrogen, and the leg tissue was

disrupted in liquid nitrogen using a mortar and pestle. Chromatin was fixed with formaldehyde, then extracted. Fixed chromatin was digested with DpnII, the 5’ overhangs filled in with biotinylated nucleotides, and the free blunt ends were ligated. After ligation, crosslinks were reversed and the DNA purified to remove proteins. Purified DNA was treated to remove biotin that was not internal to ligated fragments. The DNA was then sheared to ~350 bp mean fragment size using a Covaris S2 Focused-ultrasonicator. A typical Illumina library preparation protocol followed, with end repair and Illumina adapter ligation. Biotinylated fragments were captured with streptavidin beads before PCR (polymerase chain reaction) amplification (12 cycles), and size selection was performed using SPRI-select beads (Beckman Coulter GmbH, Germany) for a final library size distribution centered around 450 bp. The library was sequenced to approximately 440 million paired end reads on one Flowcell of an Illumina NextSeq 550 with a High Output v2 kit (150 cycles).

Genome size estimation and coverage

We estimated the genome size of *Argiope bruennichi* based on data for closely related species, and bioinformatically based on previously published Illumina paired-end data derived from a single female individual from a population in Madeira (SRA accession number: ERX533198) [5], which we later used for polishing the assembly.

The closely related species *A. aurantia* and *A. trifasciata* have genome size estimates based on densitometry data of 1.620 gigabasepairs (Gb) [45] or 1.650 Gb [46] for *A. aurantia* and 1.690 Gb for *A. trifasciata* [45,47]. Using the backmap.pl (v. 0.3) pipeline [48–55] on the Illumina data from *A. bruennichi* [5], we generated a genome size estimate

of 1.740 Gb. Averaging these four genome size measurements yields an estimate of 1.675 Gb.

Given this estimate, the PacBio sequencing yielded 21.8X coverage (approximately 36.65 Gb sequenced, with an estimated genome size of 1.675 Gb).

De novo genome assembly

First, we generated a baseline assembly using 21.85X long-read Pacific Biosciences (PacBio) Sequel I sequencing data and the wtdbg2 assembler (v. 2.3) (WTDBG, RRID:SCR_017225) [56]. Next, we polished the assembly by applying three rounds of Pilon (v. 1.23) (Pilon, RRID:SCR_014731) [57] using the ~30X of previously published Illumina paired-end data [5]. Mapping for the three rounds of polishing resulted in a mapping rate ranging from 92.55-93.69%.This The polishing resulted in 13,843 contigs with an N50 of 288.4 kilobase pairs (kb), and an overall assembly size of 1.67 gigabase pairs (Gb). Analysis of Benchmarking Universal Single Copy Orthologs (BUSCO) (v. 3.1.0) scores, using the arthropod data set (BUSCO, RRID:SCR_015008) [58], showed the presence of 90.2% of complete BUSCOs, with 86.4% complete and single-copy BUSCOs, 3.8% complete and duplicated BUSCOs, 3.3% fragmented BUSCOs, and 6.5% missing BUSCOs (Table 1). Next, we scaffolded the contigs using a proximity-ligation based short-read library [59]. The sequences from this library had a 94.71% mapping rate against the polished assembly. Scaffolding using HiRise v. 2.1.7, a software pipeline designed specifically for using proximity ligation data to scaffold genome assemblies [59], resulted in 13 12 scaffolds over 1 megabase pairs (Mb) in size and one scaffold just under 1 Mb in size. ,These 13 scaffolds comprising comprise 98.4% of the assembly, with a genome assembly scaffold N50 of 124Mb and BUSCO scores of 91.1% complete genes

(Figure 2, Table 1). Genome assembly statistics were calculated using QUAST v. 5.0.2 (QUAST, RRID:SCR_001228) [60] applying default parameters, except --min-contig 0. Previous studies have inferred the chromosome number of *A. bruennichi* to be 13, indicating our genome assembly is full-chromosome level [61,62]. As an additional assessment of assembly quality, we ran the K-mer Analysis Toolkit (KAT v. 2.4.2 , RRID: SCR_016741) [63] *comp* tool, comparing k-mer content in the Illumina sequencing data to k-mer content in the final assembly. Different values of the parameter *k* (*k* =17, 27, 29, 30 and 37) yielded k-mer completeness estimates ranging from 86.55-90.43%. The missing k-mer content in the final assembly may be attributed to the fact that the sequenced individuals came from two different populations, or it may be attributed to errors remaining in the assembly, due to the relatively high error rate and moderate 21.8X coverage of PacBio reads.

The 13 largest scaffolds are ~~thus~~ henceforth referred to as Chromosomes 1-13, ordered according to size (Figure 2~~C~~B). The 14th-largest scaffold (Scaffold 839) contained the 16S sequence of a recently discovered, as yet unnamed, bacterial symbiont of *A. bruennichi* [48]. The remaining 2,217 scaffolds are much smaller, ranging from 1,747-258,743 bp in length (Supplementary Figure 1) and will henceforth be referred to as "lesser scaffolds".

Table 1: *Argiope bruennichi* genome assembly completeness

Genome assembly statistic	Unscaffolded	Scaffolded
**Assembly size**	1,669,116,561	1,670,285,661
**AT [a] / GC / N content (%)**	70.7 / 29.3 / 0	70.6 / 29.3 / 0.1
**Number of contigs / scaffolds**	13,843	2,231
**Longest contig / scaffold**	2,039,454	143,171,375

**Contig / scaffold N50**	288,395	124,235,998
**Contig / scaffold N90**	67,231	119,022,586
**% repetitive**	34.66	34.64
**BUSCO analysis** [b]		
Complete BUSCOs (%)	90.2	91.1
Complete and single-copy BUSCOs (%)	86.4	87.8
Complete and duplicated BUSCOs (%)	3.8	3.3
Fragmented BUSCOs (%)	3.3	2.8
Missing BUSCOs (%)	6.5	6.1

Genome assembly statistics were calculated using QUAST v. 5.0.2 (QUAST, RRID:SCR_001228) [60] using default parameters, except --min-contig 0.

[a] AT: adenine thymine

[b] BUSCO analysis using default parameters against the arthropod dataset

Repeat masking and removal of contaminants

The assembly was repeat-masked using a combination of the *de novo* repeat finder RepeatModeler (v. open-1.0.11) (RepeatModeler, RRID:SCR_015027) [64] and the homology-based repeat finder RepeatMasker (v. open-4.0.9) (RepeatMasker, RRID:SCR_012954) [65]. Repetitive regions accounted for 34.64% of the genome assembly, of which the majority (20.52% of the genome) consisted of unclassified repeats, meaning that they have not been classified in previous studies. The remaining repetitive elements were made up of DNA elements (i.e. transposable elements: 6.27%), long interspersed nuclear elements (LINEs: 1.60%), simple repeats (i.e. duplications of 1-5 bp: 1.58%), long terminal repeat (LTR) elements (0.76%), satellites (0.63%), low complexity repeats (i.e. poly-purine or poly-pyrimidine stretches: 0.42%), and short interspersed nuclear elements (SINEs: 0.08%) (Table 2). BlobTools (v. 1.0) (Blobtools, RRID:SCR_017618) [66] was used to search for contamination of bacterial or

mitochondrial sequences, finding none. ~~and subsequently mitochondrial sequences and bacterial scaffolds were removed from the assembly. The 14th-largest scaffold (Scaffold 839) matched the sequence of a recently-discovered bacterial symbiont of *Argiope bruennichi* [48].~~

Table 2: *Argiope bruennichi* repetitive DNA elements

Type of element	Number of elements	Length (bp)	Percentage of assembly
**SINEs**	4,643	1,314,740	0.08 %
**LINEs**	52,648	26,768,096	1.60 %
**LTR elements**	21,649	12,683,330	0.76 %
**DNA elements**	282,019	104,785,665	6.27 %
**Unclassified**	1,359,138	342,727,030	20.52 %
**Small RNA**	0	0	0.00 %
**Satellites**	28,474	10,495,658	0.63 %
**Simple repeats**	595,962	26,379,486	1.58 %
**Low complexity**	137,182	6,952,634	0.42 %
		**Total:**	34.64 %

Repetitive elements were classified using RepeatModeler (v. open-1.0.11) (Smit & Hubley, 2008) and RepeatMasker (v. open-4.0.9) (Smit & Hubley, 2013).

Genome annotation

Raw reads from previously published transcriptome sequencing data of different life stages: 20 pooled eggs (accession number SRR11861505), 20 pooled first instar spiderlings (accession number SRR11861504), one whole body of an adult female (accession number SRR11861502) and one whole body of an adult male (accession number SRR11861503) [5] were mapped against the repeat-masked assembly using HISAT2 (v. 2.1.0) (HISAT2, RRID:SCR_015530) [67]. After conversion of the resulting SAM file into a BAM file and subsequent sorting using SAMtools (v. 1.7) (SAMTOOLS,

RRID:SCR_002105) [49], the sorted BAM file was converted to intron-hints for AUGUSTUS (v. 3.3.2) (Augustus, RRID:SCR_008417) [68] using AUGUSTUS scripts. AUGUSTUS was run on the soft-masked genome with the *Parasteatoda* parameter set. The resulting gff file containing predicted genes was converted into a gtf file using the AUGUSTUS script gtf2gff.pl. Additional AUGUSTUS scripts (getAnnoFastaFromJoinGenes.py and fix_in_frame_stop_codon_genes.py) were used to find and replace predicted protein coding genes containing in-frame stop codons with newly predicted genes. The resulting gtf file containing 23,270 predicted genes (26,318 transcripts) was converted to gff3 format using gtf2gff.pl and protein sequences of predicted genes were extracted with getAnnoFastaFromJoinGenes.py. Finally, functional annotation was performed using InterProScan (v. 5.39-77.0) (InterProScan, RRID:SCR_005829) [69,70] (Table 3). The majority of annotated genes fall on the 13 chromosome scaffolds, although 272 transcripts were predicted on the lesser scaffolds. The annotation gff3 file and the files containing predicted transcripts and proteins are available on GigaDB.

Table 3: *Argiope bruennichi* genome annotation statistics

**Genome Annotation Statistic**	**Value**
**Number of protein coding genes**	23,270
**Functionally annotated genes (%)**	81.0
**Average exon length (bp)**	200
**Average intron length (bp)**	4,035
**BUSCO analysis** [a]	
Complete BUSCOs (%)	89.3
Complete and single-copy BUSCOs (%)	76.7
Complete and duplicated BUSCOs (%)	12.6
Fragmented BUSCOs (%)	7.0
Missing BUSCOs (%)	3.7

[a] BUSCO analysis using default parameters against the arthropod dataset

Comparative genomic analysis of repeat content

High repetitiveness is characteristic of spider genomes [27]. In order to compare the repeat content of *A. bruennichi* with that of other spiders, we downloaded the genome assemblies of several other spider species from NCBI and the DNA Data Bank of Japan (DDBJ) (accession numbers in Table 4), then treated them in the same manner as the *A. bruennichi* genome, masking the repeats using RepeatModeler (v. open-1.0.11) [64] and RepeatMasker (v. open-4.0.9) [65]. *Acanthoscurria geniculata* was excluded from this analysis due to the ~~very large and~~ relatively poorly assembled genome. The *A. bruennichi* genome has a slightly lower percentage of repetitive element content (34.64%) compared to most other spiders (Table 4). Some species, such as *Loxosceles reclusa, Trichonephila clavipes* (formerly *Nephila clavipes*), *Anelosimus studiosus* and *Parasteatoda tepidariorum,* have similar repetitive content (36.51%, 36.61%, 35.98% and 36.79% respectively); other species have much higher repetitive content, such as *Araneus ventricosus, Dysdera silvatica, Stegodyphus dumicola, Stegodyphus mimosarum* and *Pardosa pseudoannulata* (55.96%, 60.03%, 58.98%, 56.91% and 48.61% respectively). Only *Latrodectus hesperus* has lower repetitive content (20.97%). The classification and relative percentage of these repeats can be found in Supplementary Table 2 and Supplementary Figure ~~1~~2. It is often asserted that the repeat content in spiders is higher in general than in other arthropod groups [i.e. 27]. In order to test this assertion, we looked into the repeat content in genomes of additional arthropod species. We obtained repeat content estimates, for which the repeats were masked using RepeatModeler and

RepeatMasker, for three insect species (*Bombus terrestris, Drosophila melanogaster* and *Rhodnius prolixus* [71]), and seven tick and mite species (*Ixodes persulcatus, Haemaphysalis longicornis, Dermacentor silvarum, Hyalomma asiaticum, Rhipicephalus sanguineus, and Ixodes scapularus* [72]). We additionally downloaded the genomes of four more arthropod species, generated custom species-specific repeat libraries with RepeatModeler and masked the genomes with RepeatMasker, to avoid any issues of under- or over masking using other repeat masking programs: a butterfly, *Heliconius melpomene* [73], a beetle, *Tribolium castaneum* [74], a millipede, *Helicorthomorpha holstii* [75], and a scorpion, *Centruroides sculpturatus* [7,33]. The percentage of total repetitive content for all of these species is presented in Table 4. In general, spiders do have a higher repetitive content than insects, but there is a large range of repetitive content in spiders, compared to which the repetitive content in *A. bruennichi* is relatively low. All of the selected spider species, aside from *Latrodectus hesperus,* have higher repetitive content than all other investigated groups, with the exception of ticks and mites, which have very high repetitive content overall (range: 52.6-64.4% repetitive). We conclude from this preliminary investigation that spider genomes, and arachnid genomes generally, do indeed have a higher repeat content than other arthropods.

Table 4: Total repetitive content in the genomes of spiders and selected other arthropods

Class	Order	Species	% repetitive	Accession number [reference]
Arachnida	Araneae	*Argiope bruennichi*	34.64	--
		Araneus ventricosus	55.96	BGPR01000001-BGPR01300721 [a] [29]
		Trichonephila clavipes	36.61	GCA_002102615.1 [b] [28]
		Dysdera silvatica	60.03	GCA_006491805.1 [b] [32]
		Stegodyphus dumicola	58.98	GCA_010614865.1 [b] [31]
		Stegodyphus mimosarum	56.91	GCA_000611955.2 [b] [27]
		Pardosa pseudoannulata	48.61	GCA_008065355.1 [b] [30]

		Loxosceles reclusa	36.51	GCA_001188405.1 [b] [33]~~[unpublished]~~
		Anelosimus studiosus	35.98	GCA_008297655.1 [b] [unpublished]
		Latrodectus hesperus	20.97	GCA_000697925.2 [b] [33]~~[unpublished]~~
		Parasteatoda tepidariorum	36.79	GCA_000365465.3 [b] [7]
	Scorpiones	*Centruroides sculpturatus*	34.4	GCA_000671375.2 [b] [26,31]
	Acari	*Ixodes persulcatus*	64.4	GCA_013358835.1 [b] [72]
		Haemaphysalis longicornis	59.3	GCA_013339765.1 [b] [72]
		Dermacentor silvarum	60.2	GCA_013339745.1 [b] [72]
		Hyalomma asiaticum	52.6	GCA_013339685.1 [b] [72]
		Rhipicephalus sanguineus	61.6	GCA_013339695.1 [b] [72]
		Rhipicephalus microplus	63.1	GCA_013339725.1 [b] [72]
		Ixodes scapularis	63.5	GCF_002892825.2 [b] [72,76]
Diplopoda	Helminthomorpha	*Helicorthomorpha holstii*	23.5	GCA_013389785.1 [b] [75]
Insecta	Hemiptera	*Rhodnius prolixus*	29.25	GCA_000181055.3 [b] [71]
	Hymenoptera	*Bombus terrestris*	12.51	GCA_000214255.1 [b] [71]
	Coleoptera	*Tribolium castaneum*	28.5	GCA_000002335.3 [b] [74]
	Lepidoptera	*Heliconius melpomene*	32.4	GCA_000313835.2 [b] [73]
	Diptera	*Drosophila melanogaster*	19.31	GCA_000001215.4 [b] [71]

Repetitive elements were classified using RepeatModeler (v. open-1.0.11) [64]~~(Smit & Hubley, 2008)~~ and RepeatMasker (v. open-4.0.9) [65]~~(Smit & Hubley, 2013)~~.

[a] DNA Data Bank of Japan (DDBJ)

[b] GenBank, National Center for Biotechnology Information (NCBI)

Genome architecture of Hox, spidroin and venom genes

Previous studies on spider genomes have focused on whole-genome duplication, silk gene evolution, and venom gene evolution [7,27–30]. Therefore, to place the *A. bruennichi* genome into the same context, we manually curated three gene sets from publicly available protein sequences: Hox, spidroin (silk), and venom genes. Because Hox genes are highly conserved across taxa [77], we chose the most complete sequences for the ten arthropod Hox gene classes from spiders without regard to the

relatedness of the species to *A. bruennichi* (Supplementary ~~Table 3~~File 1). In contrast to Hox genes, spidroin and venom genes are highly polymorphic and species-specific [78–81]. For the spidroin gene set, we downloaded protein sequences of the seven spidroin gene classes exclusively from five species of the genus *Argiope* (Supplementary ~~Table 4~~File 2). Venom genes are best studied in spiders that are medically significant to humans, which are very distant relatives to *A. bruennichi* [51–54]. To allow comparison, we focused on venom gene sequences available for araneid spiders (two species, Supplementary ~~Table 5~~File 3); however, the function and classification of these genes is poorly understood. With these three gene sets (Hox, spidroin, and venom), we performed a TBLASTN search against our genome assembly (v. 2.10.0+) (TBLASTN, RRID:SCR_011822) [86,87]. We recorded the genomic position of the best matches and compared them with the AUGUSTUS gene predictions for those locations. We employed a conservative E-value cutoff of less than 1.00~~E~~ x 10^{-20} and only included results with an identity greater than 60%. If hits overlapped on a scaffold or mapped to the same gene, only the hit with the highest identity and lowest E-value was retained. In cases where these metrics conflicted, the hit with the longest match length was retained. The manually curated FASTA files of each gene set used for the TBLASTN search are available in Supplementary Files 1-3. A table of the best matches with accession numbers for each gene set is available in Supplementary Tables 3-5.

~~Whole genome~~Hox cluster duplication

In 2017, Schwager *et al.* revealed that a ~~whole~~ whole-genome duplication (WGD) event has occurred in the ancestor of scorpions and spiders, as evidenced by a high number of duplicated genes, including two clusters of Hox genes in *Parasteatoda tepidariorum* and

Formatted: Superscript

the bark scorpion *Centruroides sculpturatus* [7]. In their study, they found one nearly-complete cluster of Hox genes on a single scaffold, lacking the *fushi tarazu (ftz)* gene, which they argued may be the case for this cluster in all spiders. The second set of Hox genes was distributed across two scaffolds, which the authors attributed to incompleteness of the assembly due to patchy sequencing coverage [7]. For consistency, we will use the same nomenclature for Hox genes as used in [7] (*Abdominal-B: AbdB, Abdominal-A: AbdA, Ultrabithorax: Ubx, Antennapedia: Antp, fushi tarazu: ftz, sex combs reduced: scr, Deformed: Dfd, Hox3, proboscipedia: pb, labial: lab*). Corresponding with the results from *P. tepidariorum*, we found two clusters of Hox genes, with no evidence of tandem duplication. The two clusters occurred on two chromosomes (Chromosome 9 and Chromosome 6). In these locations, InterProScan generally annotated the genes as Hox genes but did not identify the specific type. On Chromosome 9, the Hox genes were in reverse collinear order, with no overlapping regions. Because it is complete, we will refer to this cluster as "Cluster A." On Chromosome 6, ("Cluster B") the genes were out of collinear order, with the position of *AbdA* and *Ubx* switched, and the coordinates for *Dfd, Hox3* and *pb* from the blast search overlapping (Figure 3A). The hits for *Antp* and *ftz* in Cluster B fell onto a single predicted gene in the annotation. Thus, it is unclear if *A. bruennichi* lacks one copy of *ftz*, as in *P. tepidariorum*, or if the annotation incorrectly fused the two genes in this cluster. In the study by Schwager *et al.,* 2017 [7], low sequencing coverage of Cluster B downstream of *Dfd* limited their inference. In our genome assembly, by mapping the PacBio reads against the final assembly, we calculated that we have an average of more than 12X coverage across the length of both clusters, suggesting that Cluster B is not out of order due to problems arising from low

coverage. It is possible that Hox Cluster B in spiders has changed or lost functionality following the proposed ancestral WGD event. The presence of two Hox clusters in our assembly is suggestive, but not evidence, of WGD in *A. bruennichi,* as it could have also arisen from duplication of only the Hox-containing chromosome; future studies can capitalize on the now-available chromosome-level assemblies for several groups (e.g. horseshoe crabs, ticks, and our spider) [72,88] to do more detailed analyses of duplication across chelicerates.

Spidroin genes

There are seven classes of silk produced by araneomorph spiders, each with one or more unique uses; it is important to note that the uses of these silk types are best understood for spiders in the family Araneidae, and the number and uses of silk types can vary widely between families [28,29,89,90]. The classes of silk are major ampullate (*MaSp*) minor ampullate (*MiSp*), piriform (*PiSp*), aggregate (*AgSp*), aciniform (*AcSp*) tubuliform (also referred to as cylindrical) (*TuSp*) and flagelliform (*Flag*). In *A. bruennichi*, spidroin genes occur on eight out of the thirteen chromosome scaffolds (Chromosomes 1, 3, 4, 6, 8, 11, 12 and 13) (Figure 3B). There were no hits on smaller scaffolds. We found four unique hits for *AcSp,* six hits for *AgSp,* one hit for *Flag,* eleven hits for *MaSp,* three hits for *MiSp,* one hit for *PiSp* and four hits for *TuSp.* In the majority of cases, all blast ~~matches~~ hits for a single spidroin type occurred on a single chromosome; the only exception was for *AgSp*, which had hits on four different chromosomes. However, these were not all annotated as spidroins; on Chromosome 6 there were two~~multiple~~ hits which were annotated as spidroins and one hit which was annotated as a chitin binding domain, while on Chromosome 4 the hit was annotated as tropoelastin, on Chromosome 3 the hit was

annotated as a chitin binding domain, and on Chromosome 8 the hit was annotated as a serine protease. All hits for *TuSp* occurred on Chromosome 1, but there were hits in two physically separated ~~clusters on~~areas of the chromosome; in one region there were hits on three annotated genes, and only one hit in the other region. There are more sequences available for *MaSp* than any of the other spidroin types in the genus *Argiope*, which allowed us to find matches for several unique *MaSp* genes in the *A. bruennichi* assembly. These occur in a small region of Chromosome 12, in close proximity to one another, suggesting that the spidroin genes in this group may have diversified via tandem duplication, as has been suggested in previous studies [91].

Venom genes

We found high identity matches for venom toxins on five of the chromosome scaffolds (Chromosomes 1, 2, 7, 10 and 11) (Figure 3B), but the majority of hits were on Chromosome 1. In most cases, each region containing venom gene matches contained only one gene, with the exception of a region on Chromosome 1, which contained five genes in very close proximity to one another, and two other regions (on Chromosome 1 and Chromosome 11), which contained matches to two genes. Babb *et al.* 2017 conducted a study on silk genes in *Trichonephila clavipes* (formerly *Nephila clavipes*), in which they found a novel flagelliform-type gene (FLAG-b) which was expressed most highly in the venom glands, not the flagelliform silk glands. This added to previous findings in the *Stegodyphus mimosarum* genome, where spidroin-like proteins are also found in the venom glands ~~are found~~ [27]. Interestingly, in the *A. bruennichi* genome assembly, there are several venom genes on Chromosome 11 in close proximity to the flagelliform spidroin gene~~s~~.

Conclusions

We have assembled and annotated the first chromosome-level genome for a~~n arachnid~~ spider. The assembly approach of combining long read, short read, and proximity ligation data overcame the challenges of assembling arachnid genomes, namely genome size, high repetitiveness, and low GC content. In our study, we made a preliminary analysis of the location of certain gene families of interest in the context of spider genomics, which hinted at several interesting directions for future studies on the evolution of silk and venom genes. Furthermore, because this species has undergone a recent and rapid range expansion, the well-resolved genome assembly will be useful for studies on the genomic underpinnings of range expansion and evolutionary adaptation to novel climates.

Availability of supporting data

The final genome assembly and raw data from the PacBio and Hi-C libraries, as well as the annotation, have been deposited at NCBI under BioProject PRJNA629526 and will be available upon publication. A publicly accessible genome browser hub with the annotation, ~~and~~ raw transcriptome, and PacBio read coverage can be found on the UCSC Genome Browser server (~~hub name "Wasp spider hub"~~under "My Data" > "Track Hubs" > "My Hubs" enter the URL http://bioinf.uni-greifswald.de/hubs/argiope/hub.txt).

Availability of source code and requirements

All data required to replicate this work are available on NCBI and in the supplementary files.

Declarations

List of abbreviations

Abd-A: Abdominal-A; Abd-B: Abdominal-B; AcSp: aciniform spidroin*; AgSp:* aggregate spidroin*; Antp: Antennapedia;* AT: adenine thymine*;* bp: basepairs*;* BUSCO: Benchmarking Universal Single Copy Orthologs*;* DDBJ: DNA Data Bank of Japan*; Dfd: Deformed;* DNA: deoxyribonucleic acid; *Flag:* flagelliform spidroin*; ftz: fushi tarazu;* Gb: gigabase pairs*;* GC: guanine cytosine*;* kb: kilobase pairs*; lab: labial;* LINE: long interspersed nuclear element*;* LTR: long terminal repeat*; MaSp:* major ampullate spidroin*;* Mb: megabase pairs*; MiSp:* minor ampullate spidroin*;* NCBI: National Center for Biotechnology Information*;* PacBio: Pacific Biosciences*; pb: proboscipedia;* PCR: polymerase chain reaction; *PiSp:* piriform spidroin*;* RNA: ribonucleic acid; *scr: sex combs reduced;* SINE: short interspersed nuclear element*; TuSp:* tubuliform spidroin*; Ubx: Ultrabithorax;* WGD: whole-genome duplication

Consent for publication

Not applicable.

Competing interests

The authors declare that they have no competing interests.

Funding

Funding for this study was provided by the Deutsche Forschungsgemeinschaft (DFG) as part of the Research Training Group 2010 RESPONSE (GRK 2010) to GU.

Authors' contributions

MMS, HK, GU, and SP conceived of the study; MMS, HK, and GU collected the spiders. HK extracted DNA for the PacBio sequencing; MMS prepared and submitted the DNA for

PacBio sequencing, with input and infrastructure provided by RGG. MMS and CJ constructed and sequenced the Hi-C library, with input and infrastructure provided by LJ and AK. MMS, AH and SP performed the genome assembly, and AH and KJH performed the genome annotation with input and infrastructure provided by MMS and SP. AH and KJH analyzed the repeat content of other ~~spider genomes~~arthropod species; MMS performed the analysis of ~~whole genome~~Hox cluster duplication, spidroin genes, and venom genes. MMS, AH, KJH and SP wrote the first draft of the manuscript. All authors read and approved the final manuscript.

Acknowledgements

We would like to thank the California Academy of Sciences for allowing us access to their computing resources for the genome assembly, and to Dovetail Genomics for their support in troubleshooting the Hi-C kit and running HiRise. MMS thanks José Cerca for helpful ideas and discussions about the silk and venom gene analysis.

References

1. Wise DH. Spiders in Ecological Webs. Cambridge: Cambridge University Press; 1993.

2. Spiller DA, Schoener TW. Effects of top and intermediate predators in a terrestrial food web. Ecology. Ecological Society of America; 1994;75:182–96.

3. Moulder BC, Reichle DE. Significance of spider predation in the energy dynamics of forest-floor arthropod communities. Ecol Monogr. Wiley; 1972;42:473–98.

4. Wirta HK, Weingartner E, Hambäck PA, Roslin T. Extensive niche overlap among the dominant arthropod predators of the High Arctic. Basic Appl Ecol. Elsevier GmbH;

2015;16:86–92.

5. Krehenwinkel H, Rödder D, Tautz D. Eco-genomic analysis of the poleward range expansion of the wasp spider *Argiope bruennichi* shows rapid adaptation and genomic admixture. Glob Chang Biol. 2015;21:4320–32.

6. Garb JE, González A, Gillespie RG. The black widow spider genus *Latrodectus* (Araneae: Theridiidae): Phylogeny, biogeography, and invasion history. Mol Phylogenet Evol. Academic Press Inc.; 2004;31:1127–42.

7. Schwager EE, Sharma PP, Clarke T, Leite DJ, Wierschin T, Pechmann M, et al. The house spider genome reveals an ancient whole-genome duplication during arachnid evolution. BMC Biol. BMC Biology; 2017;15:1–27.

8. Clarke TH, Garb JE, Hayashi CY, Arensburger P, Ayoub NA. Spider Transcriptomes Identify Ancient Large-Scale Gene Duplication Event Potentially Important in Silk Gland Evolution. Genome Biol Evol. 2015;7:1856–70.

9. Gendreau KL, Haney RA, Schwager EE, Wierschin T, Stanke M, Richards S, et al. House spider genome uncovers evolutionary shifts in the diversity and expression of black widow venom proteins associated with extreme toxicity. BMC Genomics. BMC Genomics; 2017;18:178.

10. Carson HL, Clayton FE, Stalker HD. Karyotypic stability and speciation in Hawaiian Drosophila. Proc Natl Acad Sci U S A. National Academy of Sciences; 1967;57:1280–5.

11. Řezáč M, Arnedo MA, Opatova V, Musilová J, Řezáčová V, Král J. Taxonomic revision and insights into the speciation mode of the spider *Dysdera erythrina* species-complex (Araneae: Dysderidae): Sibling species with sympatric distributions. Invertebr

Syst. CSIRO; 2018;32:10–54.

12. Mérot C, Oomen RA, Tigano A, Wellenreuther M. A roadmap for understanding the evolutionary significance of structural genomic variation. Trends Ecol Evol. Elsevier Ltd; 2020;35:561–72.

13. Shchur V, Svedberg J, Medina P, Corbett-Detig R, Nielsen R. On the distribution of tract lengths during adaptive introgression. G3 Genes, Genomes, Genet. 2020;10:3663–73.

14. Fuller ZL, Koury SA, Phadnis N, Schaeffer SW. How chromosomal rearrangements shape adaptation and speciation: Case studies in *Drosophila pseudoobscura* and its sibling species *Drosophila persimilis*. Mol Ecol. Blackwell Publishing Ltd; 2019;28:1283–301.

15. Faria R, Navarro A. Chromosomal speciation revisited: Rearranging theory with pieces of evidence. Trends Ecol Evol. Elsevier Current Trends; 2010;25:660–9.

16. White MJD. Chromosomal rearrangements and speciation in animals. Annu Rev Genet. 1969;3:75–98.

17. Rieseberg LH. Chromosomal rearrangements and speciation. Trends Ecol Evol. Elsevier Current Trends; 2001;16:351–8.

18. Noor MAF, Gratos KL, Bertucci LA, Reiland J. Chromosomal inversions and the reproductive isolation of species. Proc Natl Acad Sci U S A. National Academy of Sciences; 2001;98:12084–8.

19. Yannic G, Basset P, Hausser J. Chromosomal rearrangements and gene flow over time in an inter-specific hybrid zone of the *Sorex araneus* group. Heredity (Edinb).

Nature Publishing Group; 2009;102:616–25.

20. Feulner PGD, De-Kayne R. Genome evolution, structural rearrangements and speciation. Artic J Evol Biol. 2017;30:1488–90.

21. Castiglia R. Sympatric sister species in rodents are more chromosomally differentiated than allopatric ones: Implications for the role of chromosomal rearrangements in speciation. Mamm Rev. Blackwell Publishing Ltd; 2014;44:1–4.

22. Wellenreuther M, Mérot C, Berdan E, Bernatchez L. Going beyond SNPs: The role of structural genomic variants in adaptive evolution and species diversification. Mol Ecol. Blackwell Publishing Ltd; 2019;28:1203–9.

23. Vijay N, Bossu CM, Poelstra JW, Weissensteiner MH, Suh A, Kryukov AP, et al. Evolution of heterogeneous genome differentiation across multiple contact zones in a crow species complex. Nat Commun. Nature Publishing Group; 2016;7:1–10.

24. Turner TL, Hahn MW, Nuzhdin S V. Genomic islands of speciation in *Anopheles gambiae*. PLoS Biol. 2005;3:1572–8.

25. Hejase HA, Salman-Minkov A, Campagna L, Hubisz MJ, Lovette IJ, Gronau I, et al. Genomic islands of differentiation in a rapid avian radiation have been driven by recent selective sweeps. bioRxiv. Cold Spring Harbor Laboratory; 2020;2020.03.07.977694.

26. Duranton M, Allal F, Fraïsse C, Bierne N, Bonhomme F, Gagnaire PA. The origin and remolding of genomic islands of differentiation in the European sea bass. Nat Commun. Nature Publishing Group; 2018;9:1–11.

27. Sanggaard KW, Bechsgaard JS, Fang X, Duan J, Dyrlund TF, Gupta V, et al. Spider genomes provide insight into composition and evolution of venom and silk. Nat

Commun. Nature Publishing Group; 2014;5:3765.

28. Babb PL, Lahens NF, Correa-Garhwal SM, Nicholson DN, Kim EJ, Hogenesch JB, et al. The *Nephila clavipes* genome highlights the diversity of spider silk genes and their complex expression. Nat Genet. Nature Publishing Group; 2017;49:895–903.

29. Kono N, Nakamura H, Ohtoshi R, Moran DAP, Shinohara A, Yoshida Y, et al. Orb-weaving spider *Araneus ventricosus* genome elucidates the spidroin gene catalogue. Sci Rep. Nature Publishing Group; 2019;9:8380.

30. Yu N, Li J, Liu M, Huang L, Bao H, Yang Z, et al. Genome sequencing and neurotoxin diversity of a wandering spider *Pardosa pseudoannulata* (pond wolf spider). bioRxiv. 2019;747147.

31. Liu S, Aagaard A, Bechsgaard J, Bilde T. DNA methylation patterns in the social spider, *Stegodyphus dumicola*. Genes (Basel). 2019;10:137.

32. Sánchez-Herrero JF, Frías-López C, Escuer P, Hinojosa-Alvarez S, Arnedo MA, Sánchez-Gracia A, et al. The draft genome sequence of the spider *Dysdera silvatica* (Araneae, Dysderidae): A valuable resource for functional and evolutionary genomic studies in chelicerates. Gigascience. 2019;8:giz099.

33. Thomas GWC, Dohmen E, Hughes DST, Murali SC, Poelchau M, Glastad K, et al. Gene content evolution in the arthropods. Genome Biol. BioMed Central Ltd.; 2020;21:15.

34. Stellwagen SD, Renberg RL. Toward spider glue: Long read scaffolding for extreme length and repetitious silk family genes AgSp1 and AgSp2 with insights into functional adaptation. G3 Genes, Genomes, Genet. Genetics Society of America; 2019;9:1909–

19.

35. Ayoub NA, Garb JE, Kuelbs A, Hayashi CY. Ancient properties of spider silks revealed by the complete gene sequence of the prey-wrapping silk protein (AcSp1). Mol Biol Evol. 2013;30:589–601.

36. Krehenwinkel H, Tautz D. Northern range expansion of European populations of the wasp spider *Argiope bruennichi* is associated with global warming-correlated genetic admixture and population-specific temperature adaptations. Mol Ecol. 2013;22:2232–48.

37. Wawer W, Rutkowski R, Krehenwinkel H, Lutyk D, Pusz- K. Population structure of the expansive wasp spider (*Argiope bruennichi*) at the edge of its range. J Arachnol. 2017;45:361–9.

38. Krehenwinkel H, Graze M, Rödder D, Tanaka K, Baba YG, Muster C, et al. A phylogeographical survey of a highly dispersive spider reveals eastern Asia as a major glacial refugium for Palaearctic fauna. J Biogeogr. Wiley/Blackwell (10.1111); 2016;43:1583–94.

39. Wolz M, Klockmann M, Schmitz T, Pekár S, Bonte D, Uhl G. Dispersal and life-history traits in a spider with rapid range expansion. Mov Ecol 2019 81. BioMed Central; 2020;8:1–11.

40. Fromhage L, Uhl G, Schneider JM. Fitness consequences of sexual cannibalism in female *Argiope bruennichi*. Behav Ecol Sociobiol. Springer-Verlag; 2003;55:60–4.

41. Schneider JM, Fromhage L, Uhl G. Extremely short copulations do not affect hatching success in *Argiope bruennichi* (Araneae, Araneidae). J Arachnol. American

Arachnological Society; 2005;33:663–9.

42. Schneider J, Uhl G, Herberstein ME. Cryptic female choice within the genus Argiope: A comparative approach. In: Peretti A, Aisenberg A, editors. Cryptic Female Choice Arthropods Patterns, Mech Prospect. Cham: Springer International Publishing; 2015. p. 55–77.

43. Chinta SP, Goller S, Lux J, Funke S, Uhl G, Schulz S. The sex pheromone of the wasp spider *Argiope bruennichi*. Angew Chemie - Int Ed. Wiley-Blackwell; 2010;49:2033–6.

44. Uhl G, Zimmer SM, Renner D, Schneider JM. Exploiting a moment of weakness: male spiders escape sexual cannibalism by copulating with moulting females. Sci Rep. Nature Publishing Group; 2015;5:16928.

45. Gregory TR, Shorthouse DP. Genome sizes of spiders. J Hered. Oxford Academic; 2003;94:285–90.

46. Rasch EM, Connelly BA. Genome size and endonuclear DNA replication in spiders. J Morphol. John Wiley & Sons, Ltd; 2005;265:209–14.

47. Gregory TR. Animal Genome Size Database [Internet]. 2020. Available from: http://www.genomesize.com/index.php

48. Schell T, Feldmeyer B, Schmidt H, Greshake B, Tills O, Truebano M, et al. An Annotated Draft Genome for *Radix auricularia* (Gastropoda, Mollusca). Genome Biol Evol. Oxford University Press (OUP); 2017;9:585–92.

49. Li H, Handsaker B, Wysoker A, Fennell T, Ruan J, Homer N, et al. The Sequence Alignment/Map format and SAMtools. Bioinformatics. 2009;25:2078–9.

50. Li H. Aligning sequence reads, clone sequences and assembly contigs with BWA-MEM. 2013;

51. Okonechnikov K, Conesa A, García-Alcalde F. Qualimap 2: Advanced multi-sample quality control for high-throughput sequencing data. Bioinformatics. Oxford University Press; 2016;32:292–4.

52. R Core Team. R: A language and environment for statistical computing. Vienna, Austria: R Foundation for Statistical Computing; 2017.

53. Ewels P, Magnusson M, Lundin S, Käller M. MultiQC: Summarize analysis results for multiple tools and samples in a single report. Bioinformatics. Oxford University Press; 2016;32:3047–8.

54. Quinlan AR, Hall IM. BEDTools: A flexible suite of utilities for comparing genomic features. Bioinformatics. Oxford Academic; 2010;26:841–2.

55. Li H. Minimap2: Pairwise alignment for nucleotide sequences. Bioinformatics. Oxford University Press; 2018;34:3094–100.

56. Ruan J, Li H. Fast and accurate long-read assembly with wtdbg2. Nat Methods. Nature Research; 2020;17:155–8.

57. Walker BJ, Abeel T, Shea T, Priest M, Abouelliel A, Sakthikumar S, et al. Pilon: An integrated tool for comprehensive microbial variant detection and genome assembly improvement. Wang J, editor. PLoS One. 2014;9:e112963.

58. Simão FA, Waterhouse RM, Ioannidis P, Kriventseva E V., Zdobnov EM. BUSCO: assessing genome assembly and annotation completeness with single-copy orthologs. Bioinformatics. 2015;31:3210–2.

59. Putnam NH, O'Connell BL, Stites JC, Rice BJ, Blanchette M, Calef R, et al. Chromosome-scale shotgun assembly using an in vitro method for long-range linkage. Genome Res. Cold Spring Harbor Laboratory Press; 2016;26:342–50.

60. Gurevich A, Saveliev V, Vyahhi N, Tesler G. QUAST: Quality assessment tool for genome assemblies. Bioinformatics. 2013;29:1072–5.

61. Zhang YJ, Tong SJ. The routine method for preparing the chromosomes in spiders. Chinese J Zool. 1990;25:30–1.

62. Araujo D, Mattos VF, Giroti AM, Kraeski MG, Carvalho LS, Brescovit AD. Cytogenetical characterization of six orb-weaver species and review of cytogenetical data for Araneidae. J Arachnol. 2011;39:337–44.

63. Mapleson D, Accinelli GG, Kettleborough G, Wright J, Clavijo BJ. KAT: A K-mer analysis toolkit to quality control NGS datasets and genome assemblies. Bioinformatics. Oxford University Press; 2017;33:574–6.

64. Smit AFA, Hubley R. RepeatModeler-1.0. 2008.

65. Smit AFA, Hubley R. RepeatMasker-4.0. 2013.

66. Laetsch DR, Blaxter ML. BlobTools: Interrogation of genome assemblies. F1000Research. F1000 Research, Ltd.; 2017;6:1287.

67. Kim D, Paggi JM, Park C, Bennett C, Salzberg SL. Graph-based genome alignment and genotyping with HISAT2 and HISAT-genotype. Nat Biotechnol. Nature Publishing Group; 2019;37:907–15.

68. Hoff KJ, Stanke M. Predicting Genes in Single Genomes with AUGUSTUS. Curr Protoc Bioinforma. 2018;e57.

69. Jones P, Binns D, Chang HY, Fraser M, Li W, McAnulla C, et al. InterProScan 5: Genome-scale protein function classification. Bioinformatics. 2014;30:1236–40.

70. Quevillon E, Silventoinen V, Pillai S, Harte N, Mulder N, Apweiler R, et al. InterProScan: Protein domains identifier. Nucleic Acids Res. 2005;33:W116–20.

71. Brůna T, Hoff KJ, Lomsadze A, Stanke M, Borodovsky M. BRAKER2: Automatic Eukaryotic Genome Annotation with GeneMark-EP+ and AUGUSTUS Supported by a Protein Database. bioRxiv. 2020;

72. Jia N, Wang J, Shi W, Du L, Sun Y, Zhan W, et al. Large-Scale Comparative Analyses of Tick Genomes Elucidate Their Genetic Diversity and Vector Capacities. Cell. 2020;1–13.

73. Dasmahapatra KK, Walters JR, Briscoe AD, Davey JW, Whibley A, Nadeau NJ, et al. Butterfly genome reveals promiscuous exchange of mimicry adaptations among species. Nature. Nature Publishing Group; 2012;487:94–8.

74. Kim HS, Murphy T, Xia J, Caragea D, Park Y, Beeman RW, et al. BeetleBase in 2010: Revisions to provide comprehensive genomic information for *Tribolium castaneum*. Nucleic Acids Res. Oxford University Press; 2009;38:D437.

75. Qu Z, Nong W, So WL, Barton-Owen T, Li Y, Li C, et al. Millipede genomes reveal unique adaptation of genes and microRNAs during myriapod evolution. bioRxiv. Cold Spring Harbor Laboratory; 2020;2020.01.09.900019.

76. Miller JR, Koren S, Dilley KA, Harkins DA, Stockwell TB, Shabman RS, et al. A draft genome sequence for the *Ixodes scapularis* cell line, ISE6. F1000Research. F1000 Research Ltd; 2018;7:297.

77. Pearson JC, Lemons D, McGinnis W. Modulating Hox gene functions during animal body patterning. Nat Rev Genet. Nature Publishing Group; 2005;6:893–904.

78. Gatesy J, Hayashi C, Motriuk D, Woods J, Lewis R. Extreme diversity, conservation, and convergence of spider silk fibroin sequences. Science (80-). American Association for the Advancement of Science; 2001;291:2603–5.

79. Hayashi CY, Shipley NH, Lewis R V. Hypotheses that correlate the sequence, structure, and mechanical properties of spider silk proteins. Int J Biol Macromol. Elsevier; 1999;24:271–5.

80. Casewell NR, Wü Ster W, Vonk FJ, Harrison RA, Fry BG. Complex cocktails: the evolutionary novelty of venoms. Trends Ecol Evol. 2013;28:219–29.

81. Fry BG, Roelants K, Champagne DE, Scheib H, Tyndall JDA, King GF, et al. The toxicogenomic multiverse: Convergent recruitment of proteins into animal venoms. Annu Rev Genomics Hum Genet. 2009;10:483–511.

82. Grishin E. Polypeptide neurotoxins from spider venoms. Eur J Biochem. John Wiley & Sons, Ltd; 1999;264:276–80.

83. Escoubas P. Molecular diversification in spider venoms: A web of combinatorial peptide libraries. Mol Divers. 2006;10:545–54.

84. Escoubas P, Sollod B, King GF. Venom landscapes: Mining the complexity of spider venoms via a combined cDNA and mass spectrometric approach. Toxicon. Pergamon; 2006;47:650–63.

85. Diniz CR, do Nascimento Cordeiro M, Junor LR, Kelly P, Fischer S, Reimann F, et al. The purification and amino acid sequence of the lethal neurotoxin Tx1 from the

venom of the Brazilian ‘armed’ spider *Phoneutria nigriventer*. FEBS Lett. 1990;263:251–3.

86. Gerts EM, Yu YK, Agarwala R, Schäffer AA, Altschul SF. Composition-based statistics and translated nucleotide searches: Improving the TBLASTN module of BLAST. BMC Biol. BioMed Central; 2006;4:41.

87. Altschul SF, Gish W, Miller W, Myers EW, Lipman DJ. Basic local alignment search tool. J Mol Biol. Academic Press; 1990;215:403–10.

88. Shingate P, Ravi V, Prasad A, Tay BH, Garg KM, Chattopadhyay B, et al. Chromosome-level assembly of the horseshoe crab genome provides insights into its genome evolution. Nat Commun. Springer US; 2020;11:2322.

89. Vollrath F. Biology of spider silk. Int J Biol Macromol. 1999;24:81–8.

90. Blackledge TA, Hayashi CY. Silken toolkits: Biomechanics of silk fibers spun by the orb web spider *Argiope argentata* (Fabricius 1775). J Exp Biol. The Company of Biologists Ltd; 2006;209:2452–61.

91. Zhao Y, Ayoub NA, Hayashi CY. Chromosome mapping of dragline silk genes in the genomes of widow spiders (Araneae, Theridiidae). PLoS One. 2010;5:e12804.

92. Durand NC, Robinson JT, Shamim MS, Machol I, Mesirov JP, Lander ES, et al. Juicebox Provides a Visualization System for Hi-C Contact Maps with Unlimited Zoom. Cell Syst. Cell Press; 2016;3:99–101.

Figure Legends

Figure 1: Female *Argiope bruennichi* spider in orb web from Loulé (Faro, Portugal). Photo credit: Monica M. Sheffer

Figure 2: Genome assembly completeness. (A) Contact heatmap of Hi-C scaffolding shows long-range contacts of paired-end Hi-C reads. Gray lines denote scaffold (chromosome) boundaries. Visualized with Juicebox (v. 1.11.08) [92]. (B) The length of the 20 longest scaffolds in the assembly shows that the 13 putative chromosome scaffolds are dramatically larger than the next largest. Red points represent individual scaffolds, ordered from largest to smallest. (~~B~~C) Cumulative length of assembly contained within ~~contigs~~scaffolds. Note that the vast majority (98.4%) of the genome is contained within very few ~~(13) contigs~~scaffolds. Visualized with QUAST v. 5.0.2 [60] using default parameters, except --min-contig 0. ~~(C) The length of the 20 longest scaffolds in the assembly shows that the 13 putative chromosome scaffolds are dramatically larger than the next largest. Red points represent individual scaffolds, ordered from largest to smallest.~~

Figure 3: Schematic representation of location of gene families on the 13 chromosomes. (A) Hox gene clusters. Genes connected by a black line occur on the same scaffold. Cluster A occurs on Chromosome 9, and Cluster B occurs on Chromosome 6. The presence of two Hox gene clusters on two chromosomes ~~validates the previous finding~~ is suggestive of whole~~-~~genome duplication in *Argiope bruennichi*, as was found previously for *Parasteatoda tepidariorum* [7]. (B) Position of Hox, spidroin and venom genes on chromosome scaffolds. The light grey bars represent chromosomes, the colored rectangles represent the seven different spidroin gene

families, the black rectangles represent venom genes, and the white rectangles represent Hox gene clusters. Numbers inside of the rectangles represent the number of genes found within that cluster.

Figure 1

Click here to access/download;Figure;Figure1_Argiope_bruennichi_MertolaPo

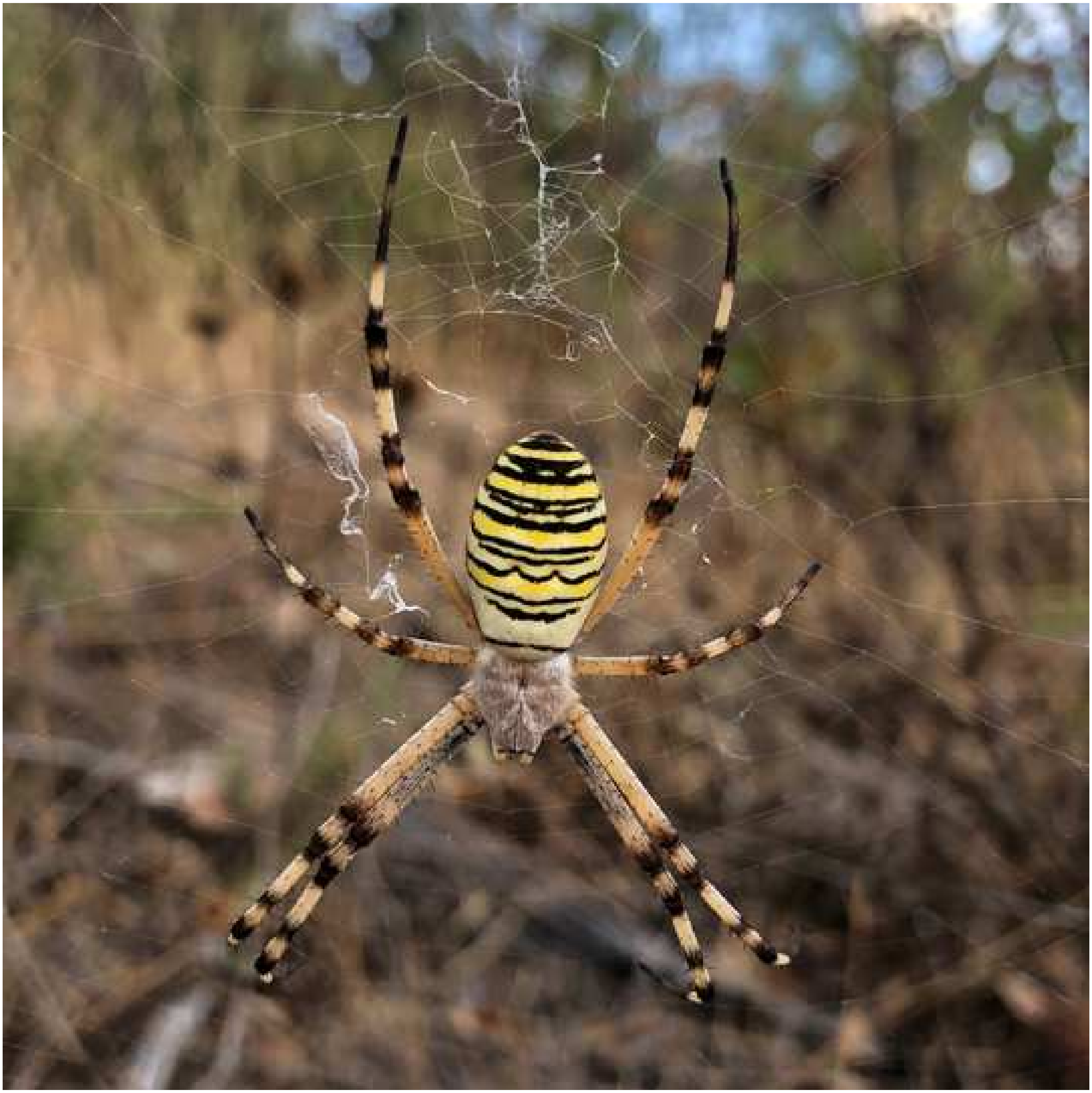

Figure 2

Click here to access/download;Figure;Figure2_assembly_figure

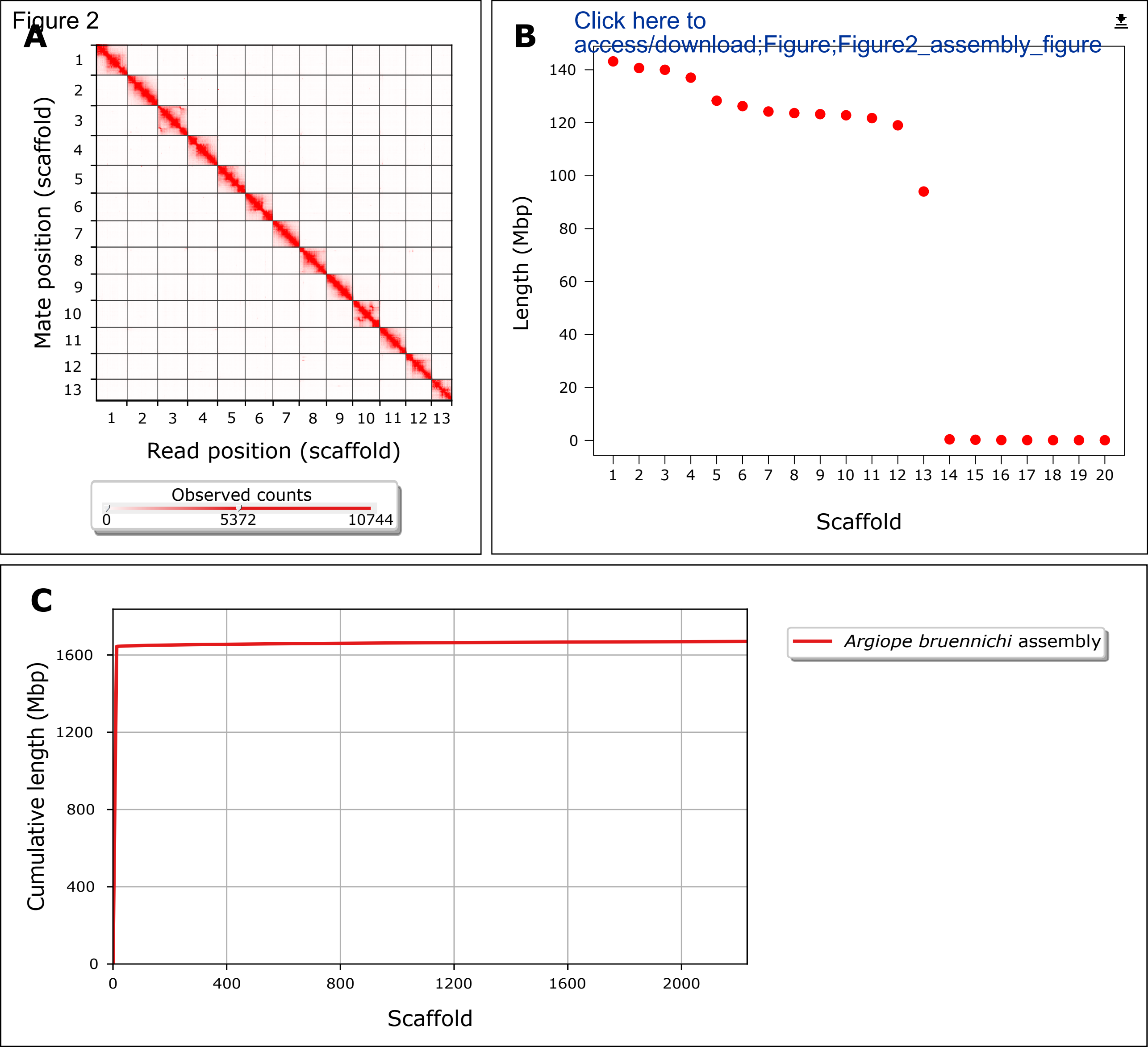

Figure 3

Click here to access/download;Figure;Figure3_

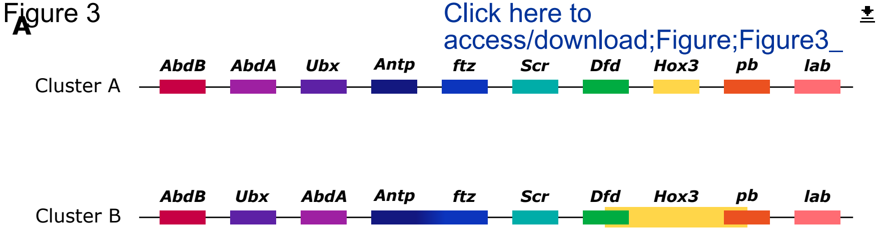

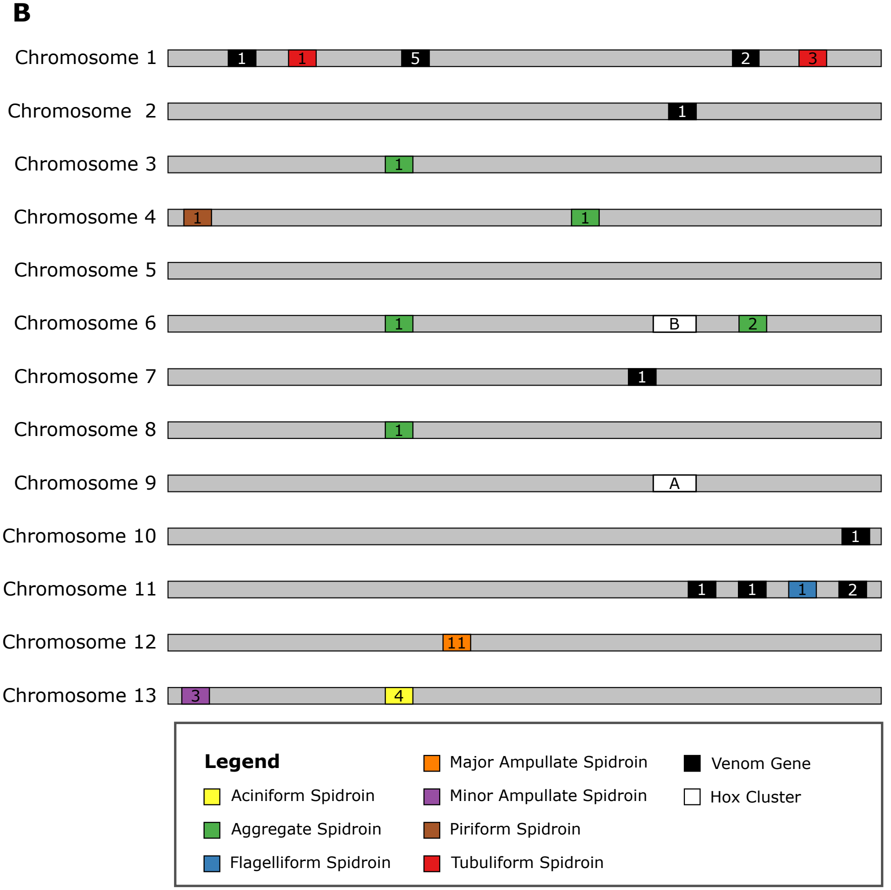

Supplementary Figure 1

Click here to access/download
Supplementary Material
SupplementaryFigure1_minorScaffoldLengths.png

Supplementary Figure 2

Click here to access/download
Supplementary Material
SupplementaryFigure2_RepeatContent.png

Supplementary File 1

Click here to access/download
Supplementary Material
SupplementaryFile1_hox_blastQuery.fasta

Supplementary File 2

Click here to access/download
Supplementary Material
SupplementaryFile2_spidroin_blastQuery.fasta

Supplementary File 3

Click here to access/download
Supplementary Material
SupplementaryFile3_venom_blastQuery.fasta

Supplementary Table 1

Click here to access/download
Supplementary Material
SupplementaryTable1_SpiderGenomeAssemblies.xlsx

Supplementary Table 2

Click here to access/download
Supplementary Material
SupplementaryTable2_RepetitiveContentSpiders.xlsx

Supplementary Table 3

Click here to access/download
Supplementary Material
SupplementaryTable3_hox_blastResults.xlsx

Supplementary Table 4

Click here to access/download
Supplementary Material
SupplementaryTable4_spidroin_blastResults_revised.xls
x

Supplementary Table 5

Click here to access/download
Supplementary Material
SupplementaryTable5_venom_blastResults.xlsx
