# Supplementary material for: Chromosome-level reference genome of the European wasp spider Argiope bruennichi: a resource for studies on range expansion and evolutionary adaptation
Source: Gigascience. 2021 Jan 7;10(1):giaa148. doi: 10.1093/gigascience/giaa148 (PMC7788392; doi:10.1093/gigascience/giaa148)
Supplement: giaa148_GIGA-D-20-00146_Revision_2 [file giaa148_giga-d-20-00146_revision_2.pdf]

GigaScience

Chromosome-level reference genome of the European wasp spider Argiope bruennichi: a resource for studies on range expansion and evolutionary adaptation

--Manuscript Draft--

Manuscript Number:	GIGA-D-20-00146R2
Full Title:	Chromosome-level reference genome of the European wasp spider Argiope bruennichi: a resource for studies on range expansion and evolutionary adaptation
Article Type:	Data Note
Funding Information:	Deutsche Forschungsgemeinschaft (GRK 2010) — Dr. Gabriele Uhl
Abstract:	Background<br><br>Argiope bruennichi, the European wasp spider, has been investigated intensively as a focal species for studies on sexual selection, chemical communication, and the dynamics of rapid range expansion at a behavioral and genetic level. However, the lack of a reference genome has limited insights into the genetic basis for these phenomena. Therefore, we assembled a high-quality chromosome-level reference genome of the European wasp spider as a tool for more in-depth future studies.<br><br>Findings<br><br>We generated, de novo, a 1.67Gb genome assembly of A. bruennichi using 21.8X PacBio sequencing, polished with 19.8X Illumina paired-end sequencing data, and proximity ligation (Hi-C) based scaffolding. This resulted in an N50 scaffold size of 124Mb and an N50 contig size of 288kb. We found 98.4% of the genome to be contained in 13 scaffolds, fitting the expected number of chromosomes (n = 13). Analyses showed the presence of 91.1% of complete arthropod BUSCOs, indicating a high quality assembly.<br><br>Conclusions<br><br>We present the first chromosome-level genome assembly in the order Araneae. With this genomic resource, we open the door for more precise and informative studies on evolution and adaptation not only in A. bruennichi, but also in arachnids overall, shedding light on questions such as the genomic architecture of traits, whole-genome duplication and the genomic mechanisms behind silk and venom evolution.
Corresponding Author:	Monica M Sheffer<br>Zoological Institute and Museum, University of Greifswald, Germany<br>Greifswald, GERMANY
Corresponding Author Secondary Information:	
Corresponding Author's Institution:	Zoological Institute and Museum, University of Greifswald, Germany
Corresponding Author's Secondary Institution:	
First Author:	Monica M. Sheffer
First Author Secondary Information:	
Order of Authors:	Monica M. Sheffer
	Anica Hoppe
	Henrik Krehenwinkel
	Gabriele Uhl
	Andreas W. Kuss
	Lars Jensen
	Corinna Jensen

Powered by Editorial Manager® and ProduXion Manager® from Aries Systems Corporation

	Rosemary G. Gillespie
	Katharina J. Hoff
	Stefan Prost
**Order of Authors Secondary Information:**	
**Response to Reviewers:**	Dear Editor:<br><br>I am submitting the revised version our data note manuscript entitled, “Chromosome-level reference genome of the European wasp spider Argiope bruennichi: a resource for studies on range expansion and evolutionary adaptation” by Monica M. Sheffer, Anica Hoppe, Henrik Krehenwinkel, Gabriele Uhl, Andreas W. Kuss, Lars Jensen, Corinna Jensen, Rosemary G. Gillespie, Katharina J. Hoff and Stefan Prost (shared last authorship), with minor changes.<br><br>We have addressed the remaining comments from the second reviewer, following the latest round of review:<br>- We have included the KAT plots as a supplementary figure (Supplementary Figure S1, line 188), and removed our sentence suggesting that the use of different individuals may have been the reason for the missing kmer content (lines 188-192), as the reviewer found this unlikely.<br>- We have changed Figure 3 to focus solely on Hox duplication: Figure 3A, colinearity of the Hox genes, remains the same; Figure 3B now contains the circular chromosome viewer depicting conserved syntenic blocks between the Hox-containing chromosomes. What was formerly Figure 3B (displaying the location of gene families within the genome) now stands alone as Figure 4. We have updated the figure legends (lines 727-743) to correspond to these changes, and added a few sentences about the synteny analysis in the main text (lines 345-356).<br>- We have addressed all of the changes to word choice and sentence structure that the reviewer requested, and fixed the references as he indicated.<br><br>In addition to those reviewer-requested changes above, we have made a few in addition:<br>- We have included the citation to our GigaDB dataset, as provided by the data curators, and included the information on available file types in our “Availability of supporting data” section.<br>- We have corrected the estimation of ~30X coverage of Illumina reads (we based this on the coverage provided in the publication of those reads, but have now calculated the coverage ourselves) in the abstract and throughout the text (lines 40, 159-160, 165, 190). The coverage is in fact 19.8X.<br>- We have numbered the supplementary files with an “S” in front of the number, as is the norm in the journal, i.e. Supplementary Figure S1 instead of Supplementary Figure 1.<br>- In some references, the publisher was missing. We have added this throughout the references.<br>- We have removed the legend of Figure 2C, as it was unnecessary, and removed the boxes around the figures within Figure 2, as they did not fit the aesthetic of the other figures.<br>- Slight changes to punctuation and sentence structure were also made.<br><br>Per our previous communication, we would like to expedite the process of publication of our manuscript as much as possible, so that we can pay the Article Processing Charges with the remainder of our 2020 budget.<br><br>We would like to, once again, express our gratitude for the thorough work of both reviewers, and the efficient and helpful work of the handling editor, which have made this process very positive and constructive. We look forward to the publication of our article.<br><br>Sincerely,<br>Monica M. Sheffer, on behalf of all co-authors
**Additional Information:**	
**Question**	**Response**

Powered by Editorial Manager® and ProduXion Manager® from Aries Systems Corporation

Are you submitting this manuscript to a special series or article collection?	No
**Experimental design and statistics**<br><br>Full details of the experimental design and statistical methods used should be given in the Methods section, as detailed in our Minimum Standards Reporting Checklist. Information essential to interpreting the data presented should be made available in the figure legends.<br><br>Have you included all the information requested in your manuscript?	Yes
**Resources**<br><br>A description of all resources used, including antibodies, cell lines, animals and software tools, with enough information to allow them to be uniquely identified, should be included in the Methods section. Authors are strongly encouraged to cite Research Resource Identifiers (RRIDs) for antibodies, model organisms and tools, where possible.<br><br>Have you included the information requested as detailed in our Minimum Standards Reporting Checklist?	Yes
**Availability of data and materials**<br><br>All datasets and code on which the conclusions of the paper rely must be either included in your submission or deposited in publicly available repositories (where available and ethically appropriate), referencing such data using a unique identifier in the references and in the "Availability of Data and Materials" section of your manuscript.<br><br>Have you have met the above requirement as detailed in our Minimum	Yes

Standards Reporting Checklist?	

Powered by Editorial Manager® and ProduXion Manager® from Aries Systems Corporation

Manuscript
Click here to access/download;Manuscript;ArgiopeGenome-ForProduction.docx

Title

Chromosome-level reference genome of the European wasp spider *Argiope bruennichi*: a resource for studies on range expansion and evolutionary adaptation

Authors

Monica M. Sheffer[1#], Anica Hoppe[2,3], Henrik Krehenwinkel[4], Gabriele Uhl[1], Andreas W. Kuss[5], Lars Jensen[5], Corinna Jensen[5], Rosemary G. Gillespie[6], Katharina J. Hoff[2,3]* & Stefan Prost[7,8]*

[#]indicates corresponding author
*indicates equal contribution

Affiliations

[1] Zoological Institute and Museum, University of Greifswald, Greifswald, Germany

[2] Institute of Mathematics and Computer Science, University of Greifswald, Greifswald, Germany

[3] Center for Functional Genomics of Microbes, University of Greifswald, Greifswald, Germany

[4] Department of Biogeography, University of Trier, Trier, Germany

[5] Interfaculty Institute for Genetics and Functional Genomics, University of Greifswald, Greifswald, Germany

[6] Department of Environmental Science Policy and Management, University of California Berkeley, Berkeley, USA

[7] LOEWE-Centre for Translational Biodiversity Genomics, Senckenberg, Frankfurt Germany

[8] South African National Biodiversity Institute, National Zoological Gardens of South Africa, Pretoria, South Africa

E-Mails and ORCID

Name	E-Mail Address	ORCID
Monica M. Sheffer	monica.sheffer@uni-greifswald.de	0000-0002-6527-4198
Anica Hoppe	anica.hoppe@stud.uni-greifswald.de	0000-0002-2586-1654
Henrik Krehenwinkel	krehenwinkel@uni-trier.de	0000-0001-5069-8601
Gabriele Uhl	gabriele.uhl@uni-greifswald.de	0000-0001-8758-7913
Andreas W. Kuss	andreas.kuss@uni-greifswald.de	0000-0002-9401-4627
Lars Jensen	larsriff.jensen@uni-greifswald.de	
Corinna Jensen	corinna.jensen@uni-greifswald.de	
Rosemary G. Gillespie	gillespie@berkeley.edu	0000-0003-0086-7424
Katharina J. Hoff	katharina.hoff@uni-greifswald.de	0000-0002-7333-8390
Stefan Prost	stefanprost.research@protonmail.com	0000-0002-6229-3596

Abstract

Background: *Argiope bruennichi*, the European wasp spider, has been investigated intensively as a focal species for studies on sexual selection, chemical communication, and the dynamics of rapid range expansion at a behavioral and genetic level. However, the lack of a reference genome has limited insights into the genetic basis for these phenomena. Therefore, we assembled a high-quality chromosome-level reference genome of the European wasp spider as a tool for more in-depth future studies.

Findings: We generated, *de novo*, a 1.67Gb genome assembly of *A. bruennichi* using 21.8X PacBio sequencing, polished with 19.8X Illumina paired-end sequencing data, and proximity ligation (Hi-C) based scaffolding. This resulted in an N50 scaffold size of 124Mb and an N50 contig size of 288kb. We found 98.4% of the genome to be contained in 13 scaffolds, fitting the expected number of chromosomes (n = 13). Analyses showed the presence of 91.1% of complete arthropod BUSCOs, indicating a high quality assembly.

Conclusions: We present the first chromosome-level genome assembly in the order Araneae. With this genomic resource, we open the door for more precise and informative studies on evolution and adaptation not only in *A. bruennichi*, but also in arachnids overall, shedding light on questions such as the genomic architecture of traits, whole-genome duplication and the genomic mechanisms behind silk and venom evolution.

Keywords

Argiope bruennichi, genome assembly, Araneae, spider, PacBio, Hi-C, chromosome-level, Hox duplication, silk, venom

Data description

Context

Spider genomes are of great interest, for instance in the context of silk and venom evolution and biomedical and technical applications. Additionally, spiders are fascinating from ecological and evolutionary perspectives. As the most important predators of terrestrial arthropods, they play a key role in terrestrial food webs [1–4]. Spiders are distributed on every continent except Antarctica, and diverse habitats can be occupied by single species or multiple close relatives [5,6], making them ideal for studies on environmental plasticity, adaptation and speciation. With regards to adaptation, work on cobweb spiders (Theridiidae) has revealed a whole-genome duplication that may facilitate diversification [7], with other studies highlighting a key role of tandem duplication and neofunctionalization of genes in the diversification and specialization of spider silks [8] and venoms [9]. A key aspect that has been missing from studies to date is the role of genome organization in facilitating or impeding adaptation, as there have been no studies to date on spiders that have provided a chromosomal framework for the genome.

Understanding the chromosomal organization of a genome is critical for identification of processes underlying divergence between populations, adaptation, and speciation. Indeed, the potential role of chromosomal reorganization in species formation has long been the subject of debate, in particular in *Drosophila* species where polytene chromosomes allowed early visualization of chromosomal rearrangements [10]. Among spiders, karyotype data are still used to identify changes in chromosomes associated with speciation [11]. With the advent of detailed genomic data, there has been renewed focus on the role that structural variants in the genome can play as drivers of adaptation and speciation, associated with translocations, fusions, and inversions [12], as well as with

admixture and associated demographic changes [13]. Recent data from sister species of the genus *Drosophila* suggest that the establishment of inversion polymorphisms within isolated and/or heterogeneous environments may well set the stage for species formation [14]. In order to develop a broader understanding of the role of structural variation in adaptation and speciation [15–22], we need chromosome-level genomes that provide the ability to map the order of genes, define chromosomal gene neighborhoods, and identify potential genomic islands of differentiation [23–26].

To the best of our knowledge, ten draft spider genomes have been published to date [7,27–33], most of which focus on silk and venom genes, while one discusses whole-genome duplication [7] and the publication of the most recent two focuses on gene content evolution across arthropods [33]. There is one additional, as yet unpublished, spider genome assembly available on NCBI (National Center for Biotechnology Information) (*Anelosimus studiosus,* accession number: GCA_008297655.1). Spider genomes are considered notoriously difficult to sequence, assemble, and annotate for a number of factors, including their relatively high repeat content, low guanine cytosine (GC) content, high levels of heterozygosity in the wild [27] and due to the fact that they possess some extremely long coding genes in the spidroin gene families [28,29,34,35]. Due to these challenges, the completeness of the available spider genomes varies greatly between assemblies (Supplementary Table S1). All of them are incomplete and there is no chromosome-level assembly published for any spider to date. While this does not lessen the conclusions of the above-mentioned studies, a chromosome-level assembly would open doors for more detailed studies on the genomic architecture of gene families, such as silk and venom genes, providing greater understanding of the evolutionary

mechanisms driving the diversification of these gene families and genome evolution, in addition to the aforementioned applications in understanding adaptation and speciation.

The European wasp spider, *Argiope bruennichi* (Scopoli, 1772), is an orb-weaving spider in the family Araneidae (Figure 1). Despite the lack of a reference genome, *A. bruennichi* has been the focal species for studies on local adaptation, range expansion, admixture, and biogeography [5,36–38]. These studies have suggested that the range expansion and subsequent local adaptation of *A. bruennichi* from southern to northern Europe was caused by genetic admixture. However, it is not yet known which regions of the genome are admixed, and if these regions are truly responsible for adaptation to colder climates. *A. bruennichi* has also been well studied in the context of dispersal and life history traits [39], as well as sexual selection and chemical communication (e.g. [40–44]). A high-quality reference genome would allow altogether new insights into our understanding of the genetic basis of these phenomena. Considering this background, a chromosome-level reference genome would be highly desirable for the species.

Sampling, DNA extraction and sequencing

Adult female *Argiope bruennichi* individuals (NCBI:txid94029) were collected in the south of Portugal in 2013 and 2019 (Latitude: 37.739 N, Longitude: -7.853 E). As inbred lines of the species do not exist, we selected a population which was previously found to have low heterozygosity in the wild, likely due to naturally high levels of inbreeding [5].

For the baseline assembly, deoxyribonucleic acid (DNA) was extracted from a female collected in 2013 using the ArchivePure blood and tissue kit (5 PRIME, Hamburg, Germany), according to the manufacturer's protocol. A ribonucleic acid (RNA) digestion

step was included using RNAse A solution (7000 U mL^{-1}; 5 PRIME). The DNA was stored at -80°C until library preparation in 2017. The DNA extract was cleaned using a salt:PCI cleaning step, and had a fragment size distribution from 1,300-165,500bp (peak at 14,002bp) before size selection. The library was size selected to 15 kilobasepairs (kb) using Pippin prep and subsequently sequenced in 2018 at the QB3 Genomics facility at the University of California Berkeley on a Pacific Biosciences Sequel I platform (PacBio, Menlo Park, CA, USA) on 10 cells.

The specimen collected in 2019 was used to build a proximity-ligation based short-read library ("Hi-C"). Four Hi-C libraries were prepared from a single individual using a Dovetail™ Hi-C library preparation kit according to the manufacturer's protocol (Dovetail Genomics, Santa Cruz, CA). The specimen was anesthetized with CO_2 before preparation. In brief, the legs were removed from the body and stored in liquid nitrogen, and the leg tissue was disrupted in liquid nitrogen using a mortar and pestle. Chromatin was fixed with formaldehyde, then extracted. Fixed chromatin was digested with DpnII, the 5' overhangs filled in with biotinylated nucleotides, and the free blunt ends were ligated. After ligation, crosslinks were reversed and the DNA purified to remove proteins. Purified DNA was treated to remove biotin that was not internal to ligated fragments. The DNA was then sheared to ~350bp mean fragment size using a Covaris S2 Focused-ultrasonicator. A typical Illumina library preparation protocol followed, with end repair and Illumina adapter ligation. Biotinylated fragments were captured with streptavidin beads before PCR (polymerase chain reaction) amplification (12 cycles), and size selection was performed using SPRI-select beads (Beckman Coulter GmbH, Germany) for a final library size distribution centered around 450bp. The library was sequenced to approximately 440

million paired end reads on one Flowcell of an Illumina NextSeq 550 with a High Output v2 kit (150 cycles).

Genome size estimation and coverage

We estimated the genome size of *Argiope bruennichi* based on data for closely related species, and bioinformatically based on previously published Illumina paired-end data derived from a single female individual from a population in Madeira (SRA accession number: ERX533198) [5], which we later used for polishing the assembly.

The closely related species *A. aurantia* and *A. trifasciata* have genome size estimates based on Feulgen densitometry data of 1.620 gigabasepairs (Gb) [45] or 1.650Gb [46] for *A. aurantia* and 1.690Gb for *A. trifasciata* [45,47]. Using the backmap.pl (v. 0.3) pipeline [48–55] on the Illumina data from *A. bruennichi* [5], we generated a genome size estimate of 1.740Gb. Averaging these four genome size measurements yields an estimate of 1.675Gb.

Given this estimate, the PacBio sequencing yielded 21.8X coverage (approximately 36.65Gb sequenced, with an estimated genome size of 1.675Gb). The previously published Illumina data [5] has a coverage of 19.8X (33.05Gb sequenced).

De novo genome assembly

First, we generated a baseline assembly using 21.8X long-read Pacific Biosciences (PacBio) Sequel I sequencing data and the wtdbg2 assembler (v. 2.3) (WTDBG, RRID:SCR_017225) [56]. Next, we polished the assembly by applying three rounds of Pilon (v. 1.23) (Pilon, RRID:SCR_014731) [57] using the 19.8X of previously published Illumina paired-end data [5]. Mapping for the three rounds of polishing resulted in a

mapping rate ranging from 92.55 to 93.69%. The polishing resulted in 13,843 contigs with an N50 of 288.4 kilobase pairs (kb), and an overall assembly size of 1.67Gb. Analysis of Benchmarking Universal Single Copy Orthologs (BUSCO) (v. 3.1.0) scores, using the arthropod data set (BUSCO, RRID:SCR_015008) [58], showed the presence of 90.2% of complete BUSCOs, with 86.4% complete and single-copy BUSCOs, 3.8% complete and duplicated BUSCOs, 3.3% fragmented BUSCOs, and 6.5% missing BUSCOs (Table 1). Next, we scaffolded the contigs using a proximity-ligation based short-read library [59]. The sequences from this library had a 94.71% mapping rate against the polished assembly. Scaffolding using HiRise v. 2.1.7, a software pipeline designed specifically for using proximity ligation data to scaffold genome assemblies [59], resulted in 12 scaffolds over 1 megabase pairs (Mb) in size and one scaffold just under 1Mb in size. These 13 scaffolds comprise 98.4% of the assembly, with a genome assembly scaffold N50 of 124Mb and BUSCO scores of 91.1% complete genes (Figure 2, Table 1). Genome assembly statistics were calculated using QUAST v. 5.0.2 (QUAST, RRID:SCR_001228) [60] applying default parameters, except setting the minimum contig length (--min-contig parameter) to 0. Previous studies have inferred the chromosome number of *A. bruennichi* to be 13, indicating our genome assembly is full-chromosome level [61,62]. As an additional assessment of assembly quality, we ran the K-mer Analysis Toolkit (KAT v. 2.4.2 , RRID: SCR_016741) [63] *comp* tool, comparing the k-mer content in the Illumina sequencing data to the k-mer content in the final assembly. Different values of the parameter *k* (*k* =17, 27, 29, 30 and 37) yielded k-mer completeness estimates ranging from 86.55 to 90.43% (Supplementary Figure S1). The missing k-mer content in the final assembly may be attributed to errors remaining in the assembly, likely within repeat

regions. This could be attributed to the moderate 19.8X coverage Illumina reads used for polishing and their short read length, which may have been insufficient to correct the more error-prone PacBio reads.

The 13 largest scaffolds are henceforth referred to as Chromosomes 1-13, ordered according to size (Figure 2B). The 14th-largest scaffold (Scaffold 839) contained the 16S sequence of a recently discovered, as yet unnamed, bacterial symbiont of *A. bruennichi* [48]. The remaining 2,217 scaffolds are much smaller, ranging from 1,747-258,743bp in length (Supplementary Figure S2) and will henceforth be referred to as “lesser scaffolds”.

Table 1: *Argiope bruennichi* genome assembly completeness

**Genome assembly statistic**	**Unscaffolded**	**Scaffolded**
**Assembly size (bp)**	1,669,116,561	1,670,285,661
**AT [a] / GC / N content (%)**	70.7 / 29.3 / 0	70.6 / 29.3 / 0.1
**Number of contigs / scaffolds**	13,843	2,231
**Longest contig / scaffold (bp)**	2,039,454	143,171,375
**Contig / scaffold N50 (bp)**	288,395	124,235,998
**Contig / scaffold N90 (bp)**	67,231	119,022,586
**% repetitive**	34.66	34.64
**BUSCO analysis [b]**		
Complete BUSCOs (%)	90.2	91.1
Complete and single-copy BUSCOs (%)	86.4	87.8
Complete and duplicated BUSCOs (%)	3.8	3.3
Fragmented BUSCOs (%)	3.3	2.8
Missing BUSCOs (%)	6.5	6.1

Genome assembly statistics were calculated using QUAST v. 5.0.2 (QUAST, RRID:SCR_001228) [60] using default parameters, except --min-contig 0.
[a] AT: adenine thymine
[b] BUSCO analysis using default parameters against the arthropod dataset

Repeat masking and removal of contaminants

The assembly was repeat-masked using a combination of the *de novo* repeat finder RepeatModeler (v. open-1.0.11) (RepeatModeler, RRID:SCR_015027) [64] and the homology-based repeat finder RepeatMasker (v. open-4.0.9) (RepeatMasker, RRID:SCR_012954) [65]. Repetitive regions accounted for 34.64% of the genome assembly, of which the majority (20.52% of the genome) consisted of unclassified repeats, meaning that they have not been classified in previous studies. The remaining repetitive elements were made up of DNA elements (i.e. transposable elements: 6.27%), long interspersed nuclear elements (LINEs: 1.60%), simple repeats (i.e. duplications of 1-5 bp: 1.58%), long terminal repeat (LTR) elements (0.76%), satellites (0.63%), low complexity repeats (i.e. poly-purine or poly-pyrimidine stretches: 0.42%), and short interspersed nuclear elements (SINEs: 0.08%) (Table 2). BlobTools (v. 1.0) (Blobtools, RRID:SCR_017618) [66] was used to search for contamination of bacterial or mitochondrial sequences, finding none.

Table 2: *Argiope bruennichi* repetitive DNA elements

Type of element	Number of elements	Length (bp)	Percentage of assembly
**SINEs**	4,643	1,314,740	0.08 %
**LINEs**	52,648	26,768,096	1.60 %
**LTR elements**	21,649	12,683,330	0.76 %
**DNA elements**	282,019	104,785,665	6.27 %
**Unclassified**	1,359,138	342,727,030	20.52 %
**Small RNA**	0	0	0.00 %
**Satellites**	28,474	10,495,658	0.63 %
**Simple repeats**	595,962	26,379,486	1.58 %
**Low complexity**	137,182	6,952,634	0.42 %
		**Total:**	34.64 %

Repetitive elements were classified using RepeatModeler (v. open-1.0.11) [64] and RepeatMasker (v. open-4.0.9) [65].

Genome annotation

Raw reads from previously published transcriptome sequencing data of different life stages: 20 pooled eggs (accession number SRR11861505), 20 pooled first instar spiderlings (accession number SRR11861504), one whole body of an adult female (accession number SRR11861502) and one whole body of an adult male (accession number SRR11861503) [5] were mapped against the repeat-masked assembly using HISAT2 (v. 2.1.0) (HISAT2, RRID:SCR_015530) [67]. After conversion of the resulting SAM file into a BAM file and subsequent sorting using SAMtools (v. 1.7) (SAMTOOLS, RRID:SCR_002105) [49], the sorted BAM file was converted to intron-hints for AUGUSTUS (v. 3.3.2) (Augustus, RRID:SCR_008417) [68] using AUGUSTUS scripts. AUGUSTUS was run on the soft-masked genome with the *Parasteatoda* parameter set. The resulting gff file containing predicted genes was converted into a gtf file using the AUGUSTUS script gtf2gff.pl. Additional AUGUSTUS scripts (getAnnoFastaFromJoinGenes.py and fix_in_frame_stop_codon_genes.py) were used to find and replace predicted protein-coding genes containing in-frame stop codons with newly predicted genes. The resulting gtf file containing 23,270 predicted genes (26,318 transcripts) was converted to gff3 format using gtf2gff.pl and protein sequences of predicted genes were extracted with getAnnoFastaFromJoinGenes.py. Finally, functional annotation was performed using InterProScan (v. 5.39-77.0) (InterProScan, RRID:SCR_005829) [69,70] (Table 3). The majority of annotated genes fall on the 13 chromosome scaffolds, although 272 transcripts were predicted on the lesser scaffolds.

The annotation gff3 file and the files containing predicted transcripts and proteins are available on GigaDB [71].

Table 3: *Argiope bruennichi* genome annotation statistics

Statistic	Value
**Number of protein coding genes**	23,270
**Functionally annotated genes (%)**	81.0
**Average exon length (bp)**	200
**Average intron length (bp)**	4,035
**BUSCO analysis** [a]	
Complete BUSCOs (%)	89.3
Complete and single-copy BUSCOs (%)	76.7
Complete and duplicated BUSCOs (%)	12.6
Fragmented BUSCOs (%)	7.0
Missing BUSCOs (%)	3.7

[a] BUSCO analysis using default parameters against the arthropod dataset

Comparative genomic analysis of repeat content

High repetitiveness is characteristic of spider genomes [27]. In order to compare the repeat content of *A. bruennichi* with that of other spiders, we downloaded the genome assemblies of several other spider species from NCBI and the DNA Data Bank of Japan (DDBJ) (accession numbers in Table 4), then treated them in the same manner as the *A. bruennichi* genome, masking the repeats using RepeatModeler (v. open-1.0.11) [64] and RepeatMasker (v. open-4.0.9) [65]. *Acanthoscurria geniculata* was excluded from this analysis due to the relatively poorly assembled genome. The *A. bruennichi* genome has a slightly lower percentage of repetitive element content (34.64%) compared to most other spiders (Table 4). Some species, such as *Loxosceles reclusa, Trichonephila clavipes* (formerly *Nephila clavipes*), *Anelosimus studiosus* and *Parasteatoda*

tepidariorum, have similar repetitive content (36.51%, 36.61%, 35.98% and 36.79% respectively); other species have much higher repetitive content, such as *Araneus ventricosus, Dysdera silvatica, Stegodyphus dumicola, Stegodyphus mimosarum* and *Pardosa pseudoannulata* (55.96%, 60.03%, 58.98%, 56.91% and 48.61% respectively). Only *Latrodectus hesperus* has lower repetitive content (20.97%). The classification and relative percentage of these repeats can be found in Supplementary Table S2 and Supplementary Figure S3. It is often asserted that the repeat content in spiders is higher in general than in other arthropod groups [i.e. 27]. In order to test this assertion, we looked into the repeat content in genomes of additional arthropod species. We obtained repeat content estimates, for which the repeats were masked using RepeatModeler and RepeatMasker, for three insect species (*Bombus terrestris, Drosophila melanogaster* and *Rhodnius prolixus* [72]), and seven tick and mite species (*Ixodes persulcatus, Haemaphysalis longicornis, Dermacentor silvarum, Hyalomma asiaticum, Rhipicephalus sanguineus, and Ixodes scapularus* [73]). We additionally downloaded the genomes of four more arthropod species, generated custom species-specific repeat libraries with RepeatModeler and masked the genomes with RepeatMasker, to avoid any issues of under- or over masking using other repeat masking programs: a butterfly, *Heliconius melpomene* [74], a beetle, *Tribolium castaneum* [75], a millipede, *Helicorthomorpha holstii* [76], and a scorpion, *Centruroides sculpturatus* [7,33]. The percentage of total repetitive content for all of these species is presented in Table 4. In general, spiders do have a higher repetitive content than insects, but there is a large range of repetitive content in spiders, compared to which the repetitive content in *A. bruennichi* is relatively low. All of the selected spider species, aside from *Latrodectus hesperus,* have higher repetitive

content than all other investigated groups, with the exception of ticks and mites, which have very high repetitive content overall (range: 52.6-64.4% repetitive). We conclude from this preliminary investigation that spider genomes, and arachnid genomes generally, do indeed have a higher repeat content than other arthropods.

Table 4: Total repetitive content in the genomes of spiders and selected other arthropods

Class	Order	Species	% repetitive	Accession number [reference]
Arachnida	Araneae	*Argiope bruennichi*	34.64	--
		Araneus ventricosus	55.96	BGPR01000001-BGPR01300721 [a] [29]
		Trichonephila clavipes	36.61	GCA_002102615.1 [b] [28]
		Dysdera silvatica	60.03	GCA_006491805.1 [b] [32]
		Stegodyphus dumicola	58.98	GCA_010614865.1 [b] [31]
		Stegodyphus mimosarum	56.91	GCA_000611955.2 [b] [27]
		Pardosa pseudoannulata	48.61	GCA_008065355.1 [b] [30]
		Loxosceles reclusa	36.51	GCA_001188405.1 [b] [33]
		Anelosimus studiosus	35.98	GCA_008297655.1 [b] [unpublished]
		Latrodectus hesperus	20.97	GCA_000697925.2 [b] [33]
		Parasteatoda tepidariorum	36.79	GCA_000365465.3 [b] [7]
	Scorpiones	*Centruroides sculpturatus*	34.4	GCA_000671375.2 [b] [26,31]
	Acari	*Ixodes persulcatus*	64.4	GCA_013358835.1 [b] [73]
		Haemaphysalis longicornis	59.3	GCA_013339765.1 [b] [73]
		Dermacentor silvarum	60.2	GCA_013339745.1 [b] [73]
		Hyalomma asiaticum	52.6	GCA_013339685.1 [b] [73]
		Rhipicephalus sanguineus	61.6	GCA_013339695.1 [b] [73]
		Rhipicephalus microplus	63.1	GCA_013339725.1 [b] [73]
		Ixodes scapularis	63.5	GCF_002892825.2 [b] [73,77]
Diplopoda	Helminthomorpha	*Helicorthomorpha holstii*	23.5	GCA_013389785.1 [b] [76]
Insecta	Hemiptera	*Rhodnius prolixus*	29.25	GCA_000181055.3 [b] [72]
	Hymenoptera	*Bombus terrestris*	12.51	GCA_000214255.1 [b] [72]
	Coleoptera	*Tribolium castaneum*	28.5	GCA_000002335.3 [b] [75]
	Lepidoptera	*Heliconius melpomene*	32.4	GCA_000313835.2 [b] [74]
	Diptera	*Drosophila melanogaster*	19.31	GCA_000001215.4 [b] [72]

Repetitive elements were classified using RepeatModeler (v. open-1.0.11) [64] and RepeatMasker (v. open-4.0.9) [65].

[a] DNA Data Bank of Japan (DDBJ)

[b] GenBank, National Center for Biotechnology Information (NCBI)

Genome architecture of Hox, spidroin and venom genes

Previous studies on spider genomes have focused on whole-genome duplication, silk gene evolution, and venom gene evolution [7,27–30]. Therefore, to place the *A. bruennichi* genome into the same context, we manually curated three gene sets from publicly available protein sequences: Hox, spidroin (silk), and venom genes. Because Hox genes are highly conserved across taxa [78], we chose the most complete sequences for the ten arthropod Hox gene classes from spiders without regard to the relatedness of the species to *A. bruennichi* (Supplementary File S1). In contrast to Hox genes, spidroin and venom genes are highly polymorphic and species-specific [79–82]. For the spidroin gene set, we downloaded protein sequences of the seven spidroin gene classes exclusively from five species of the genus *Argiope* (Supplementary File S2). Venom genes are best studied in spiders that are medically significant to humans, which are very distant relatives to *A. bruennichi* [51–54]. To allow comparison, we focused on venom gene sequences available for araneid spiders (two species, Supplementary File S3); however, the function and classification of these genes is poorly understood. With these three gene sets (Hox, spidroin, and venom), we performed a TBLASTN search against our genome assembly (v. 2.10.0+) (TBLASTN, RRID:SCR_011822) [87,88]. We recorded the genomic position of the best matches and compared them with the AUGUSTUS gene predictions for those locations. We employed a conservative E-value cutoff of less than 1.00×10^{-20} and only included results with an identity greater than 60%.

If hits overlapped on a scaffold or mapped to the same gene, only the hit with the highest identity and lowest E-value was retained. In cases where these metrics conflicted, the hit with the longest match length was retained. The manually curated FASTA files of each gene set used for the TBLASTN search are available in Supplementary Files S1-S3 and on GigaDB [71]. A table of the best matches with accession numbers for each gene set is available in Supplementary Tables S3-S5.

Hox cluster duplication

In 2017, Schwager *et al.* revealed that a whole-genome duplication (WGD) event occurred in the ancestor of scorpions and spiders, as evidenced by a high number of duplicated genes, including two clusters of Hox genes in the common house spider *Parasteatoda tepidariorum* and the bark scorpion *Centruroides sculpturatus* [7]. They found one nearly-complete cluster of Hox genes on a single scaffold, lacking the *fushi tarazu (ftz)* gene, which they argued may be the case for this cluster in all spiders. The second set of Hox genes was distributed across two scaffolds, which the authors attributed to incompleteness of the assembly due to patchy sequencing coverage [7]. For consistency, we will use the same nomenclature for Hox genes as used in [7] (*Abdominal-B: AbdB, Abdominal-A: AbdA, Ultrabithorax: Ubx, Antennapedia: Antp, fushi tarazu: ftz, sex combs reduced: scr, Deformed: Dfd, Hox3, proboscipedia: pb, labial: lab*). Corresponding with the results from *P. tepidariorum*, we found two clusters of Hox genes in *A. bruennichi*, with no evidence of tandem duplication. The two clusters occurred on two chromosomes (Chromosome 6 and Chromosome 9). In these locations, InterProScan generally annotated the genes as Hox genes but did not identify the specific type. On Chromosome 9, the Hox genes were in reverse colinear order (ordered according to their

expression in development), with no overlapping regions. Because the cluster on Chromosome 9 is complete, we will refer to it as "Cluster A." On Chromosome 6, ("Cluster B") the genes were out of colinear order, with the position of *AbdA* and *Ubx* switched, and the coordinates for *Dfd, Hox3* and *pb* from the blast search overlapping (Figure 3A). The hits for *Antp* and *ftz* in Cluster B fell onto a single predicted gene in the annotation. Thus, it is unclear if *A. bruennichi* lacks one copy of *ftz*, as in *P. tepidariorum*, or if the annotation incorrectly fused the two genes in this cluster. In the study by Schwager *et al.* [7], low sequencing coverage of Cluster B downstream of *Dfd* limited their inference. In our genome assembly, by mapping the PacBio reads against the final assembly, we calculated that we have an average of more than 12X coverage across the length of both clusters, suggesting that Cluster B is not out of order due to problems arising from low coverage. It is possible that Hox Cluster B in spiders has changed or lost functionality following the proposed ancestral WGD event. To check if the two Hox-containing chromosomes show evidence of duplication, we performed an analysis of conserved synteny using the tool SatsumaSynteny2 (https://github.com/bioinfologics/satsuma2). "Synteny" here refers to loci occurring on the same chromosome; chromosomes with conserved synteny will have a high degree of syntenic blocks in common. In the genome of *A. bruennichi*, Chromosomes 6 and 9 show a high level of conserved synteny (Figure 3B). The presence of two Hox clusters on highly syntenic chromosomes in our assembly is suggestive, but not evidence, of WGD in *A. bruennichi,* as it could have also arisen from duplication of only the ancestral Hox-containing chromosome; future studies will be able to capitalize on the now-available chromosome-level assemblies for several groups

(e.g. horseshoe crabs, ticks, and our spider) [73,89] to do more detailed analyses of duplication across chelicerates.

Spidroin genes

There are seven classes of silk produced by araneomorph spiders, each with one or more unique uses; it is important to note that the uses of these silk types are best understood for spiders in the family Araneidae, and the number and uses of silk types can vary widely between families [28,29,90,91]. The classes of silk are major ampullate (*MaSp*) minor ampullate (*MiSp*), piriform (*PiSp*), aggregate (*AgSp*), aciniform (*AcSp*) tubuliform (also referred to as cylindrical) (*TuSp*) and flagelliform (*Flag*). In *A. bruennichi*, spidroin genes occur on eight out of the thirteen chromosome scaffolds (Chromosomes 1, 3, 4, 6, 8, 11, 12 and 13) (Figure 4). There were no hits on the lesser scaffolds. We found four unique hits for *AcSp,* six hits for *AgSp,* one hit for *Flag,* eleven hits for *MaSp,* three hits for *MiSp,* one hit for *PiSp* and four hits for *TuSp.* In the majority of cases, all blast hits for a single spidroin type occurred on a single chromosome; the only exception was for *AgSp*, which had hits on four different chromosomes. However, these were not all annotated as spidroins; on Chromosome 6 there were two *AgSp* hits which were annotated as spidroins and one hit which was annotated as a chitin-binding domain, while on Chromosome 4 the *AgSp* hit was annotated as tropoelastin, on Chromosome 3 the hit was annotated as a chitin-binding domain, and on Chromosome 8 the hit was annotated as a serine protease. All hits for *TuSp* occurred on Chromosome 1, but there were hits in two physically separated areas of the chromosome; in one region there were hits on three annotated genes, and only one hit in the other region. There are more sequences available on NCBI for *MaSp* than any of the other spidroin types in the genus *Argiope*, which allowed us to

find matches for several unique *MaSp* genes in the *A. bruennichi* assembly. These occur in a small region of Chromosome 12, in close proximity to one another, suggesting that the spidroin genes in this group may have diversified via tandem duplication, as has been suggested in previous studies [92].

Venom genes

We found high identity matches for venom toxins on five of the chromosome scaffolds (Chromosomes 1, 2, 7, 10 and 11) (Figure 4), but the majority of hits were on Chromosome 1. In most cases, each region containing venom gene matches contained only one gene, with the exception of a region on Chromosome 1, which contained five genes in very close proximity to one another, and two other regions (on Chromosome 1 and Chromosome 11), which contained matches to two genes. Babb *et al.* 2017 [28] conducted a study on silk genes in *Trichonephila clavipes* (formerly *Nephila clavipes*), in which they found a novel flagelliform-type gene (FLAG-b) which was expressed most highly in the venom glands, not the flagelliform silk glands. This added to previous findings in the *Stegodyphus mimosarum* genome, where spidroin-like proteins are also found in the venom glands [27]. Interestingly, in the *A. bruennichi* genome assembly, there are several venom genes on Chromosome 11 in close proximity to the flagelliform spidroin gene.

Conclusions

We have assembled and annotated the first chromosome-level genome for a spider. The assembly approach of combining long read, short read, and proximity ligation data overcame the challenges of assembling arachnid genomes, namely large genome size, high repetitiveness, and low GC content. In our study, we made a preliminary analysis of

the location of certain gene families of interest in the context of spider genomics, which hinted at several interesting directions for future studies on the evolution of silk and venom genes. Furthermore, because this species has undergone a recent and rapid range expansion, the well-resolved genome assembly will be useful for studies on the genomic underpinnings of range expansion and evolutionary adaptation to novel climates.

Availability of supporting data

The final genome assembly and raw data from the PacBio and Hi-C libraries, as well as the annotation, have been deposited at NCBI under BioProject PRJNA629526. A publicly accessible genome browser hub with the annotation, raw transcriptome, and PacBio read coverage can be found on the UCSC Genome Browser server (under “My Data” > “Track Hubs” > “My Hubs” enter the URL http://bioinf.uni-greifswald.de/hubs/argiope/hub.txt). Supporting data is available via the GigaScience data repository, GigaDB, including the softmasked assembly in FASTA format, the output file from RepeatMasker, predicted coding genes and their functional annotation in GFF3 formats, predicted coding gene nucleotide and translated sequences in FASTA formats, functional annotation from InterProScan in TSV format, the blast query results for Hox, spidroin and venom genes in FASTA format, and the BUSCO output files in a zip folder [71].

Declarations

List of abbreviations

Abd-A: Abdominal-A; Abd-B: Abdominal-B; AcSp: aciniform spidroin; *AgSp:* aggregate spidroin; *Antp: Antennapedia;* AT: adenine thymine; bp: basepairs; BUSCO: Benchmarking Universal Single Copy Orthologs; DDBJ: DNA Data Bank of Japan; *Dfd: Deformed;* DNA: deoxyribonucleic acid; *Flag:* flagelliform spidroin; *ftz: fushi tarazu;* Gb: gigabase pairs; GC: guanine cytosine; kb: kilobase pairs; *lab: labial;* LINE: long interspersed nuclear element; LTR: long terminal repeat; *MaSp:* major ampullate spidroin; Mb: megabase pairs; *MiSp:* minor ampullate spidroin; NCBI: National Center for Biotechnology Information; PacBio: Pacific Biosciences; *pb: proboscipedia;* PCR: polymerase chain reaction; *PiSp:* piriform spidroin; RNA: ribonucleic acid; *scr: sex combs reduced;* SINE: short interspersed nuclear element; *TuSp:* tubuliform spidroin; *Ubx: Ultrabithorax;* WGD: whole-genome duplication

Consent for publication

Not applicable.

Competing interests

The authors declare that they have no competing interests.

Funding

Funding for this study was provided by the Deutsche Forschungsgemeinschaft (DFG) as part of the Research Training Group 2010 RESPONSE (GRK 2010) to GU.

Authors' contributions

MMS, HK, GU, and SP conceived of the study; MMS, HK, and GU collected the spiders. HK extracted DNA for the PacBio sequencing; MMS prepared and submitted the DNA for PacBio sequencing, with input and infrastructure provided by RGG. MMS and CJ

constructed and sequenced the Hi-C library, with input and infrastructure provided by LJ and AWK. MMS, AH and SP performed the genome assembly, and AH and KJH performed the genome annotation with input and infrastructure provided by MMS and SP. AH and KJH analyzed the repeat content of other arthropod species; MMS performed the analysis of Hox duplication, spidroin genes, and venom genes. MMS, AH, KJH and SP wrote the first draft of the manuscript. All authors read and approved the final manuscript.

Acknowledgements

We would like to thank the California Academy of Sciences for allowing us access to their computing resources for the genome assembly, and to Dovetail Genomics for their support in troubleshooting the Hi-C kit and running HiRise. MMS thanks José Cerca for helpful ideas and discussions about the silk and venom gene analysis.

References

1. Wise DH. Spiders in Ecological Webs. Cambridge: Cambridge University Press; 1993.

2. Spiller DA, Schoener TW. Effects of top and intermediate predators in a terrestrial food web. Ecology. Ecological Society of America; 1994;75:182–96.

3. Moulder BC, Reichle DE. Significance of spider predation in the energy dynamics of forest-floor arthropod communities. Ecol Monogr. Wiley; 1972;42:473–98.

4. Wirta HK, Weingartner E, Hambäck PA, Roslin T. Extensive niche overlap among the dominant arthropod predators of the High Arctic. Basic Appl Ecol. Elsevier GmbH; 2015;16:86–92.

5. Krehenwinkel H, Rödder D, Tautz D. Eco-genomic analysis of the poleward range

expansion of the wasp spider Argiope bruennichi shows rapid adaptation and genomic admixture. Glob Chang Biol. John Wiley & Sons, Ltd; 2015;21:4320–32.

6. Garb JE, González A, Gillespie RG. The black widow spider genus *Latrodectus* (Araneae: Theridiidae): Phylogeny, biogeography, and invasion history. Mol Phylogenet Evol. Academic Press Inc.; 2004;31:1127–42.

7. Schwager EE, Sharma PP, Clarke T, Leite DJ, Wierschin T, Pechmann M, et al. The house spider genome reveals an ancient whole-genome duplication during arachnid evolution. BMC Biol. BMC Biology; 2017;15:1–27.

8. Clarke TH, Garb JE, Hayashi CY, Arensburger P, Ayoub NA. Spider transcriptomes identify ancient large-scale gene duplication event potentially important in silk gland evolution. Genome Biol Evol. Oxford University Press; 2015;7:1856–70.

9. Gendreau KL, Haney RA, Schwager EE, Wierschin T, Stanke M, Richards S, et al. House spider genome uncovers evolutionary shifts in the diversity and expression of black widow venom proteins associated with extreme toxicity. BMC Genomics. BMC Genomics; 2017;18:178.

10. Carson HL, Clayton FE, Stalker HD. Karyotypic stability and speciation in Hawaiian *Drosophila.* Proc Natl Acad Sci U S A. National Academy of Sciences; 1967;57:1280–5.

11. Řezáč M, Arnedo MA, Opatova V, Musilová J, Řezáčová V, Král J. Taxonomic revision and insights into the speciation mode of the spider *Dysdera erythrina* species-complex (Araneae: Dysderidae): Sibling species with sympatric distributions. Invertebr Syst. CSIRO; 2018;32:10–54.

12. Mérot C, Oomen RA, Tigano A, Wellenreuther M. A roadmap for understanding the

evolutionary significance of structural genomic variation. Trends Ecol Evol. Elsevier Ltd; 2020;35:561–72.

13. Shchur V, Svedberg J, Medina P, Corbett-Detig R, Nielsen R. On the distribution of tract lengths during adaptive introgression. G3 Genes, Genomes, Genet. Genetics Society of America; 2020;10:3663–73.

14. Fuller ZL, Koury SA, Phadnis N, Schaeffer SW. How chromosomal rearrangements shape adaptation and speciation: Case studies in *Drosophila pseudoobscura* and its sibling species *Drosophila persimilis*. Mol Ecol. Blackwell Publishing Ltd; 2019;28:1283–301.

15. Faria R, Navarro A. Chromosomal speciation revisited: Rearranging theory with pieces of evidence. Trends Ecol Evol. Elsevier Current Trends; 2010;25:660–9.

16. White MJD. Chromosomal rearrangements and speciation in animals. Annu Rev Genet. Annual Reviews; 1969;3:75–98.

17. Rieseberg LH. Chromosomal rearrangements and speciation. Trends Ecol Evol. Elsevier Current Trends; 2001;16:351–8.

18. Noor MAF, Gratos KL, Bertucci LA, Reiland J. Chromosomal inversions and the reproductive isolation of species. Proc Natl Acad Sci U S A. National Academy of Sciences; 2001;98:12084–8.

19. Yannic G, Basset P, Hausser J. Chromosomal rearrangements and gene flow over time in an inter-specific hybrid zone of the *Sorex araneus* group. Heredity (Edinb). Nature Publishing Group; 2009;102:616–25.

20. Feulner PGD, De-Kayne R. Genome evolution, structural rearrangements and

speciation. Artic J Evol Biol. John Wiley & Sons, Ltd; 2017;30:1488–90.

21. Castiglia R. Sympatric sister species in rodents are more chromosomally differentiated than allopatric ones: Implications for the role of chromosomal rearrangements in speciation. Mamm Rev. Blackwell Publishing Ltd; 2014;44:1–4.

22. Wellenreuther M, Mérot C, Berdan E, Bernatchez L. Going beyond SNPs: The role of structural genomic variants in adaptive evolution and species diversification. Mol Ecol. Blackwell Publishing Ltd; 2019;28:1203–9.

23. Vijay N, Bossu CM, Poelstra JW, Weissensteiner MH, Suh A, Kryukov AP, et al. Evolution of heterogeneous genome differentiation across multiple contact zones in a crow species complex. Nat Commun. Nature Publishing Group; 2016;7:1–10.

24. Turner TL, Hahn MW, Nuzhdin S V. Genomic islands of speciation in *Anopheles gambiae*. PLoS Biol. Public Library of Science; 2005;3:1572–8.

25. Hejase HA, Salman-Minkov A, Campagna L, Hubisz MJ, Lovette IJ, Gronau I, et al. Genomic islands of differentiation in a rapid avian radiation have been driven by recent selective sweeps. bioRxiv. Cold Spring Harbor Laboratory; 2020;2020.03.07.977694.

26. Duranton M, Allal F, Fraïsse C, Bierne N, Bonhomme F, Gagnaire PA. The origin and remolding of genomic islands of differentiation in the European sea bass. Nat Commun. Nature Publishing Group; 2018;9:1–11.

27. Sanggaard KW, Bechsgaard JS, Fang X, Duan J, Dyrlund TF, Gupta V, et al. Spider genomes provide insight into composition and evolution of venom and silk. Nat Commun. Nature Publishing Group; 2014;5:3765.

28. Babb PL, Lahens NF, Correa-Garhwal SM, Nicholson DN, Kim EJ, Hogenesch JB,

et al. The *Nephila clavipes* genome highlights the diversity of spider silk genes and their complex expression. Nat Genet. Nature Publishing Group; 2017;49:895–903.

29. Kono N, Nakamura H, Ohtoshi R, Moran DAP, Shinohara A, Yoshida Y, et al. Orb-weaving spider *Araneus ventricosus* genome elucidates the spidroin gene catalogue. Sci Rep. Nature Publishing Group; 2019;9:8380.

30. Yu N, Li J, Liu M, Huang L, Bao H, Yang Z, et al. Genome sequencing and neurotoxin diversity of a wandering spider *Pardosa pseudoannulata* (pond wolf spider). bioRxiv. 2019;747147.

31. Liu S, Aagaard A, Bechsgaard J, Bilde T. DNA methylation patterns in the social spider, *Stegodyphus dumicola*. Genes (Basel). MDPI; 2019;10:137.

32. Sánchez-Herrero JF, Frías-López C, Escuer P, Hinojosa-Alvarez S, Arnedo MA, Sánchez-Gracia A, et al. The draft genome sequence of the spider *Dysdera silvatica* (Araneae, Dysderidae): A valuable resource for functional and evolutionary genomic studies in chelicerates. Gigascience. Oxford University Press; 2019;8:giz099.

33. Thomas GWC, Dohmen E, Hughes DST, Murali SC, Poelchau M, Glastad K, et al. Gene content evolution in the arthropods. Genome Biol. BioMed Central Ltd.; 2020;21:15.

34. Stellwagen SD, Renberg RL. Toward spider glue: Long read scaffolding for extreme length and repetitious silk family genes AgSp1 and AgSp2 with insights into functional adaptation. G3 Genes, Genomes, Genet. Genetics Society of America; 2019;9:1909–19.

35. Ayoub NA, Garb JE, Kuelbs A, Hayashi CY. Ancient properties of spider silks

revealed by the complete gene sequence of the prey-wrapping silk protein (AcSp1). Mol Biol Evol. Oxford University Press; 2013;30:589–601.

36. Krehenwinkel H, Tautz D. Northern range expansion of European populations of the wasp spider Argiope bruennichi is associated with global warming-correlated genetic admixture and population-specific temperature adaptations. Mol Ecol. Blackwell Publishing Ltd; 2013;22:2232–48.

37. Wawer W, Rutkowski R, Krehenwinkel H, Lutyk D, Pusz- K. Population structure of the expansive wasp spider (*Argiope bruennichi*) at the edge of its range. J Arachnol. American Arachnological Society; 2017;45:361–9.

38. Krehenwinkel H, Graze M, Rödder D, Tanaka K, Baba YG, Muster C, et al. A phylogeographical survey of a highly dispersive spider reveals eastern Asia as a major glacial refugium for Palaearctic fauna. J Biogeogr. John Wiley & Sons, Ltd; 2016;43:1583–94.

39. Wolz M, Klockmann M, Schmitz T, Pekár S, Bonte D, Uhl G. Dispersal and life-history traits in a spider with rapid range expansion. Mov Ecol 2019 81. BioMed Central; 2020;8:1–11.

40. Fromhage L, Uhl G, Schneider JM. Fitness consequences of sexual cannibalism in female *Argiope bruennichi*. Behav Ecol Sociobiol. Springer-Verlag; 2003;55:60–4.

41. Schneider JM, Fromhage L, Uhl G. Extremely short copulations do not affect hatching success in *Argiope bruennichi* (Araneae, Araneidae). J Arachnol. American Arachnological Society; 2005;33:663–9.

42. Schneider J, Uhl G, Herberstein ME. Cryptic female choice within the genus

Argiope: A comparative approach. In: Peretti A, Aisenberg A, editors. Cryptic Female Choice Arthropods Patterns, Mech Prospect. Cham: Springer International Publishing; 2015. p. 55–77.

43. Chinta SP, Goller S, Lux J, Funke S, Uhl G, Schulz S. The sex pheromone of the wasp spider *Argiope bruennichi*. Angew Chemie - Int Ed. Wiley-Blackwell; 2010;49:2033–6.

44. Uhl G, Zimmer SM, Renner D, Schneider JM. Exploiting a moment of weakness: male spiders escape sexual cannibalism by copulating with moulting females. Sci Rep. Nature Publishing Group; 2015;5:16928.

45. Gregory TR, Shorthouse DP. Genome sizes of spiders. J Hered. Oxford Academic; 2003;94:285–90.

46. Rasch EM, Connelly BA. Genome size and endonuclear DNA replication in spiders. J Morphol. John Wiley & Sons, Ltd; 2005;265:209–14.

47. Gregory TR. Animal Genome Size Database [Internet]. 2020. Available from: http://www.genomesize.com/index.php

48. Schell T, Feldmeyer B, Schmidt H, Greshake B, Tills O, Truebano M, et al. An annotated draft genome for *Radix auricularia* (Gastropoda, Mollusca). Genome Biol Evol. Oxford University Press (OUP); 2017;9:585–92.

49. Li H, Handsaker B, Wysoker A, Fennell T, Ruan J, Homer N, et al. The Sequence Alignment/Map format and SAMtools. Bioinformatics. Oxford University Press; 2009;25:2078–9.

50. Li H. Aligning sequence reads, clone sequences and assembly contigs with BWA-

MEM. 2013;

51. Okonechnikov K, Conesa A, García-Alcalde F. Qualimap 2: Advanced multi-sample quality control for high-throughput sequencing data. Bioinformatics. Oxford University Press; 2016;32:292–4.

52. R Core Team. R: A language and environment for statistical computing. Vienna, Austria: R Foundation for Statistical Computing; 2017.

53. Ewels P, Magnusson M, Lundin S, Käller M. MultiQC: Summarize analysis results for multiple tools and samples in a single report. Bioinformatics. Oxford University Press; 2016;32:3047–8.

54. Quinlan AR, Hall IM. BEDTools: A flexible suite of utilities for comparing genomic features. Bioinformatics. Oxford University Press; 2010;26:841–2.

55. Li H. Minimap2: Pairwise alignment for nucleotide sequences. Bioinformatics. Oxford University Press; 2018;34:3094–100.

56. Ruan J, Li H. Fast and accurate long-read assembly with wtdbg2. Nat Methods. Nature Research; 2020;17:155–8.

57. Walker BJ, Abeel T, Shea T, Priest M, Abouelliel A, Sakthikumar S, et al. Pilon: An integrated tool for comprehensive microbial variant detection and genome assembly improvement. PLoS One. Public Library of Science; 2014;9:e112963.

58. Simão FA, Waterhouse RM, Ioannidis P, Kriventseva E V., Zdobnov EM. BUSCO: Assessing genome assembly and annotation completeness with single-copy orthologs. Bioinformatics. Oxford University Press; 2015;31:3210–2.

59. Putnam NH, O'Connell BL, Stites JC, Rice BJ, Blanchette M, Calef R, et al.

Chromosome-scale shotgun assembly using an in vitro method for long-range linkage. Genome Res. Cold Spring Harbor Laboratory Press; 2016;26:342–50.

60. Gurevich A, Saveliev V, Vyahhi N, Tesler G. QUAST: Quality assessment tool for genome assemblies. Bioinformatics. Oxford University Press; 2013;29:1072–5.

61. Zhang YJ, Tong SJ. The routine method for preparing the chromosomes in spiders. Chinese J Zool. 1990;25:30–1.

62. Araujo D, Mattos VF, Giroti AM, Kraeski MG, Carvalho LS, Brescovit AD. Cytogenetical characterization of six orb-weaver species and review of cytogenetical data for Araneidae. J Arachnol. American Arachnological Society; 2011;39:337–44.

63. Mapleson D, Accinelli GG, Kettleborough G, Wright J, Clavijo BJ. KAT: A K-mer analysis toolkit to quality control NGS datasets and genome assemblies. Bioinformatics. Oxford University Press; 2017;33:574–6.

64. Smit AFA, Hubley R. RepeatModeler-1.0. 2008.

65. Smit AFA, Hubley R. RepeatMasker-4.0. 2013.

66. Laetsch DR, Blaxter ML. BlobTools: Interrogation of genome assemblies. F1000Research. F1000 Research, Ltd.; 2017;6:1287.

67. Kim D, Paggi JM, Park C, Bennett C, Salzberg SL. Graph-based genome alignment and genotyping with HISAT2 and HISAT-genotype. Nat Biotechnol. Nature Publishing Group; 2019;37:907–15.

68. Hoff KJ, Stanke M. Predicting genes in single genomes with AUGUSTUS. Curr Protoc Bioinforma. John Wiley & Sons, Ltd; 2019;65:e57.

69. Jones P, Binns D, Chang HY, Fraser M, Li W, McAnulla C, et al. InterProScan 5: Genome-scale protein function classification. Bioinformatics. Oxford University Press; 2014;30:1236–40.

70. Quevillon E, Silventoinen V, Pillai S, Harte N, Mulder N, Apweiler R, et al. InterProScan: Protein domains identifier. Nucleic Acids Res. Oxford University Press; 2005;33:W116–20.

71. Sheffer MM, Hoppe A, Krehenwinkel H, Uhl G, Kuss AW, Jensen L, et al. Supporting data for "Chromosome-level reference genome of the European wasp spider *Argiope bruennichi*: a resource for studies on range expansion and evolutionary adaptation." GigaScience Database 2020. http://dx.doi.org/10.5524/100837

72. Brůna T, Hoff KJ, Lomsadze A, Stanke M, Borodovsky M. BRAKER2: Automatic eukaryotic genome annotation with GeneMark-EP+ and AUGUSTUS supported by a protein database. bioRxiv. 2020;

73. Jia N, Wang J, Shi W, Du L, Sun Y, Zhan W, et al. Large-scale comparative analyses of tick genomes elucidate their genetic diversity and vector capacities. Cell. Cell Press; 2020;1–13.

74. Dasmahapatra KK, Walters JR, Briscoe AD, Davey JW, Whibley A, Nadeau NJ, et al. Butterfly genome reveals promiscuous exchange of mimicry adaptations among species. Nature. Nature Publishing Group; 2012;487:94–8.

75. Kim HS, Murphy T, Xia J, Caragea D, Park Y, Beeman RW, et al. BeetleBase in 2010: Revisions to provide comprehensive genomic information for *Tribolium castaneum*. Nucleic Acids Res. Oxford University Press; 2009;38:D437.

76. Qu Z, Nong W, So WL, Barton-Owen T, Li Y, Li C, et al. Millipede genomes reveal unique adaptation of genes and microRNAs during myriapod evolution. bioRxiv. Cold Spring Harbor Laboratory; 2020;2020.01.09.900019.

77. Miller JR, Koren S, Dilley KA, Harkins DA, Stockwell TB, Shabman RS, et al. A draft genome sequence for the *Ixodes scapularis* cell line, ISE6. F1000Research. F1000 Research Ltd; 2018;7:297.

78. Pearson JC, Lemons D, McGinnis W. Modulating Hox gene functions during animal body patterning. Nat Rev Genet. Nature Publishing Group; 2005;6:893–904.

79. Gatesy J, Hayashi C, Motriuk D, Woods J, Lewis R. Extreme diversity, conservation, and convergence of spider silk fibroin sequences. Science (80-). American Association for the Advancement of Science; 2001;291:2603–5.

80. Hayashi CY, Shipley NH, Lewis R V. Hypotheses that correlate the sequence, structure, and mechanical properties of spider silk proteins. Int J Biol Macromol. Elsevier; 1999;24:271–5.

81. Casewell NR, Wü Ster W, Vonk FJ, Harrison RA, Fry BG. Complex cocktails: the evolutionary novelty of venoms. Trends Ecol Evol. 2013;28:219–29.

82. Fry BG, Roelants K, Champagne DE, Scheib H, Tyndall JDA, King GF, et al. The toxicogenomic multiverse: Convergent recruitment of proteins into animal venoms. Annu Rev Genomics Hum Genet. 2009;10:483–511.

83. Grishin E. Polypeptide neurotoxins from spider venoms. Eur J Biochem. John Wiley & Sons, Ltd; 1999;264:276–80.

84. Escoubas P. Molecular diversification in spider venoms: A web of combinatorial

peptide libraries. Mol Divers. 2006;10:545–54.

85. Escoubas P, Sollod B, King GF. Venom landscapes: Mining the complexity of spider venoms via a combined cDNA and mass spectrometric approach. Toxicon. Pergamon; 2006;47:650–63.

86. Diniz CR, do Nascimento Cordeiro M, Junor LR, Kelly P, Fischer S, Reimann F, et al. The purification and amino acid sequence of the lethal neurotoxin Tx1 from the venom of the Brazilian 'armed' spider *Phoneutria nigriventer*. FEBS Lett. 1990;263:251–3.

87. Gerts EM, Yu YK, Agarwala R, Schäffer AA, Altschul SF. Composition-based statistics and translated nucleotide searches: Improving the TBLASTN module of BLAST. BMC Biol. BioMed Central; 2006;4:41.

88. Altschul SF, Gish W, Miller W, Myers EW, Lipman DJ. Basic local alignment search tool. J Mol Biol. Academic Press; 1990;215:403–10.

89. Shingate P, Ravi V, Prasad A, Tay BH, Garg KM, Chattopadhyay B, et al. Chromosome-level assembly of the horseshoe crab genome provides insights into its genome evolution. Nat Commun. Springer US; 2020;11:2322.

90. Vollrath F. Biology of spider silk. Int J Biol Macromol. 1999;24:81–8.

91. Blackledge TA, Hayashi CY. Silken toolkits: Biomechanics of silk fibers spun by the orb web spider *Argiope argentata* (Fabricius 1775). J Exp Biol. The Company of Biologists Ltd; 2006;209:2452–61.

92. Zhao Y, Ayoub NA, Hayashi CY. Chromosome mapping of dragline silk genes in the genomes of widow spiders (Araneae, Theridiidae). PLoS One. 2010;5:e12804.

93. Durand NC, Robinson JT, Shamim MS, Machol I, Mesirov JP, Lander ES, et al. Juicebox provides a visualization system for Hi-C contact maps with unlimited zoom. Cell Syst. Cell Press; 2016;3:99–101.

94. Krzywinski M, Schein J, Birol I, Connors J, Gascoyne R, Horsman D, et al. Circos: An information aesthetic for comparative genomics. Genome Res. 2009;19:1639–45.

Figure Legends

Figure 1: Female *Argiope bruennichi* spider in orb web from Loulé (Faro, Portugal). Photograph by Monica M. Sheffer.

Figure 2: *Argiope bruennichi* genome assembly completeness. (A) Contact heatmap of Hi-C scaffolding shows long-range contacts of paired-end Hi-C reads. Gray gridlines denote scaffold (chromosome) boundaries. Visualized with Juicebox (v. 1.11.08) [93]. (B) The length of the 20 longest scaffolds in the assembly shows that the 13 putative chromosome scaffolds are much larger than the next largest. Red points represent individual scaffolds, ordered from largest to smallest. (C) Cumulative length of assembly contained within scaffolds. Note that the vast majority (98.4%) of the genome is contained within very few scaffolds. Visualized with QUAST v. 5.0.2 [60] using default parameters, except --min-contig 0.

Figure 3: Duplication of the Hox-containing chromosomes. (A) Hox gene clusters. Genes connected by a black line occur on the same scaffold, in the order depicted. Cluster A occurs on Chromosome 9, and Cluster B occurs on Chromosome 6. (B) A synteny plot of the results of SatsumaSynteny2 (https://github.com/bioinfologics/satsuma2) visualized in Circos [94] shows chromosome-scale conservation of synteny for the Hox-containing chromosomes (Chromosomes 6 and 9). The two curved rectangles represent Chromosomes 6 and 9, and the tick marks represent the position on the chromosome, in megabase pairs. Lines between the two rectangles show the shared syntenic blocks between the chromosomes, based on sequence homology. The presence of two Hox gene clusters

on two highly syntenic chromosomes is suggestive of whole-genome duplication in *Argiope bruennichi*, as was found previously for *Parasteatoda tepidariorum* [7].

Figure 4: Schematic representation of the location of gene families on the 13 chromosomes. The light grey bars represent chromosomes, the colored rectangles represent the seven different spidroin gene families, the black rectangles represent venom genes, and the white rectangles represent Hox gene clusters. The numbers inside of the rectangles represent the number of genes found within that cluster.

Figure 1

Click here to access/download;Figure;Figure1_Argiope_bruennichi_MertolaPo

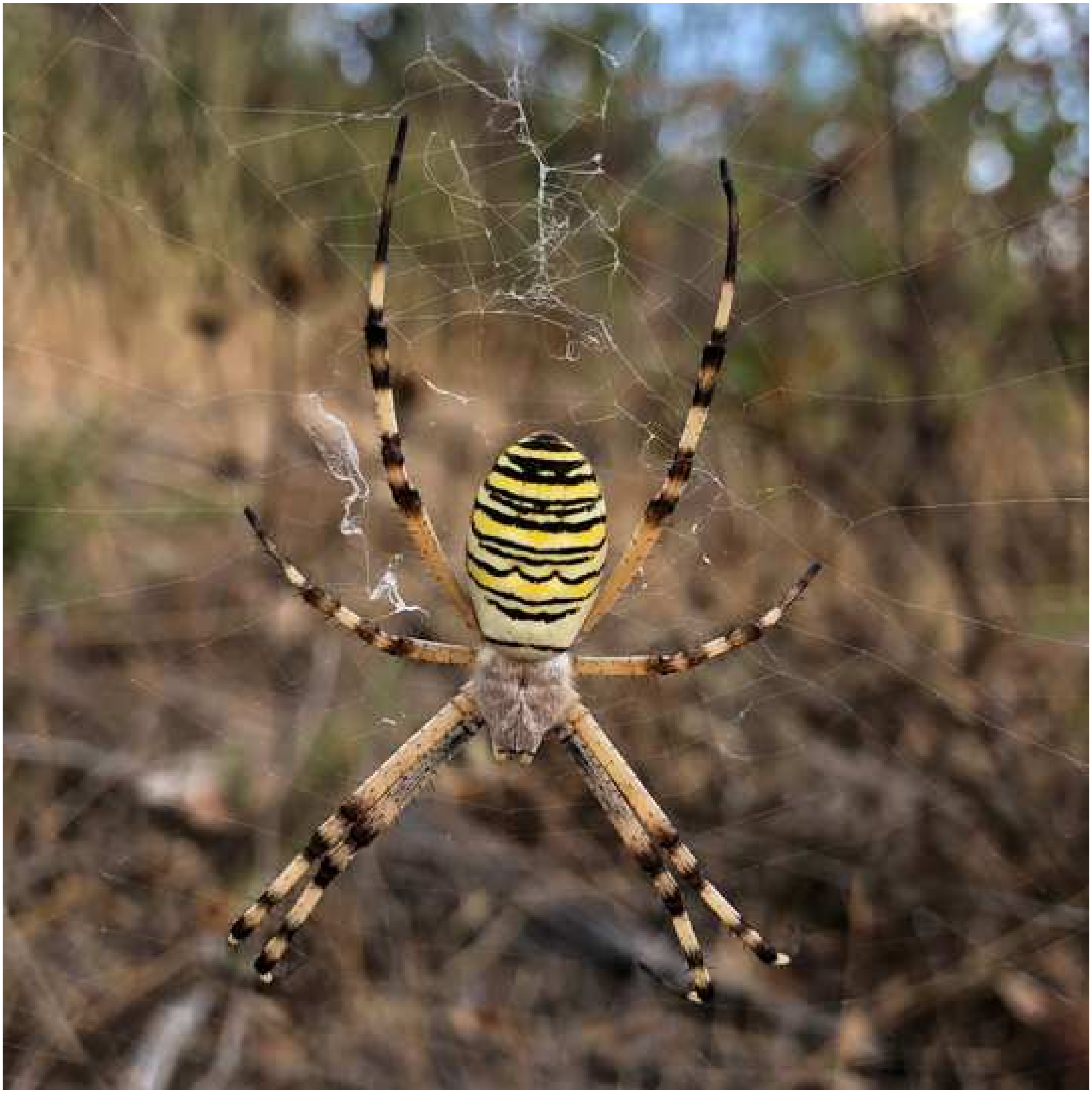

Figure 2

Click here to access/download;Figure;Figure2_assembly_figur

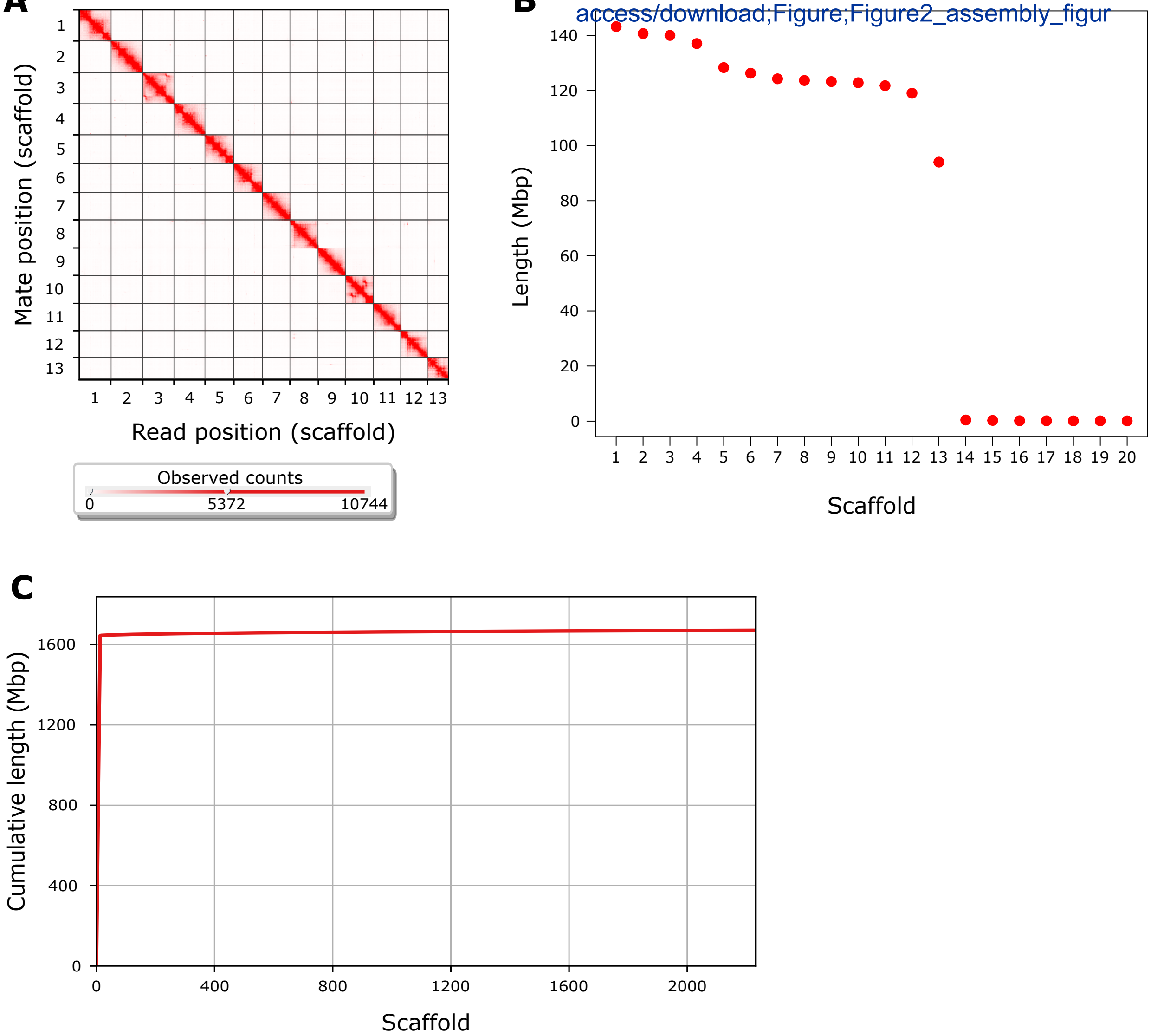

Figure 3

Click here to access/download;Figure;Figure3_New_HoxDuplication.jpg

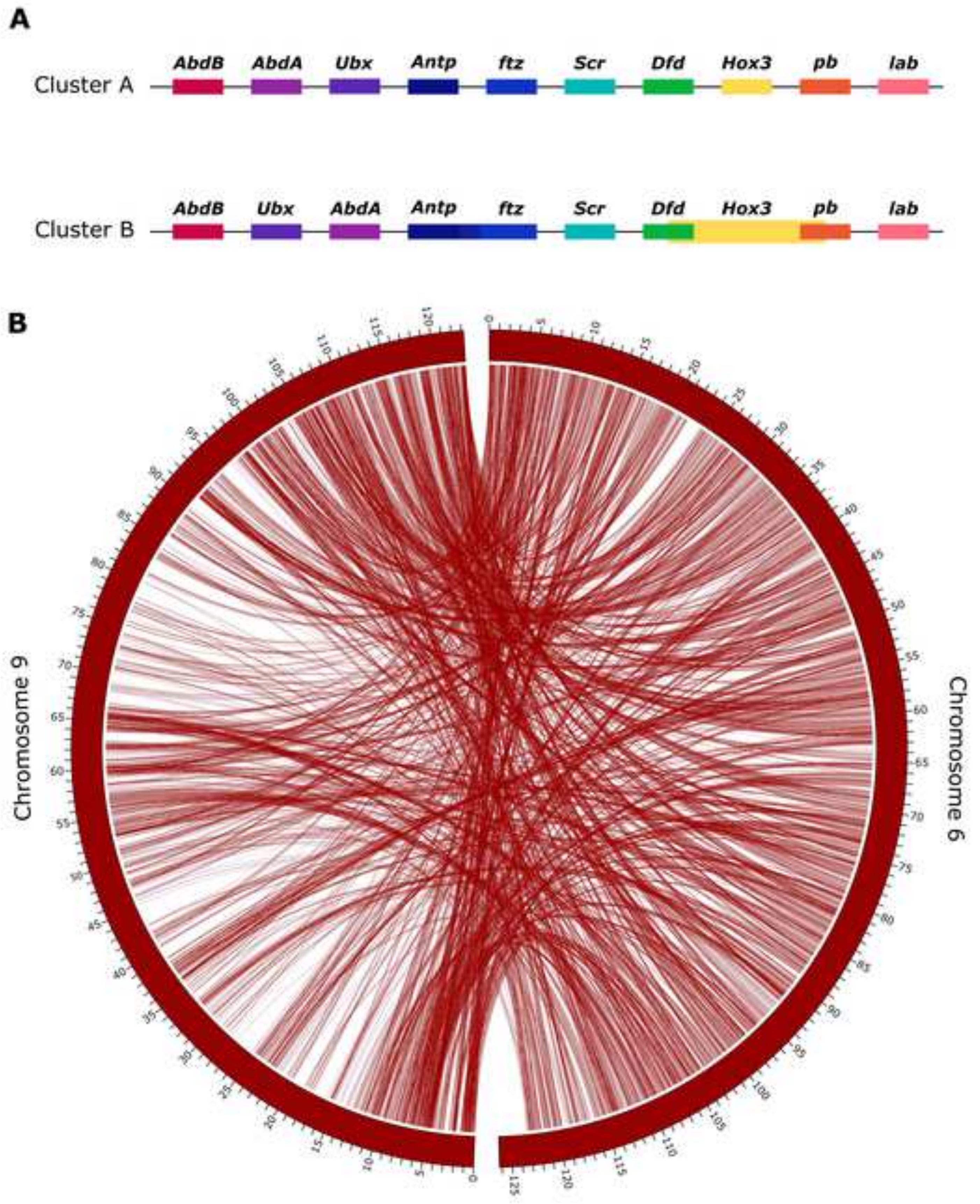

Figure 4

Click here to access/download;Figure;Figure4

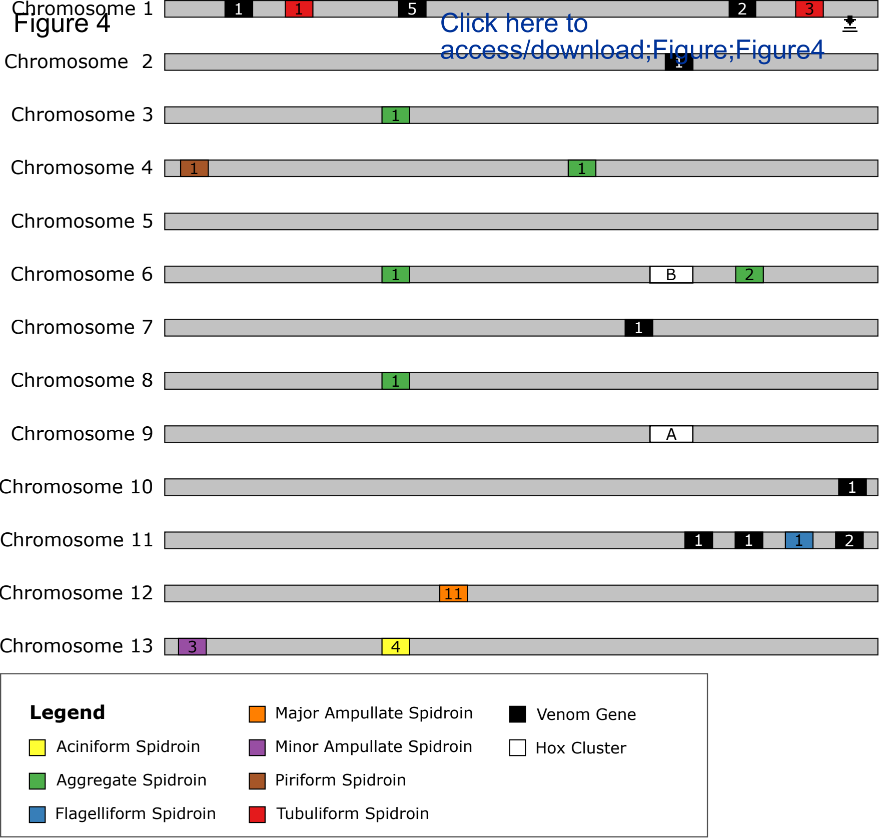

Supplementary Figure S1

Click here to access/download
Supplementary Material
SupplementaryFigureS1_KATplots.png

Supplementary Figure S2

Click here to access/download
Supplementary Material
SupplementaryFigureS2_minorScaffoldLengths.png

Supplementary Figure S3

Click here to access/download
Supplementary Material
SupplementaryFigureS3_RepeatContent.png

Supplementary File S1

Click here to access/download
Supplementary Material
SupplementaryFileS1_hox_blastQuery.fasta

Supplementary File S2

Click here to access/download
Supplementary Material
SupplementaryFileS2_spidroin_blastQuery.fasta

Supplementary File S3

Click here to access/download

Supplementary Material

SupplementaryFileS3_venom_blastQuery.fasta

Supplementary Table S1

Click here to access/download
Supplementary Material
SupplementaryTableS1_SpiderGenomeAssemblies.xlsx

Supplementary Table S2

Click here to access/download
Supplementary Material
SupplementaryTableS2_RepetitiveContentSpiders.xlsx

Supplementary Table S3

Click here to access/download
Supplementary Material
SupplementaryTableS3_hox_blastResults.xlsx

Supplementary Table S4

Click here to access/download
Supplementary Material
SupplementaryTableS4_spidroin_blastResults_revised.xlsx

Supplementary Table S5

Click here to access/download
Supplementary Material
SupplementaryTableS5_venom_blastResults.xlsx
