# Supplementary figures and images for: Chromosome-level reference genome of the European wasp spider Argiope bruennichi: a resource for studies on range expansion and evolutionary adaptation
Source: Gigascience. 2021 Jan 7;10(1):giaa148. doi: 10.1093/gigascience/giaa148 (PMC7788392; doi:10.1093/gigascience/giaa148)

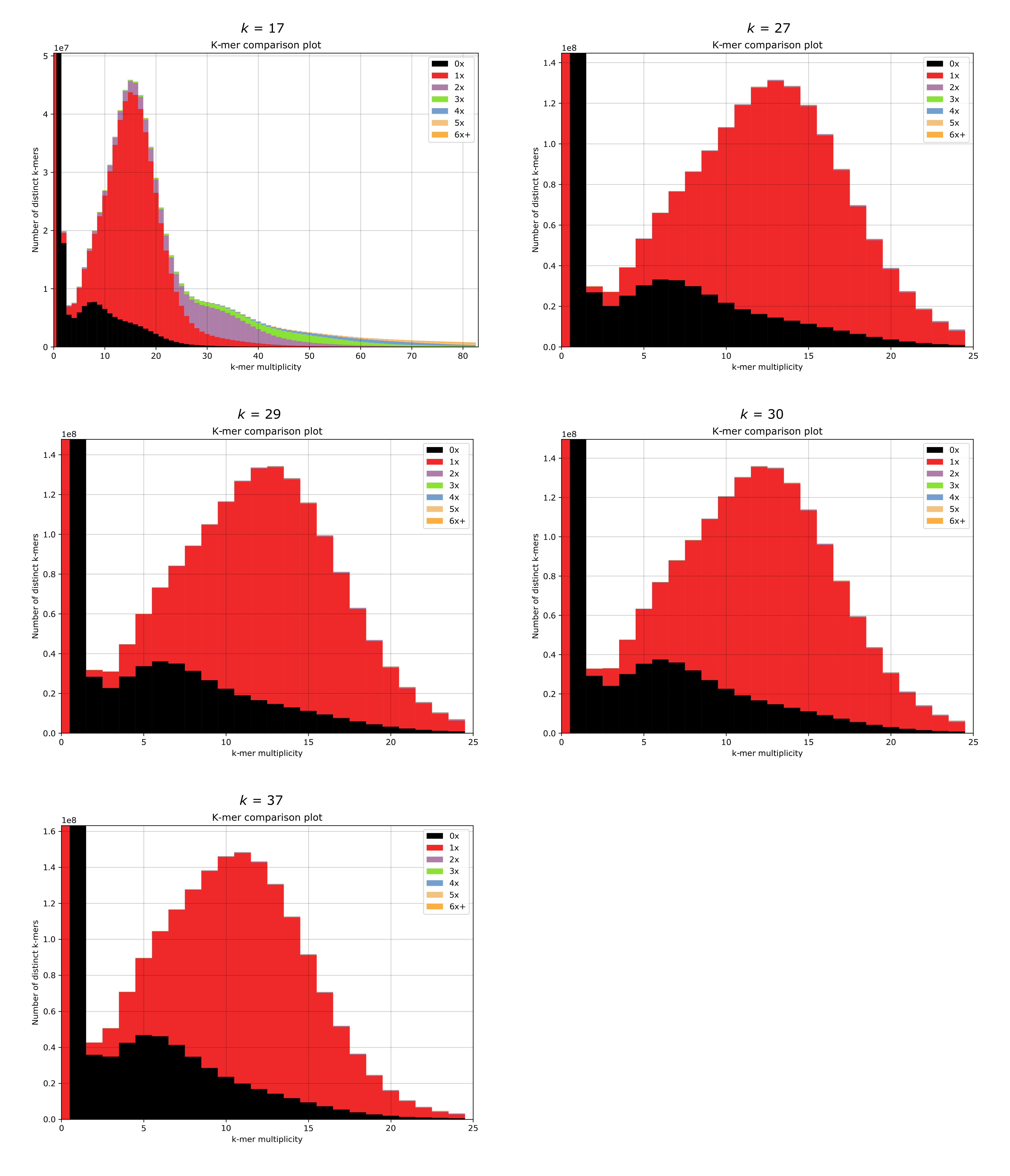

Supplement: giaa148_Supplemental_Files [file giaa148_supplemental_files.zip › SupplementaryFigureS1_KATplots.png]

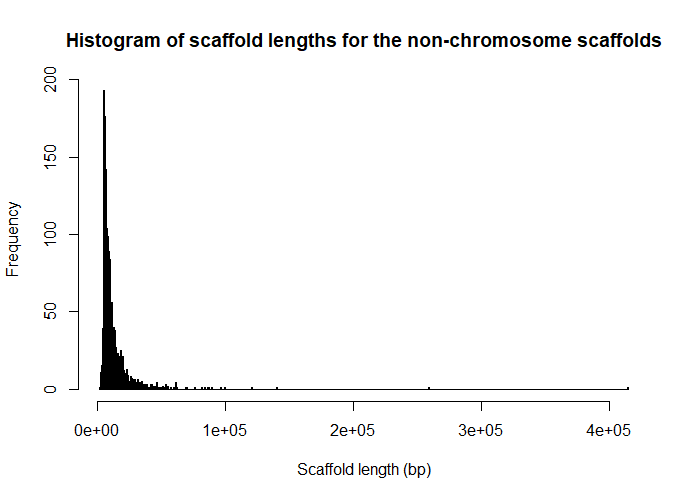

Supplement: giaa148_Supplemental_Files [file giaa148_supplemental_files.zip › SupplementaryFigureS2_minorScaffoldLengths.png]

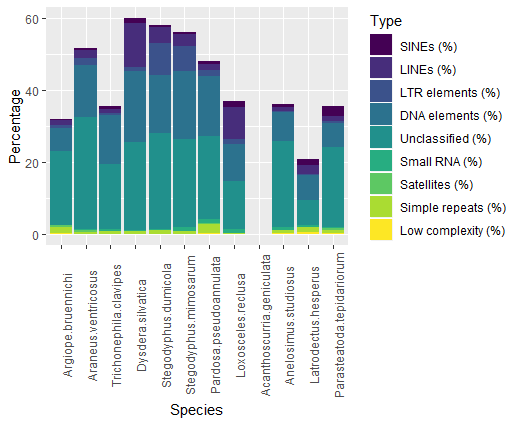

Supplement: giaa148_Supplemental_Files [file giaa148_supplemental_files.zip › SupplementaryFigureS3_RepeatContent.png]
