# Supplementary material for: Chromosome-level reference genome of the European wasp spider Argiope bruennichi: a resource for studies on range expansion and evolutionary adaptation
Source: Gigascience. 2021 Jan 7;10(1):giaa148. doi: 10.1093/gigascience/giaa148 (PMC7788392; doi:10.1093/gigascience/giaa148)
Supplement: giaa148_Response_to_Reviewer_Comments_Original_Submission [file giaa148_response_to_reviewer_comments_original_submission.pdf]

Author's Response To Reviewer Comments

Close

Comments to the editor:

I am re-submitting our data note manuscript entitled, “Chromosome-level reference genome of the European wasp spider Argiope bruennichi: a resource for studies on range expansion and evolutionary adaptation” by Monica M. Sheffer, Anica Hoppe, Henrik Krehenwinkel, Gabriele Uhl, Andreas W. Kuss, Lars Jensen, Corinna Jensen, Rosemary G. Gillespie, Katharina J. Hoff and Stefan Prost (shared last authorship, KJH and SP), following major revision.
Firstly, we would like to thank both reviewers for their insightful comments, which we feel helped us to improve the manuscript substantially. We addressed the specific points of the reviewers in response letters to each of them, below. However, there are some general changes to the manuscript that were not specifically requested, which are outlined here:

1) Formatting of title page and table with author emails (we did not track formatting changes, as this looked very messy, but actual additions of information are tracked)
2) Addition of city to author affiliation information (lines 14-27)
3) We can no longer claim to have published the first chromosome-level genome for an arachnid, as recently six tick genomes have been published at chromosome level. Therefore, we have changed the text to reflect that this is the first chromosome-level genome assembly for a spider (order Araneae) (lines 46-47, 402-403).
4) We rearranged and added one new panel to our assembly completeness figure (figure 2B), which shows the size of the 20 largest scaffolds. This demonstrates that the 13 ‘chromosome’ super scaffolds are dramatically larger than the next largest, and that there are no large missing pieces.
5) According to formatting guidelines of the journal, we removed embedded titles from our figures.
6) In the final version of our assembly, no bacterial or mitochondrial sequences were found, so we adjusted phrasing in lines 224-226 to reflect that.
7) Some small changes to grammar and word choice have also been made.

With the field of arachnid genomics advancing so quickly, we are hopeful that the revisions to our manuscript are satisfactory, as a quick decision would allow us to publish our dataset while it is still the first chromosome-level genome for a spider and will be valued and cited as such.

Sincerely,
Monica M. Sheffer, on behalf of all co-authors

Authors’ reply to the Review Report (Reviewer 1):

We thank Reviewer 1 for the input on and subsequent improvement of our manuscript, and have answered their questions here and amended the manuscript to address them. Changes to the manuscript are given here with line numbers and excerpts from the text, and indicated in the text using track changes. Line numbers correspond to the manuscript with “All Markup” showing in track changes.

Reviewer point one:
Minor point, but the genomes of L. hesperus and L. reclusa have been analyzed, "published" and discussed along with other pilot genomes of the i5k project in a paper by Thomas et al. (2020) in Genome Biology (see: https://genomebiology.biomedcentral.com/articles/10.1186/s13059-019-1925-7) It is more that these species' genomes haven't been published and discussed in their own single genome specific paper. It would be nice to cite the aforementioned paper to credit the i5k work.

Authors’ response:
Thank you for drawing our attention to this oversight. We have updated the text (lines 91-94) and Supplementary Table 1 to reflect the publication of these spider genomes:

“To the best of our knowledge, ten draft spider genomes have been published to date [7,27–33], most of which focus on silk and venom genes, while one discusses whole-genome duplication [7] and the publication of the most recent two focuses on gene content evolution across arthropods [33]. There is one additional, as yet unpublished, spider genome assembly available on NCBI (National Center for Biotechnology Information) (Anelosimus studiosus, accession number: GCA_008297655.1).”

Reviewer point two:
On page 3, line 63 the authors discuss why spider genomes are notoriously difficult to assemble They mention high repeat content , low GC content and long spidroin genes. I was surprised that they did not mention that spider genomes are likely to be highly polymorphic (have high heterozygosity), and the difficulty of assembling heterozygous genomes, and that it is not easy to make inbred lines of spiders. Given the authors specifically pick an individual from a population with low heterozygosity, it seems they recognize this as a problem too, so perhaps they should mention this being part of the problem of assembly.

Authors’ response:
Indeed, this is a challenge that we failed to mention in the introduction; we have added this to the text in lines 100-101:
“Spider genomes are considered notoriously difficult to sequence, assemble, and annotate for a number of factors, including their relatively high repeat content, low guanine cytosine (GC) content, high levels of heterozygosity in the wild [27]...”

Reviewer point three:
The Babb et al. 2017 paper should probably also be cited along with the other references on line 66 (it also provides a comprehensive sense of spidroin gene lengths)

Authors’ response:
Thank you for noticing this omission. We have added the reference to line 102, and additionally added the reference to Kono et al. 2019, which is also relevant here:
“…they possess some extremely long coding genes in the spidroin gene families [28,29,34,35].”

Reviewer point four:
The authors should provide more detail on the library preparation methods for the PacBio genomic DNA libraries prior to sequencing. What was the length of the DNA insert sizes sequenced, what type of size selection methods were employed to restrict the sequencing to large fragments? This is important for people that would like to replicate the methods and maximize the utility of this publication. How long were the movie lengths of the SMRT cells?

Authors’ response:
We included additional information about the library preparation for PacBio sequencing, lines 132-135:
“The DNA was stored at -80°C until library preparation in 2017. The DNA extract was cleaned using a salt:PCI cleaning step, and had a fragment size distribution from 1,300-165,500 bp (peak at 14,002 bp) before size selection. The library was size selected to 15 kilobasepairs (kb) using Pippin prep…”
We do not have any information on the movie lengths, but if this is critical information for the reviewer, we can contact the sequencing facility for more details.
One additional change is to the sequencing year: we noticed in our notes that while we submitted the DNA for library prep and sequencing in 2017, the actual sequencing was performed by the facility in 2018. This has been updated in line 135:
“…and subsequently sequenced in 2018 at the QB3 Genomics facility at the University of California Berkeley on a Pacific Biosciences Sequel I platform (PacBio, Menlo Park, CA, USA) on 10 cells.”

Reviewer point five:
On line 123 can the authors provide the NCBI SRA accession numbers for the Illumina data (from reference 5) used for genome polishing. Was a specific subset of Illumina reads published with reference 5 used for the polishing and if so what geographic population did that individual come from and how many individuals was the data derived from?

Authors’ response:
Thank you for pointing this out, we realized after submission that the accession number was not in the text. We have added the accession number, and information about the sequenced individual in lines 159-161:
“…previously published Illumina paired-end data derived from a single female individual from a

population in Madeira (SRA accession number: ERX533198) [5]…"

Reviewer point six:
I find it a little confusing that the authors do not state the total number scaffolds assembled in the text of the paper but I assume it is listed in Table 1 as 2231 scaffolds. The text says that scaffolding resulted in 13 scaffolds over 1Mb in size. So my interpretation is that there were 2231 scaffolds, and 13 of these were over 1Mb in size. I think the authors should clarify this in the text, in other words most of the genome is in these 13 large pieces but there are still many additional remaining pieces. As a follow up, I think it would be helpful for the authors to discuss what is going on with these remaining pieces (do they contain genes?) and provide more detail on them such as a histogram of the size distribution of the smaller scaffolds, otherwise it is hard to visualize what this data looks like

Authors' response:
This is a very good point. We have added more information on our choice of naming the 13 largest scaffolds as chromosomes, and on the sizes of the lesser scaffolds in the text and a new panel of figure 2 (lines 201-206 (quoted below), Figure 2B), as well as a supplementary histogram which shows all of the lesser scaffold sizes, Supplementary Figure 1. We moved our reference to the bacterial scaffold into this section (de novo genome assembly) as well. We have also provided information on the small number of transcripts predicted on lesser scaffolds in the section on genome annotation (lines 252-253).
"The 13 largest scaffolds are thus henceforth referred to as Chromosomes 1-13, ordered according to size (Figure 2B). The 14th-largest scaffold (Scaffold 839) contained the 16S sequence of a recently discovered, as yet unnamed, bacterial symbiont of A. bruennichi [48]. The remaining 2,217 scaffolds are much smaller, ranging from 1,747-258,743 bp in length (Supplementary Figure 1) and will henceforth be referred to as "lesser scaffolds"."

"The majority of annotated genes fall on the 13 chromosome scaffolds, although 272 transcripts were predicted on the lesser scaffolds."

Reviewer point seven:
I really like the tables and figures of the amount of repetitive DNA content in different spider genomes. Given the earlier statement that spider genomes are difficult to assemble due to their repetitiveness (line 64), I think it would be useful to broaden the context and also compare spider repeat content to that of other arthropods to determine if spiders are an outlier or this was a misconception.

Authors' response:
Thank you for this suggestion; it provides an interesting dimension to the paper. Because some repeat masking programs are prone to under-masking, we included 10 non-spider arthropod species which used RepeatModeler for generating a custom species-specific repeat library and RepeatMasker for masking (as we did) for quantifying repeat content, and downloaded an additional 4 genomes to mask ourselves, representing a broad taxonomic sampling, although narrow within taxa. Repeat masking is very computationally expensive, which is why we chose a small number. The results of our analysis can be found in the text (lines 275-295) and in Table 4, which we extended to include all of the newly-investigated species:
"It is often asserted that the repeat content in spiders is higher in general than in other arthropod groups [i.e. 27]. In order to test this assertion, we looked into the repeat content in genomes of additional arthropod species. We obtained repeat content estimates, for which the repeats were masked using RepeatModeler and RepeatMasker, for three insect species (Bombus terrestris, Drosophila melanogaster and Rhodnius prolixus [71]), and seven tick and mite species (Ixodes persulcatus, Haemaphysalis longicornis, Dermacentor silvarum, Hyalomma asiaticum, Rhipicephalus sanguineus, and Ixodes scapularus [72]). We additionally downloaded the genomes of four more arthropod species, generated custom species-specific repeat libraries with RepeatModeler and masked the genomes with RepeatMasker, to avoid any issues of under- or over masking using other repeat masking programs: a butterfly, Heliconius melpomene [73], a beetle, Tribolium castaneum [74], a millipede, Helicorthomorpha holstii [75], and a scorpion, Centruroides sculpturatus [7,33]. The percentage of total repetitive content for all of these species is presented in Table 4. In general, spiders do have a higher repetitive content than insects, but there is a large range of repetitive content in spiders, compared to which the repetitive content in A. bruennichi is relatively low. All of the selected spider species, aside from Latrodectus hesperus, have higher repetitive content than all other investigated groups, with the exception of ticks and mites, which have very high repetitive content overall (range: 52.6-64.4% repetitive). We conclude from this preliminary investigation that spider genomes, and arachnid genomes generally, do indeed have a higher repeat content than other arthropods."
We hope that this first look, which shows high variability but nonetheless high repeat content in spiders

might inspire further research into the variability in repeat content in other groups. We feel that an in depth analysis of the other arthropod groups is not within the scope of this data note but are happy to discuss this topic further.

Reviewer point eight:
On line 158-159 - very cool that the 14th largest scaffold matched the sequence of a recently discovered symbiont of A. bruennichi. Can the authors says if the entire scaffold matched that of the symbiont or was it a mixture of spider and symbiont genetic material? What was the symbiont species, maybe just name the species?

Authors' response:
At this point, we cannot say if the scaffold is a mix or is solely bacterial sequence. Further work by collaborators will look into the bacterial symbiont in detail, including looking into this scaffold – the bacterium is extremely divergent, in 16S sequence space, from known species, thus we cannot say very much about it currently. We moved this information into the "De novo genome assembly" section, as it fits to the discussion of the non-chromosome "lesser" scaffolds.

Reviewer point nine:
Line 165 - for the published RNA-Seq reads used for genome annotation - can the authors say what tissues, sex and developmental stages these reads came from in this paper to give context to the quality of the evidence for the annotation? Perhaps provide the SRA accession for these reads somewhere?

Authors' response:
We have added the information about life stages, as well as the accession numbers, to the text in lines 234-238:
"Raw reads from previously published transcriptome sequencing data of different life stages: 20 pooled eggs (accession number SRR11861505), 20 pooled first instar spiderlings (accession number SRR11861504), one whole body of an adult female (accession number SRR11861502) and one whole body of an adult male (accession number SRR11861503) [5] were mapped against the repeat-masked assembly…"

Reviewer point ten:
The authors say how many genes were predicted from the genome. Maybe I missed it but I could not find the total number of transcripts/proteins predicted from the genome. I think this should also be listed.

Authors' response:
We have added the predicted transcript number (26,318) to the text in lines 248-249.

Reviewer point eleven:
Can the authors be sure to deposit a fasta file of predicted transcripts and proteins from this genome in NCBI and to report the accession for these in the paper itself? In addition the authors could provide these as supplementary files to maximize the utility of this resource. Can they also provide a link/url in the paper to the UCSC genome browser when it is available?

Authors' response:
The fasta files of predicted transcripts and proteins will be automatically generated by NCBI; our genome is in the final stages of processing, so the files will be available publicly on NCBI when the genome is. However, our files for predicted transcripts and proteins (.aa file for proteins and .codingseq file for transcripts) have been uploaded to gigaDB with the submission of our manuscript, and should thus be accessible – we have added this to the text (lines 254-255):
"The annotation gff3 file and the files containing predicted transcripts and proteins are available on GigaDB."
We have added the URL for the UCSC browser in the section, "Availability of supporting data," although it should now also be publicly searchable on the UCSC genome browser homepage.

Reviewer point twelve:
The authors should also think about if they want to provide their gff file as supplementary , again to maximize utility for the community wanting to understand their annotations.

Authors' response:
Similarly to the point above, the gff3 file is available on GigaDB (line 254-255).

Reviewer point thirteen:
The analyses of the venom and silk genes are very interesting but it is hard to tell what are the number of total venom and silk genes or genome-predicted proteins found or within each category, e.g., how many of each silk gene type or total number of venom genes and the numbers distributed in the islands. This is because (as I interpret it) they report on number of regions on chromosomes where those genes lie, but not the number of genes within those regions. I tried to look further into this by looking at the supplementary blast results, but it is hard to tell because different queries blast to some of the same genomic regions. My point is simply that this information is not easy to find or deduce from the way it's presented.

Authors' response:
Indeed, we struggled with how to present these results, so we appreciate the suggestion about reporting on the number of genes within each island in Figure 3. Originally, we included all matches which passed our filters for E-value and % identity in the supplemental files (thus the confusion with multiple queries blasting to the same region), but have now reduced those matches which map to the same region/gene (lines 322-324 and Supplementary Tables 3-5), and have mentioned in the text and in the figure how many genes we found per silk or venom type on each scaffold (lines 370 -376, 379-381, 390-393).

Reviewer point fourteen:
How well does this assembly perform for the spidroin genes? Are they completely assembled, do they contain Ns, how long are they - what is the length range? This would be another good assessment of the quality of the assembly.

Authors' response:
By manual inspection of the blast hits for spidroin genes in the UCSC genome browser (one can use the "genomebrowser_search" column in Supplementary Table 4 to look at each hit), the assembly in general appears to be high quality with good coverage of long PacBio reads in these areas. However, in some cases, such as for the aciniform spidroin genes, it appears that the annotation may have split the genes into several pieces. In the future, if we or others are interested in the details of the silk genes, manual curation of the annotation could improve the annotation of the silk genes, as the assembly appears complete. As to Ns: many of the spidroin genes contain softmasked repeats, but no Ns.

Reviewer point fifteen:
Great job on an important piece of work!

Authors' response:
Thank you very much for the detail-oriented, helpful, and positive review of our work. Your suggestions helped us look at the manuscript with fresh eyes, and allowed us to improve our reporting of the findings in this genome in a more explicit and (hopefully) more understandable way.

Authors' reply to the Review Report (Reviewer 2)

We thank Reviewer 2 (and their PhD student) for the input on and subsequent improvement of our manuscript, and have answered their questions here and, where necessary, amended the manuscript to address them. Changes to the manuscript are given here with line numbers and excerpts from the text, and indicated in the text using track changes. Line numbers correspond to the manuscript with "All Markup" showing in track changes.

Reviewer points one and four:
One: The manuscript revolves around the presentation of a high-quality chromosome-level assembly, but the evidence supporting the quality of the assembly is a bit sketchy: merely contiguity statistics, BUSCO scores and a contact map. To be convinced by the quality of the assembly, I would need to see a KAT plot (https://kat.readthedocs.io/en/latest/walkthrough.html#genome-assembly-analysis-using-k-mer-spectra - as the Illumina sequencing depth is only 30X, the authors will probably need to play a bit with the k-mer size parameter to generate a satisfying plot in which the peaks are well separated from one another) and a k-mer completeness estimate.

Four: Running KAT (with default parameters) on the data downloaded from the FTP server provided by the authors yielded a genome size estimate of 1.62 Gb. Also, KAT estimated a k-mer completeness of

only 88.9% (for the homozygous peak, which should have a 100% k-mer completeness for a haploid assembly of a diploid genome): this may be due to the 21.5X PacBio coverage used for the assembly being too low for the consensus step to fully correct the sequencing errors, followed by a polishing step with Pilon using an Illumina coverage once again on the lower side (30X). The authors could possibly obtain a better polished assembly with a higher k-mer completeness by performing their polishing using HyPo, which utilizes both PacBio and Illumina data.

Authors' response:
As these two points are related, we have chosen to respond to them together.
We have not seen KAT before and it is indeed a very helpful tool. Thank you for the suggestion. With KAT we now have a much more high-resolution tool to investigate the error profile of the assembly. Given the fact that the pacbio and the Illumina data came from two individuals from different populations, would you still expect a k-mer completeness of close to 100%? We will investigate this and also try HyPo, and if that results in a better kmer match, we will update the submission of the genome on NCBI in the future. However, for the purposes of this data note, we feel that fine-tuning the assembly will not change the downstream results of annotation and genomic architecture, and we hope that you agree. We have mentioned the results of KAT in the manuscript now (lines 192-200), with the explanation that the missing k-mers may be due to sequencing errors remaining in the assembly, or due to the use of individuals from different populations in different years:
"As an additional assessment of assembly quality, we ran the K-mer Analysis Toolkit (KAT v. 2.4.2 , RRID: SCR_016741) [63] comp tool, comparing k-mer content in the Illumina sequencing data to k-mer content in the final assembly. Different values of the parameter k (k =17, 27, 29, 30 and 37) yielded k-mer completeness estimates ranging from 86.55-90.43%. The missing k-mer content in the final assembly may be attributed to the fact that the sequenced individuals came from two different populations, or it may be attributed to errors remaining in the assembly, due to the relatively high error rate and moderate 21.8X coverage of PacBio reads."
Given the mapping rates (see point five below) of the Illumina data for polishing and scaffolding, we think the missing k-mers are more likely due to the use of different individuals, and not due to a high error rate remaining in the assembly.
We generated KAT plots for k values of 17, 27, 29, 30 and 37. Due to memory limitations of our server (due to simultaneously running the synteny analysis; see point 6 below), we were not able to run higher values of k. According to the KAT documentation, the plot for k = 17 in our case is too low. We do not see many differences in the KAT plots between the higher values of k, although there are differences in completeness and genome size estimates depending on the value of k: k = 17 yields a genome size estimate of 1.23 Gb and completeness of 90.43%; k = 27 yields a genome size estimate of 1.62 Gb and completeness of 88.9%; k = 29 yields a genome size estimate of 1.621 Gb and completeness of 88.96%; k = 30 yields a genome size estimate of 1.763 Gb and completeness of 87.6%; k = 30 yields a genome size estimate of 1.83 Gb and completeness of 86.55%. Given these results, the k value of 29 seems most appropriate, although we have included the range of results in the manuscript.
Regarding polishing: during the genome assembly polishing process we tried PacBio polishing using Racon before running Pilon. However, that did not change the BUSCO results compared to the Pilon only approach, so we are not confident that polishing using PacBio reads with another tool will change the assembly substantially.
Therefore, in consideration of the time it would take to compare the assembly and polishing strategies, re-run the scaffolding and annotation, etc., we hope that the assembly in its current state is acceptable, as it remains the most complete spider genome assembly to date.

Reviewer point two:
Also, as the amount of repetitions seems fairly high it would be interesting to see coverage plots (obtained by remapping the PacBio reads on the one hand and the Illumina reads on the other hand on the genome assembly) in order to assess whether some repeated parts have been overcollapsed or (conversely) some haplotypes have not been
properly merged, resulting in artefactual duplications.

Authors' response:
We have included two additional tracks on our UCSC genome browser, which show the Illumina and Pacbio reads mapped onto the assembly. While there certainly seem to be some cases of overcollapsing repeats, it does not seem to be a pervasive problem. We hope that with the addition of this track to the browser, interested readers can look into this in detail.

Reviewer point three:
On line 99 it is mentioned that the genome size was estimated at 1.7 Gb but there is no explanation

regarding this estimation: was it obtained using flow cytometry, or by analyzing the k-mer distribution of Illumina reads?

Authors' response:
Thank you for bringing this to our attention. This estimate was based off of the Animal Genome Size Database, which has densitometry data from the very close relatives Argiope trifasciata and Argiope aurantia. We have added this rationalization to the text. We previously rounded up to 1.7 to not overestimate our coverage estimate if A. bruennichi has a slightly larger genome size than its close relatives. However, we have now added additional investigations into the expected genome size using bioinformatic methods (backmap.pl), which taken together with the data for the other species show 1.675Gb as an appropriate estimate; therefore, we have changed our coverage measurement slightly to reflect this.
We have added all of this information into a new section, "Genome size estimation and coverage" (lines 157-169):
"We estimated the genome size of Argiope bruennichi based on data for closely related species, and bioinformatically based on previously published Illumina paired-end data derived from a single female individual from a population in Madeira (SRA accession number: ERX533198) [5], which we later used for polishing the assembly.
The closely related species A. aurantia and A. trifasciata have genome size estimates based on densitometry data of 1.620 gigabasepairs (Gb) [45] or 1.650 Gb [46] for A. aurantia and 1.690 Gb for A. trifasciata [45,47]. Using the backmap.pl (v. 0.3) pipeline [48–55] on the Illumina data from A. bruennichi [5], we generated a genome size estimate of 1.740 Gb. Averaging these four genome size measurements yields an estimate of 1.675 Gb.
Given this estimate, the PacBio sequencing yielded 21.8X coverage (approximately 36.65 Gb sequenced, with an estimated genome size of 1.675 Gb)"
We also tried using KAT to estimate the genome size, but found that it was extremely sensitive to the chosen value of k, and thus left it out of the estimate. The KAT documentation describes k values from 17 to 63 as generally reasonable. Therefore, we ran KAT comp with k values 17, 27, 29, 30 and 37. Higher values required more memory than we have on our server. With increasing values of k we had increasing genome size estimates (1.23 Gb, 1.62 Gb, 1.621 Gb, 1.763 Gb, 1.83 Gb).

Reviewer point five:
As the PacBio, Illumina and Hi-C data were generated from different individuals collected several years apart, the mapping rates of the Illumina data on the initial PacBio assembly as well as the mapping rate of the the Hi-C data on the polished assembly should be mentioned.

Authors' response:
We have included information on the mapping rates in lines 175-176 and lines 183-184:
"Mapping for the three rounds of polishing resulted in a mapping rate ranging from 92.55-93.69%."
"The sequences from this [the Hi-C] library had a 94.71% mapping rate against the polished assembly."

Reviewer point six:
The part entitled "whole-genome duplication" does not really look into WGS per se but rather only analyzes the duplication of the Hox gene cluster, which could also result from a segmental duplication involving this cluster. Argiope bruennichi being the first chromosome-scale assembly made available for any arachnid, the authors should seize this opportunity to perform a synteny and/or microsynteny analysis at chromosome level in order to check whether they find evidence supporting an actual whole-genome duplication.

Authors' response:
This suggestion is indeed very interesting. We tried to run Satsuma Synteny 2 on our genome, comparing the whole genome to itself, which proved to be too computationally intensive for our server. Therefore, we started by comparing the two hox-containing chromosomes, which do indeed appear to be very orthologous (Screenshot of MizBee viewer available upon request), suggesting at least duplication of these complete chromosomes, if not the whole genome.
To look for further evidence, we tried running a single chromosome against the whole genome to find the duplicate of that chromosome. This analysis ran for more than three weeks before it used all of the memory of our server and "core dumped". We then considered going pairwise, comparing one chromosome to another through all possible comparisons, however the comparison of the two Hox-containing scaffolds above took more than 5 days and all of our server's memory to run. The pairwise analysis of all remaining chromosomes, if they took the same time, would have taken more time than we were allotted for the revision, and would tie up all of our computational resources for other projects

during that time. If the reviewer has a suggestion for another tool for synteny analysis (perhaps something lighter weight in terms of computation), we would welcome it, as we find this very interesting in general.
Because we are unable to provide evidence of WGD, and only of the two Hox-containing chromosomes, we have changed our manuscript to report on "Hox cluster duplication" (lines 54, 328, 356-361, 449, 725-727) instead of whole-genome duplication:
"It is possible that Hox Cluster B in spiders has changed or lost functionality following the proposed ancestral WGD event. The presence of two Hox clusters in our assembly is suggestive, but not evidence, of WGD in A. bruennichi, as it could have also arisen from duplication of only the Hox-containing chromosome; future studies can capitalize on the now-available chromosome-level assemblies for several groups (e.g. horseshoe crabs, ticks, and our spider) [72,88] to do more detailed analyses of duplication across chelicerates."
We feel that with the publication of this genome, as well as the chromosome-level genomes of the horseshoe crab and a handful of tick species which have been published since we submitted our manuscript, the stage is set for a chelicerate-wide analysis of WGD. This is not possible for us given the computational resources needed, but we look forward to sharing our data with others so that it can be used for this purpose. Indeed, this is why we chose the format of a data note for fast dissemination of our data.

Reviewer point seven:
-line 38: "Arachnids" should be spelled "arachnids

Authors' response:
Thank you for bringing this to our attention; it has been fixed in the text (line 49).

Reviewer point eight:
- lines 38-39: "whole-genome duplication" is normally with a hyphen

Authors' response:
Thank you for bringing this to our attention. We have fixed this in the indicated lines in the abstract, and throughout the text (lines 50, 93, 303, 329, 433, 726).

Reviewer point nine:
- lines 53 and 230: I checked reference 7 and it does not really seem to support the assertion that "chromosome-level genome assembly would greatly increase the potential for inference on evolutionary adaptation and modes of speciation" (there is no discussion about the need for a chromosome-level genome assembly in that paper)

Authors' response:
This reference (formerly reference 7, now reference 8) shows how chromosomal rearrangements play a role in speciation in spiders. While the authors do not directly make the argument that one needs a genome assembly for this purpose, it nonetheless supports the idea that information on chromosomal conformation (in this case assessed at a karyotype level) is important to understand speciation and adaptation. Chromosomal genome assemblies are another tool to complement and expand our understanding of the role of chromosomal rearrangements in speciation.
Since we realized that the argumentation in the introduction could be stronger, clearer, and better supported by references to the literature, we have rewritten these paragraphs (lines 63-90) to provide a stronger argument, and added more references that rely on genomic sequence data, but have left this reference in due to its relevance for the taxonomic group:
"With regards to adaptation, work on cobweb spiders (Theridiidae) has revealed a whole-genome duplication that may facilitate diversification [7], with other studies highlighting a key role of tandem duplication and neofunctionalization of genes in the diversification and specialization of spider silks [8] and venoms [9]. A key aspect that has been missing from studies to date is the role of genome organization in facilitating or impeding adaptation as there have been no studies to date on spiders that have provided a chromosomal framework for the genome.
Understanding the chromosomal organization of a genome is critical for identification of processes underlying divergence between populations, adaptation, and speciation. Indeed, the potential role of chromosomal reorganization in species formation has long been the subject of debate, in particular in Drosophila species where polytene chromosomes allowed early visualization of chromosomal rearrangements [10]. Among spiders, karyotype data are still used to identify changes in chromosomes associated with speciation [11]. With the advent of detailed genomic data, there has been renewed focus on the role that structural variants in the genome can play as drivers of adaptation and speciation,

associated with translocations, fusions, and inversions [12], as well as with admixture and associated demographic changes [13]. Recent data from sister species of the genus Drosophila suggest that the establishment of inversion polymorphisms within isolated and/or heterogeneous environments may well set the stage for species formation [14]. In order to develop a broader understanding of the role of structural variation in adaptation and speciation [15–22], we need chromosome-level genomes that provide the ability to map the order of genes, define chromosomal gene neighborhoods, and identify potential genomic islands of differentiation [23–26]."

Reviewer point ten:
- line 305: I could not find the "Wasp spider hub" on the UCSC genome browser, please provide a direct link

Authors' response:
Our apologies for this, as public listing of the hub was delayed; the hub is now publicly listed at the UCSC Genome browser, and we have added the direct link to the accessibility of data section (lines 416-417).

Reviewer point eleven:
- figure 3b could be removed as it does not bring much relevant information that is not already present in the text.

Authors' response:
While we understand the reviewer's point that figure 3B could be seen as redundant relative to the text, we see the figure as a complement to more easily follow the text and place the results for the different gene groups into context with one another. If it is acceptable to the reviewers and editors, we would like to leave figure 3B in.

Close
