# Supplementary material for: Chromosome-level reference genome of the European wasp spider Argiope bruennichi: a resource for studies on range expansion and evolutionary adaptation
Source: Gigascience. 2021 Jan 7;10(1):giaa148. doi: 10.1093/gigascience/giaa148 (PMC7788392; doi:10.1093/gigascience/giaa148)
Supplement: giaa148_Response_to_Reviewer_Comments_Revision_1 [file giaa148_response_to_reviewer_comments_revision_1.pdf]

Author's Response To Reviewer Comments

Close

Dear Editor:

I am submitting the revised version our data note manuscript entitled, “Chromosome-level reference genome of the European wasp spider Argiope bruennichi: a resource for studies on range expansion and evolutionary adaptation” by Monica M. Sheffer, Anica Hoppe, Henrik Krehenwinkel, Gabriele Uhl, Andreas W. Kuss, Lars Jensen, Corinna Jensen, Rosemary G. Gillespie, Katharina J. Hoff and Stefan Prost (shared last authorship), with minor changes.

We have addressed the remaining comments from the second reviewer, following the latest round of review:
- We have included the KAT plots as a supplementary figure (Supplementary Figure S1, line 188), and removed our sentence suggesting that the use of different individuals may have been the reason for the missing kmer content (lines 188-192), as the reviewer found this unlikely.
- We have changed Figure 3 to focus solely on Hox duplication: Figure 3A, colinearity of the Hox genes, remains the same; Figure 3B now contains the circular chromosome viewer depicting conserved syntenic blocks between the Hox-containing chromosomes. What was formerly Figure 3B (displaying the location of gene families within the genome) now stands alone as Figure 4. We have updated the figure legends (lines 727-743) to correspond to these changes, and added a few sentences about the synteny analysis in the main text (lines 345-356).
- We have addressed all of the changes to word choice and sentence structure that the reviewer requested, and fixed the references as he indicated.

In addition to those reviewer-requested changes above, we have made a few in addition:
- We have included the citation to our GigaDB dataset, as provided by the data curators, and included the information on available file types in our “Availability of supporting data” section.
- We have corrected the estimation of ~30X coverage of Illumina reads (we based this on the coverage provided in the publication of those reads, but have now calculated the coverage ourselves) in the abstract and throughout the text (lines 40, 159-160, 165, 190). The coverage is in fact 19.8X.
- We have numbered the supplementary files with an “S” in front of the number, as is the norm in the journal, i.e. Supplementary Figure S1 instead of Supplementary Figure 1.
- In some references, the publisher was missing. We have added this throughout the references.
- We have removed the legend of Figure 2C, as it was unnecessary, and removed the boxes around the figures within Figure 2, as they did not fit the aesthetic of the other figures.
- Slight changes to punctuation and sentence structure were also made.

Per our previous communication, we would like to expedite the process of publication of our manuscript as much as possible, so that we can pay the Article Processing Charges with the remainder of our 2020 budget.

We would like to, once again, express our gratitude for the thorough work of both reviewers, and the efficient and helpful work of the handling editor, which have made this process very positive and constructive. We look forward to the publication of our article.

Sincerely,
Monica M. Sheffer, on behalf of all co-authors

Close
