# Supplementary material for: Chromosome-level reference genome of the European wasp spider Argiope bruennichi: a resource for studies on range expansion and evolutionary adaptation
Source: Gigascience. 2021 Jan 7;10(1):giaa148. doi: 10.1093/gigascience/giaa148 (PMC7788392; doi:10.1093/gigascience/giaa148)
Supplement: giaa148_Reviewer_1_Report_Original_Submission — Jessica Garb -- 7/7/2020 Reviewed [file giaa148_reviewer_1_report_original_submission.pdf]

Reviewer Report

Title: Chromosome-level reference genome of the European wasp spider Argiope bruennichi: a resource for studies on range expansion and evolutionary adaptation

Version: Original Submission **Date:** 7/7/2020

Reviewer name: Jessica Garb

Reviewer Comments to Author:

This Data Note describes the first chromosome level assembly of a spider genome (Argiope bruennichi), which represents a major advance in spider genomics. In the context of the Data Note guidelines, the work is well-suited. The paper presents a significant dataset that will be highly useful for a large community of scientists. The paper is well written and easy to understand. The analyses presented are thorough and the figures and tables are easy to interpret. The work represents a substantial advance in the field considering the past difficulties associated with assembling spider genomes and will pave the way for future studies using this genome as a reference across multiple fields.

I have the following comments, questions and clarifications I would like the authors to address in the manuscript:

1) Minor point, but the genomes of L. hesperus and L. reclusa have been analyzed, "published" and discussed along with other pilot genomes of the i5k project in a paper by Thomas et al. (2020) in Genome Biology (see: https://genomebiology.biomedcentral.com/articles/10.1186/s13059-019-1925-7) It is more that these species' genomes haven't been published and discussed in their own single genome specific paper. It would be nice to cite the aforementioned paper to credit the i5k work.

2) On page 3, line 63 the authors discuss why spider genomes are notoriously difficult to assemble They mention high repeat content , low GC content and long spidroin genes. I was surprised that they did not mention that spider genomes are likely to be highly polymorphic (have high heterozygosity), and the difficulty of assembling heterozygous genomes, and that it is not easy to make inbred lines of spiders. Given the authors specifically pick an individual from a population with low heterozygosity, it seems they recognize this as a problem too, so perhaps they should mention this being part of the problem of assembly.

3) The Babb et al. 2017 paper should probably also be cited along with the other references on line 66 (it also provides a comprehensive sense of spidroin gene lengths)

4) The authors should provide more detail on the library preparation methods for the PacBio genomic DNA libraries prior to sequencing. What was the length of the DNA insert sizes sequenced, what type of size selection methods were employed to restrict the sequencing to large fragments? This is important for people that would like to replicate the methods and maximize the utility of this publication. How long were the movie lengths of the SMRT cells?

5) On line 123 can the authors provide the NCBI SRA accession numbers for the Illumina data (from reference 5) used for genome polishing. Was a specific subset of Illumina reads published with reference 5 used for the polishing and if so what geographic population did that individual come from and how many individuals was the data derived from?

6) I find it a little confusing that the authors do not state the total number scaffolds assembled in the text of the paper but I assume it is listed in Table 1 as 2231 scaffolds. The text says that scaffolding resulted in 13 scaffolds over 1Mb in size. So my interpretation is that there were 2231 scaffolds, and 13 of these were over 1Mb in size. I think the authors should clarify this in the text, in other words most of the genome is in these 13 large pieces but there are still many additional remaining pieces. As a follow up, I think it would be helpful for the authors to discuss what is going on with these remaining pieces (do they contain genes?) and provide more detail on them such as a histogram of the size distribution of the smaller scaffolds, otherwise it is hard to visualize what this data looks like.

7) I really like the tables and figures of the amount of repetitive DNA content in different spider genomes. Given the earlier statement that spider genomes are difficult to assemble due to their repetitiveness (line 64), I think it would be useful to broaden the context and also compare spider repeat content to that of other arthropods to determine if spiders are an outlier or this was a misconception.

8) On line 158-159 - very cool that the 14th largest scaffold matched the sequence of a recently discovered symbiont of A. bruennichi. Can the authors says if the entire scaffold matched that of the symbiont or was it a mixture of spider and symbiont genetic material? What was the symbiont species, maybe just name the species?

9) Line 165 - for the published RNA-Seq reads used for genome annotation - can the authors say what tissues, sex and developmental stages these reads came from in this paper to give context to the quality of the evidence for the annotation? Perhaps provide the SRA accession for these reads somewhere?

10) The authors say how many genes were predicted from the genome. Maybe I missed it but I could not find the total number of transcripts/proteins predicted from the genome. I think this should also be listed.

11) Can the authors be sure to deposit a fasta file of predicted transcripts and proteins from this genome in NCBI and to report the accession for these in the paper itself? In addition the authors could provide these as supplementary files to maximize the utility of this resource. Can they also provide a link/url in the paper to the UCSC genome browser when it is available?

12) The authors should also think about if they want to provide their gff file as supplementary, again to maximize utility for the community wanting to understand their annotations.

13) The analyses of the venom and silk genes are very interesting but it is hard to tell what are the number of total venom and silk genes or genome-predicted proteins found or within each category, e.g., how many of each silk gene type or total number of venom genes and the numbers distributed in the islands. This is because (as I interpret it) they report on number of regions on chromosomes where those genes lie, but not the number of genes within those regions. I tried to look further into this by looking at the supplementary blast results, but it is hard to tell because different queries blast to some of the same genomic regions. My point is simply that this information is not easy to find or deduce from the way it's presented.

14) How well does this assembly perform for the spidroin genes? Are they completely assembled, do they contain Ns, how long are they - what is the length range? This would be another good assessment of the quality of the assembly.

15) Great job on an important piece of work!

Level of Interest

Please indicate how interesting you found the manuscript: Choose an item.

Quality of Written English

Please indicate the quality of language in the manuscript: Choose an item.

Declaration of Competing Interests

Please complete a declaration of competing interests, considering the following questions:

- Have you in the past five years received reimbursements, fees, funding, or salary from an organisation that may in any way gain or lose financially from the publication of this manuscript, either now or in the future?
- Do you hold any stocks or shares in an organisation that may in any way gain or lose financially from the publication of this manuscript, either now or in the future?
- Do you hold or are you currently applying for any patents relating to the content of the manuscript?
- Have you received reimbursements, fees, funding, or salary from an organization that holds or has applied for patents relating to the content of the manuscript?
- Do you have any other financial competing interests?
- Do you have any non-financial competing interests in relation to this paper?

If you can answer no to all of the above, write 'I declare that I have no competing interests' below. If your reply is yes to any, please give details below.

I declare that I have no competing interests.

I agree to the open peer review policy of the journal. I understand that my name will be included on my report to the authors and, if the manuscript is accepted for publication, my named report including any attachments I upload will be posted on the website along with the authors' responses. I agree for my report to be made available under an Open Access Creative Commons CC-BY license (http://creativecommons.org/licenses/by/4.0/). I understand that any comments which I do not wish to be included in my named report can be included as confidential comments to the editors, which will not be published.

Choose an item.

To further support our reviewers, we have joined with Publons, where you can gain additional credit to further highlight your hard work (see: https://publons.com/journal/530/gigascience). On publication of

this paper, your review will be automatically added to Publons, you can then choose whether or not to claim your Publons credit. I understand this statement.

Yes Choose an item.
