# Supplementary material for: Chromosome-level reference genome of the European wasp spider Argiope bruennichi: a resource for studies on range expansion and evolutionary adaptation
Source: Gigascience. 2021 Jan 7;10(1):giaa148. doi: 10.1093/gigascience/giaa148 (PMC7788392; doi:10.1093/gigascience/giaa148)
Supplement: giaa148_Reviewer_2_Report_Original_Submission — Jean-FranÃ§ois Flot -- 8/23/2020 Reviewed [file giaa148_reviewer_2_report_original_submission.pdf]

Reviewer Report

Title: Chromosome-level reference genome of the European wasp spider Argiope bruennichi: a resource for studies on range expansion and evolutionary adaptation

Version: Original Submission **Date:** 8/23/2020

Reviewer name: Jean-FranÃ§ois Flot

Reviewer Comments to Author:

I was excited to read about the first chromosome-scale spider genome, but this manuscript will still require a little bit of work before I can recommend it for publication.
Major concerns:
The manuscript revolves around the presentation of a high-quality chromosome-level assembly, but the evidence supporting the quality of the assembly is a bit sketchy: merely contiguity statistics, BUSCO scores and a contact map. To be convinced by the quality of the assembly, I would need to see a KAT plot (https://kat.readthedocs.io/en/latest/walkthrough.html#genome-assembly-analysis-using-k-mer-spectra - as the Illumina sequencing depth is only 30X, the authors will probably need to play a bit with the k-mer size parameter to generate a satisfying plot in which the peaks are well separated from one another) and a k-mer completeness estimate. Also, as the amount of repetitions seems fairly high it would be interesting to see coverage plots (obtained by remapping the PacBio reads on the one hand and the Illumina reads on the other hand on the genome assembly) in order to assess whether some repeated parts have been overcollapsed or (conversely) some haplotypes have not been properly merged, resulting in artefactual duplications.
On line 99 it is mentioned that the genome size was estimated at 1.7 Gb but there is no explanation regarding this estimation: was it obtained using flow cytometry, or by analyzing the k-mer distribution of Illumina reads?
Running KAT (with default parameters) on the data downloaded from the FTP server provided by the authors yielded a genome size estimate of 1.62 Gb. Also, KAT estimated a k-mer completeness of only 88.9% (for the homozygous peak, which should have a 100% k-mer completeness for a haploid assembly of a diploid genome): this may be due to the 21.5X PacBio coverage used for the assembly being too low for the consensus step to fully correct the sequencing errors, followed by a polishing step with Pilon using an Illumina coverage once again on the lower side (30X). The authors could possibly obtain a better polished assembly with a higher k-mer completeness by performing their polishing using HyPo, which utilizes both PacBio and Illumina data.
As the PacBio, Illumina and Hi-C data were generated from different individuals collected several years apart, the mapping rates of the Illumina data on the initial PacBio assembly as well as the mapping rate of the the Hi-C data on the polished assembly should be mentioned.
The part entitled "whole-genome duplication" does not really look into WGS per se but rather only analyzes the duplication of the Hox gene cluster, which could also result from a segmental duplication involving this cluster. Argiope bruennichi being the first chromosome-scale assembly made available for any arachnid, the authors should seize this opportunity to perform a synteny and/or microsynteny

analysis at chromosome level in order to check whether they find evidence supporting an actual whole-genome duplication.
Minor concerns:
- line 38: "Arachnids" should be spelled "arachnids";
- lines 38-39: "whole-genome duplication" is normally with a hyphen;
- lines 53 and 230: I checked reference 7 and it does not really seem to support the assertion that "chromosome-level genome assembly would greatly increase the potential for inference on evolutionary adaptation and modes of speciation" (there is no discussion about the need for a chromosome-level genome assembly in that paper);
- line 305: I could not find the "Wasp spider hub" on the UCSC genome browser, please provide a direct link;
- figure 3b could be removed as it does not bring much relevant information that is not already present in the text.

Jean-FranÃ§ois Flot (with contributions from PhD student NadÃ¨ge Guiglielmoni)
UniversitÃ© libre de Bruxelles, Belgium

Level of Interest

Please indicate how interesting you found the manuscript: Choose an item.

Quality of Written English

Please indicate the quality of language in the manuscript: Choose an item.

Declaration of Competing Interests

Please complete a declaration of competing interests, considering the following questions:

- Have you in the past five years received reimbursements, fees, funding, or salary from an organisation that may in any way gain or lose financially from the publication of this manuscript, either now or in the future?
- Do you hold any stocks or shares in an organisation that may in any way gain or lose financially from the publication of this manuscript, either now or in the future?
- Do you hold or are you currently applying for any patents relating to the content of the manuscript?
- Have you received reimbursements, fees, funding, or salary from an organization that holds or has applied for patents relating to the content of the manuscript?
- Do you have any other financial competing interests?
- Do you have any non-financial competing interests in relation to this paper?

If you can answer no to all of the above, write 'I declare that I have no competing interests' below. If your reply is yes to any, please give details below.

I declare that I have no competing interests

I agree to the open peer review policy of the journal. I understand that my name will be included on my report to the authors and, if the manuscript is accepted for publication, my named report including any attachments I upload will be posted on the website along with the authors' responses. I agree for my report to be made available under an Open Access Creative Commons CC-BY license (http://creativecommons.org/licenses/by/4.0/). I understand that any comments which I do not wish to be included in my named report can be included as confidential comments to the editors, which will not be published.

Choose an item.

To further support our reviewers, we have joined with Publons, where you can gain additional credit to further highlight your hard work (see: https://publons.com/journal/530/gigascience). On publication of this paper, your review will be automatically added to Publons, you can then choose whether or not to claim your Publons credit. I understand this statement.

Yes Choose an item.
