# Supplementary material for: Chromosome-level reference genome of the European wasp spider Argiope bruennichi: a resource for studies on range expansion and evolutionary adaptation
Source: Gigascience. 2021 Jan 7;10(1):giaa148. doi: 10.1093/gigascience/giaa148 (PMC7788392; doi:10.1093/gigascience/giaa148)
Supplement: giaa148_Reviewer_2_Report_Revision_1 — Jean-FranÃ§ois Flot -- 11/10/2020 Reviewed [file giaa148_reviewer_2_report_revision_1.pdf]

Reviewer Report

Title: Chromosome-level reference genome of the European wasp spider Argiope bruennichi: a resource for studies on range expansion and evolutionary adaptation

Version: Revision 1 **Date:** 11/10/2020

Reviewer name: Jean-FranÃ§ois Flot

Reviewer Comments to Author:

The authors thoroughly revised their article and took into account satisfactorily all my comments. I look forward to seeing this nice piece of work published in GigaScience.
Although I still find the k-mer completeness of their assembly quite low (to the author's question "Given the fact that the pacbio and the Illumina data came from two individuals from different populations, would you still expect a k-mer completeness of close to 100%?" I reply that, if the polishing is performed correctly and the same set of Illumina reads is used for the final polishing and for calculating the k-mer completeness, then one should indeed expect a k-mer completeness close to 100%), improving it would undoubtedly require lots of additional time, effort and resources for a minimal enhancement: since the short-read mapping rates reported by the authors are OK, my guess is that the missing k-mers are mostly located in the repeated parts of the genome (possibly because of cross-mapping of the short Illumina reads during the polishing step) and do not really pose a problem for downstream analyses. Still, it would be interesting to include the KAT plots as supplementary data to the paper.
The interesting analysis of microsynteny between the two Hox-containing chromosomes mentioned by the authors in their response to reviewers should be included in the article, together with a figure showing the colinearity between these two chromosomes. Even if this analysis concerns only one pair of chromosome, such chromosome-scale conservation of synteny is a very significant observation that corroborates the microsynteny analysis in Schwager et al. and goes one step further towards confirming that arachnopulmonates underwent a whole-genome duplication. Moreover, if this analysis is not included in the paper then the rationale for the statement "The presence of two Hox clusters in our assembly is suggestive, but not evidence, of WGD in A. bruennichi, as it could have also arisen from duplication of only the Hox-containing chromosome" (lines 356-358) is unclear (it could also have resulted from the sole duplication of the Hox cluster, not of the whole Hox-containing chromosome).
In addition to these two main comments, I have a few minor suggestions/corrections listed below:
- line 163: rather than just "densitometry", it would be better to specify "Feulgen densitometry";
- line 176: "ranging from 92.55-93.69%" would be better spelled as "ranging from 92.55 to 93.69%";
- lines 194-194: "comparing k-mer content in the Illumina sequencing data to k-mer content in the final assembly" -> "comparing the k-mer content in the Illumina sequencing data to the k-mer content in the final assembly";
- line 330: "a whole-genome duplication has occurred in the ancestor of scorpions and spiders" -> "a whole-genome duplication occurred in the ancestor of scorpions and spiders";
- line 344 and following: "collinear" means "lying on or passing through the same straight line" (https://www.merriam-webster.com/dictionary/collinear), whereas "colinear" means "having

corresponding parts arranged in the same linear order" (https://www.merriam-webster.com/dictionary/colinear). If one writes that "genes A, B, C and D, E, F are collinear" it means that A, B, C, D, E and F occur on the same chomosome, whereas if one writes that "genes A, B, C and D, E, F are colinear" it means that A, B, C on the one hand and D, E, F on other hand occur in the same order on different chromosomes. Hence, the spelling "colinear" is more appropriate here;
- lines 358-359: "future studies can capitalize" -> "future studies will be able to capitalize" ;
- lines 376 and 378: "chitin binding domain" -> "chitin-binding domain";
- lines 476-478: ref. 8 is in title case while all other references are in sentence case;
- line 484: "Drosophila" should be in italics;
- lines 578-579: "Argiope" should be in italics, and the book title "Cryptic Female Choice Arthropods Patterns, Mech Prospec" should rather not be abbreviated: "Cryptic Female Choice in Arthropods: Patterns, Mechanisms and Prospects";
- lines 593-594: ref. 48 is in title case, please put it in sentence case as the other references;
- lines 640-641: please put ref. 68 in sentence case;
- lines 646-651: please put refs. 71 and 72 in sentence case; and
- lines 702-704: please put ref. 92 in sentence case.

Jean-FranÃ§ois Flot
UniversitÃ© libre de Bruxelles (ULB), Belgium

Level of Interest

Please indicate how interesting you found the manuscript: Choose an item.

Quality of Written English

Please indicate the quality of language in the manuscript: Choose an item.

Declaration of Competing Interests

Please complete a declaration of competing interests, considering the following questions:

- Have you in the past five years received reimbursements, fees, funding, or salary from an organisation that may in any way gain or lose financially from the publication of this manuscript, either now or in the future?
- Do you hold any stocks or shares in an organisation that may in any way gain or lose financially from the publication of this manuscript, either now or in the future?
- Do you hold or are you currently applying for any patents relating to the content of the manuscript?
- Have you received reimbursements, fees, funding, or salary from an organization that holds or has applied for patents relating to the content of the manuscript?
- Do you have any other financial competing interests?

- Do you have any non-financial competing interests in relation to this paper?

If you can answer no to all of the above, write 'I declare that I have no competing interests' below. If your reply is yes to any, please give details below.

I declare that I have no competing interests

I agree to the open peer review policy of the journal. I understand that my name will be included on my report to the authors and, if the manuscript is accepted for publication, my named report including any attachments I upload will be posted on the website along with the authors' responses. I agree for my report to be made available under an Open Access Creative Commons CC-BY license (http://creativecommons.org/licenses/by/4.0/). I understand that any comments which I do not wish to be included in my named report can be included as confidential comments to the editors, which will not be published.

Choose an item.

To further support our reviewers, we have joined with Publons, where you can gain additional credit to further highlight your hard work (see: https://publons.com/journal/530/gigascience). On publication of this paper, your review will be automatically added to Publons, you can then choose whether or not to claim your Publons credit. I understand this statement.

Yes Choose an item.
